# Acute circulating tumor DNA dynamics during and after systemic therapy initiation for advanced triple-negative breast cancer

**DOI:** 10.1038/s41523-026-00953-w

**Published:** 2026-04-16

**Authors:** Briana To, Vishnu Prasath, Deloris Veney, Christian Rolfo, Viktor Adalsteinsson, William E. Carson, Robert Wesolowski, Daniel Stover, Dionisia Quiroga

**Affiliations:** 1 The Ohio State University College of Medicine, Columbus, OH USA; 2 Division of Medical Oncology, Department of Internal Medicine, The Ohio State University Comprehensive Cancer Center, Columbus, OH USA; 3 Broad Institute of Harvard, Cambridge, MA USA; 4 Massachusetts Institute of Technology, Cambridge, MA USA; 5 Division of Surgical Oncology, Department of Surgery, The Ohio State University Comprehensive Cancer Center, Columbus, OH USA; 6 Stefanie Spielman Comprehensive Breast Center, Columbus, OH USA

**Keywords:** Cancer, Oncology

## Abstract

Circulating tumor DNA (ctDNA) ‘tumor fraction’ (TF) may correlate with therapy response, but little is known regarding TF dynamics immediately after therapy initiation. Plasma samples from a single-arm clinical trial enrolling patients with triple-negative breast cancer were collected at a variety of time points following infusion of onalespib (day -7), paclitaxel (day 1), and onalespib + paclitaxel (day 8). 313 samples from 14 patients underwent shallow whole genome sequencing and TF determination. The primary objective was to evaluate TF change from pre-infusion to 6 h and 24 h post-infusion. There was significant TF decline from pre-infusion to 6 h for paclitaxel (p = 0.03) but no change for onalespib or onalespib+paclitaxel at 6hl. There was no ctDNA TF surge within minutes to 24 h of onalespib, paclitaxel, or combination, despite an overall decline in TF during the first therapy cycle. However, there was significant decline in TF from pre-infusion to D9 (16% to 6.5%, p = 0.004). These findings support further research on ctDNA dynamics immediately after therapy initiation.

## Introduction

As tumor cells undergo cell death, fragments of DNA are released into the bloodstream, termed ‘circulating tumor DNA’ (ctDNA). The proportion of tumor DNA in circulation (‘tumor fraction’ [TF]) has been shown to be prognostic in metastatic breast cancers and many other malignancies^1–3^, and ctDNA monitoring has an emerging role as a minimally invasive tool to evaluate therapeutic response. There is evidence that early dynamic changes in ctDNA levels during systemic anti-cancer therapy may provide insight into treatment response even before standard imaging changes are evident, making it a promising approach^4–9^.

While there has been significant progress toward elucidating the role of ctDNA as a prognostic and therapeutic biomarker, there is still a poor understanding of early ctDNA dynamics in response to treatment. Available data suggest that the half-life of ctDNA is only 30 minutes to 2 h, indicating relatively rapid degradation and suggesting that ctDNA TF changes could be detectable in hours^10^. Further, historical data tracking Epstein-Barr virus (EBV) DNA in the context of patients with nasopharyngeal cancer receiving radiation identified a rapid rise of ctDNA in the 24 h after radiotherapy initiation^11^. Additionally, acutely elevated ctDNA obtained via lymphatic exudate from surgical drains following head and neck carcinoma surgery was found to be positively correlated with risk of recurrence, independent of plasma ctDNA collected at later timepoints^12^. Cardiopulmonary exercise has even been suggested to acutely affect ctDNA levels in the minutes following exercise^13^. It has been widely theorized that there is a potential ‘surge’ or ‘transient peak’ of ctDNA immediately following therapy initiation due to induction of rapid cell death^14^. However, there is very little data regarding acute changes in ctDNA in the minutes to hours to days after initiation of systemic therapy and, in particular, whether different types of therapy (e.g. targeted therapy versus cytotoxic therapy) may result in distinct patterns.

To address this gap, in this study, we characterized ctDNA changes peri-infusion of targeted and cytotoxic therapies to address the hypothesis that initiation of systemic therapy would induce a short-term ‘surge’ in ctDNA. By studying samples from patients with metastatic triple-negative BC (mTNBC) enrolled in a phase 1b clinical trial testing paclitaxel and heat shock protein 90 (HSP90) inhibitor onalespib, we evaluated changes in ctDNA TF following targeted therapy alone, cytotoxic therapy alone, and the combination of both in the same patients with mTNBC, a cancer type associated with high levels of baseline ctDNA^1^.

## Results

### Patient Characteristics

**Fig. 1:**
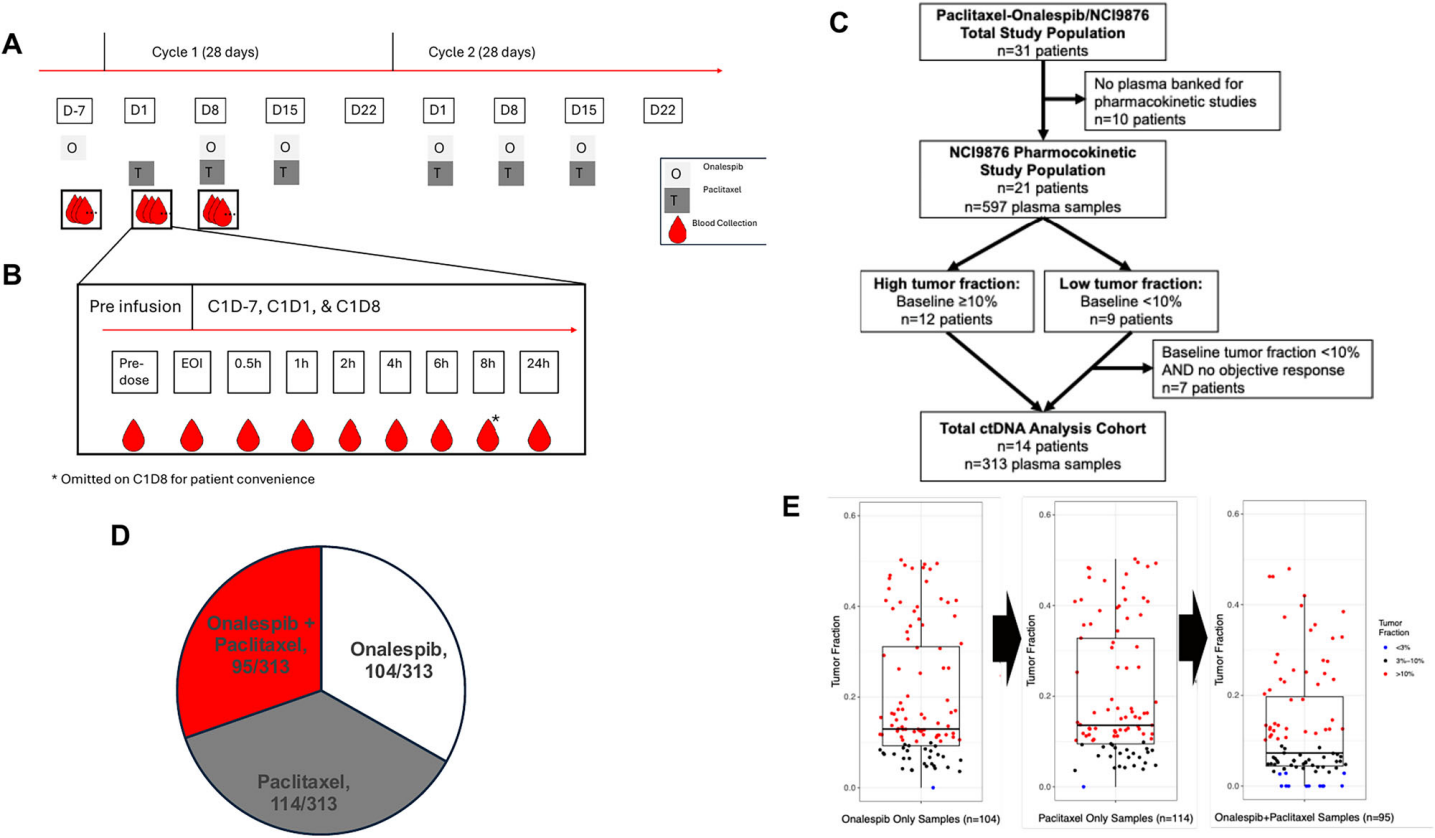
Study design and patient selection. **A** Dosing schedule with onalespib alone, paclitaxel alone, and combination onalespib and paclitaxel. Plasma was collected pre-infusion, end-of-infusion (EOI), and then 0.5/1/2/4/6/8/24 hours post-infusion on cycle 1 day-7, day 1, and day 8. **B** Distribution of plasma samples based on treatment. **C** CONSORT diagram outlining the selection criteria for patients included in the ctDNA analysis cohort. **D** Distribution of plasma sample based on treatment. **E** Box plot illustrating tumor fraction (TF) distribution by treatment group, categorized by TF < 3%, 3–10%, and >10%.

Thirty-one patients with advanced TNBC were enrolled in a phase 1b trial of HSP90 inhibitor onalespib in combination with paclitaxel (NCT02474173) (Fig. 1A, Table 1)^15^. To optimize detection of TF changes, shallow whole-genome sequencing (sWGS) was initially performed on one baseline plasma sample per patient followed by TF analysis with the ichorCNA algorithm. Patients with TF ≥ 10% and/or Response Evaluation Criteria in Solid Tumors (RECIST) objective response were selected for the final analysis cohort comprised of 14 patients (Fig. 1B). Patients were all Caucasian women with a median age of 53 years (range 29–74). Of the 14 patients, 8 had mTNBC at primary diagnosis (de novo disease), 4 were diagnosed with recurrent mTNBC, and 2 had metastatic hormone receptor-low (HR-low), human epidermal growth factor receptor 2 (HER2)-negative breast cancer (BC). All 14 patients had previously been treated with taxane therapy and had a median number of 1.5 prior lines of therapy. Four patients received onalespib dose level 1 (DL1; 120 mg/m^2^), 7 received DL3 (200 mg/m^2^), and 3 received DL4 (260 mg/m^2^).

**Table 1:** Patient Characteristics

Characteristic	Percentage
**Median Age**	53 (range 29–74)
**Sex**	
Female	100% (14/14)
**Race**	
White	100% (14/14)
**ECOG Performance Status**	
ECOG 0	50% (7/14)
ECOG 1	50% (7/14)
**Onalespib Dose Level (DL)**	
DL1	29% (4/14)
DL2	0% (0/14)
DL3	50% (7/14)
DL4	21% (3/14)
**ctDNA RECIST**	
Complete Response	21% (3/14)
Partial Response	14% (2/14)
Stable Disease	65% (9/14)
Progressive Disease	0% (0/14)
**Receptor Status**	
De Novo TNBC	57% (8/14)
Switched to TNBC	29% (4/14)
Weakly HR-positive	14% (2/14)
**Prior Treatment**	
Taxane Therapy	100% (14/14)
Median prior lines of therapy for metastatic disease	1.5 (range 0–8)
**Baseline Median TF**	15.87% (range 11.46–46.20%)

*ECOG* Eastern Cooperative Oncology Group, *ctDNA* circulating tumor DNA, *TNBC* triple-negative breast cancer, *HR* hormone receptor, *TF* tumor fraction.

All available samples (*n* = 313) from the 14 patients were subjected to sWGS. Of the 313 plasma samples, 104 (33.2%) were collected while patients were receiving onalespib alone, 114 (36.4%) while patients were on paclitaxel alone, and 95 (30.4%) while patients were on both onalespib + paclitaxel (Fig. 1C). The median baseline TF was 15.9%. Median TF for onalespib alone was 13.0%, for paclitaxel alone was 13.6%, and for combination onalespib + paclitaxel was 7.3% (Fig. 1D). Most samples had a TF > 3% based on cohort selection for high baseline TF.

### ctDNA Tumor Fraction Dynamics Pre- versus Post-Infusion of Targeted and Cytotoxic Therapies

**Fig. 2:**
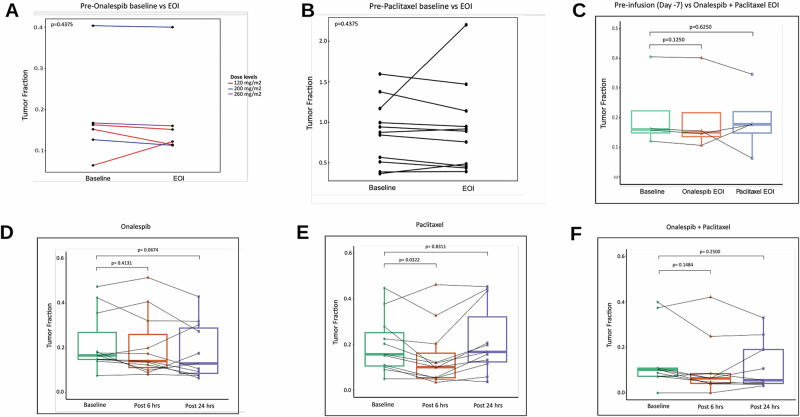
Significant decline in tumor fraction (TF) at 6 hours post-paclitaxel. **A** Change in TF from baseline to immediately post-infusion of onalespib at three dose levels: 120 mg/m^2^, 200 mg/m^2^, and 260 mg/m^2^. **B** Comparison of TF levels at baseline and immediately post-paclitaxel infusion. **C** Baseline TF vs. post-infusion of onalespib and paclitaxel. TF change from baseline compared to 6 and 24 h post-infusion of onalespib **D**, paclitaxel **E**, and combination onalespib and paclitaxel **F**.

To characterize early TF changes in response to treatment, we first compared TF pre-infusion against the end-of-infusion (EOI) timepoints to determine if there were rapid changes after infusion completion of onalespib alone, paclitaxel alone, or combination onalespib + paclitaxel. There was no significant change in TF at the EOI of onalespib compared to baseline regardless of DL (p = 0.44) (Fig. 2A). Similarly, TF at paclitaxel EOI did not significantly change compared to pre-infusion of paclitaxel (p = 0.4375) (Fig. 2B). When onalespib and paclitaxel were administered in combination on day 8, there was no significant change in TF at the onalespib EOI (p = 0.125) or paclitaxel EOI (p = 0.625) timepoints (Fig. 2C). This suggests that TF changes require more than the immediate peri-infusional time to change in response to treatment.

### ctDNA Tumor Fraction Dynamics Minutes to Hours Post-Infusion of Targeted and Cytotoxic Therapies

The a priori primary objective was to evaluate TF change at 6 hours and 24 hours post-infusion of onalepsib alone, paclitaxel alone, and the combination of both agents, based on historical data demonstrating a ‘surge’ at these timepoints post-radiation^11^. There was no significant change in TF at 6 hours (p = 0.41) or 24 hours (p = 0.067) post-infusion of onalespib alone (Fig. 2D). Interestingly, there was a significant decrease in TF at 6 hours post-infusion of paclitaxel (p = 0.03) (Fig. 2E). However, this decline in TF was not sustained at the 24-hour post-infusion timepoint (p = 0.83). There was no significant change in TF at 6 hours or 24 hours post-infusion of onalespib plus paclitaxel (all p > 0.05; Fig. 2F). As an exploratory analysis to investigate whether TF may demonstrate changes at timepoints other than the pre-specified 6 hours and 24 hours, we evaluated the baseline (pre-infusion) timepoint with each subsequent timepoint for onalespib alone (Supplementary Fig. 1A, left panel), paclitaxel alone (Supplementary Fig. 1A, middle panel), and the combination of onalespib + paclitaxel (Supplementary Fig. 1A, right panel). While there was no significant difference in TF in the hours after onalespib infusion alone, multiple timepoints after infusion of paclitaxel alone demonstrated a significant (nominal p < 0.05) decline, including 1 h, 2 h, 4 h, 6 h, and 8 h after infusion. For the combination of onalespib and paclitaxel infusion, there was a variable decline in TF but again no surge identified (Supplementary Fig. 1A). Overall, these data consistently demonstrated no surge of ctDNA in the minutes-hours post infusion of either targeted or cytotoxic therapy. Surprisingly, a decline in TF was detectable as soon as 1 hour after paclitaxel infusion. The notable TF declines at 1 h and 6 h timepoints following paclitaxel infusion suggests that the activity of paclitaxel is notably rapid and due to its cytotoxic nature. In comparison, onalespib dynamics as an HSP90 inhibitor may be more cytostatic in its anti-tumor effects, not affecting TF levels at early timepoints

### ctDNA Tumor Fraction Dynamics Over Weeks

**Fig. 3:**
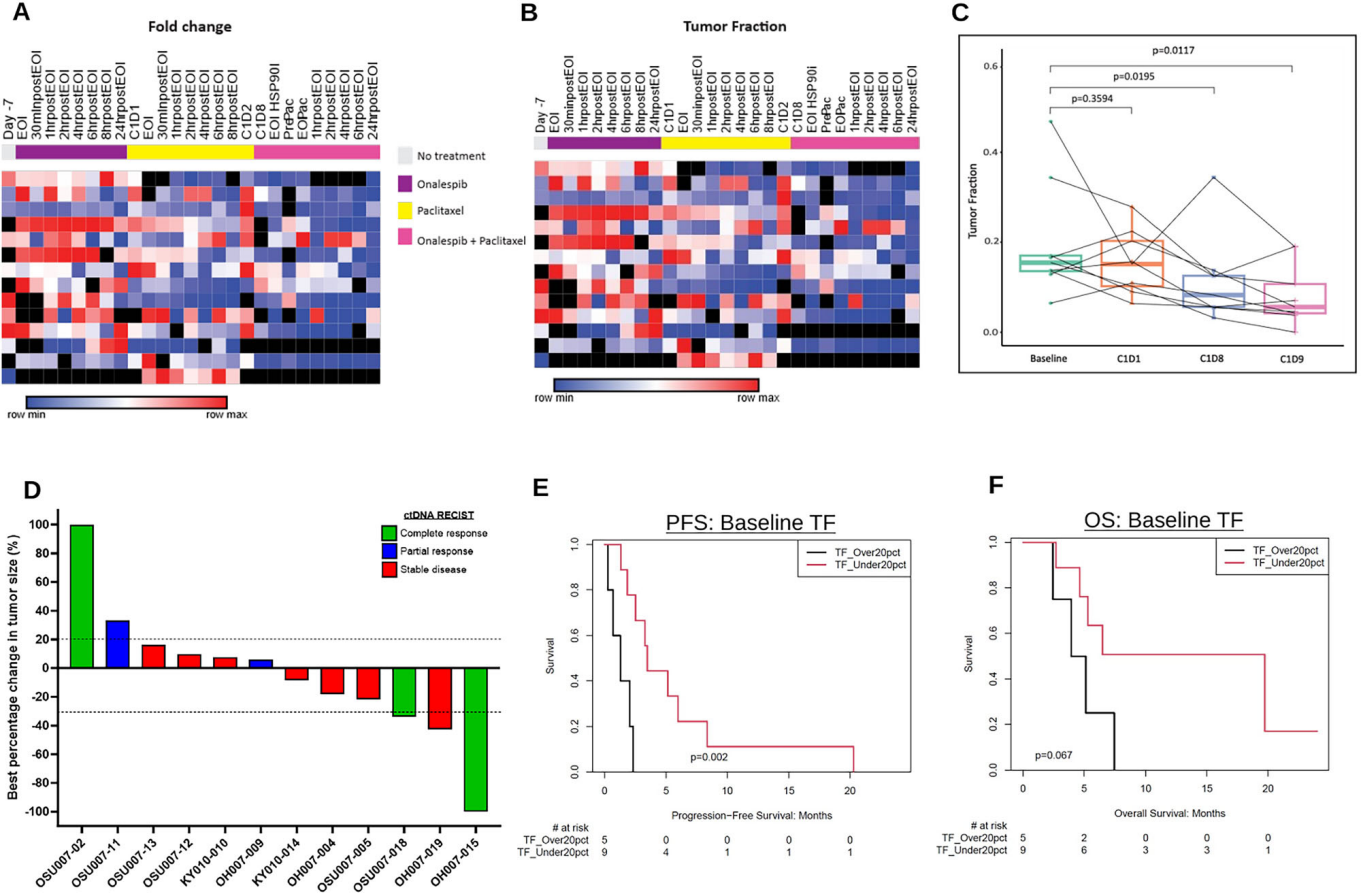
Baseline tumor fraction (TF) < 20% is associated with improved PFS. Heatmap depicting TF fold change **A** and TF levels **B** at available timepoints post-treatment during cycle 1 (C1). **C** Changes in TF at baseline compared to C1D1, C1D8, and C1D9. **D** Best percentage change in tumor size is presented with corresponding ctDNA-RECIST classification. Kaplan-Meier analysis of progression-free survival (PFS) **E** and overall survival (OS) **F** stratified by baseline TF greater or less than 20%.

When examining the global trend of ctDNA during cycle 1 of treatment, we observed an overall decline in ctDNA toward the end of the cycle when considering either fold change in TF (Fig. 3A) or raw TF value (Fig. 3B). To further confirm this finding, we compared overall baseline TF (collected prior to the day -7 onalespib infusion) to pre-infusion day 1, pre-infusion day 8, and 24 hours after onalespib + paclitaxel day 9. While there was no significant change on day 1 (*p* = 0.36), there was a significant reduction in TF observed at days 8 (*p* = 0.02) and 9 (*p* = 0.01) (Fig. 3C). Copy number analysis (CNA) via ichorCNA revealed a high degree of concordance post-treatment with onalespib or paclitaxel at corresponding timepoints (Supplementary Fig. 2). These results suggest that while acute TF reductions may be seen in the initial 6 h timepoint, sustained TF response requires at least several days’ worth of time following treatment.

### Correlation Between ctDNA RECIST and Size of Tumor

ctDNA-RECIST^16^ is a commonly used tool created to standardized measurements of ctDNA change and response. When we considered the lowest TF for each individual patient in cycle 1 (C1) to be the ‘best response’ using ctDNA-RECIST, 65% of patients were classified as stable disease, 21% as complete response, and 14% as partial response. When comparing ctDNA-RECIST response with changes in tumor size of the target lesion by imaging, we identified discordance between ctDNA-RECIST response and change in tumor size in half of the patients who had evaluable response (Fig. 3D). None of the patients were classified as progressive disease based on ctDNA-RECIST despite tumor growth. This finding highlights the limitations of our ctDNA measurement tools at this time.

### Tumor Fraction Change and Survival Outcomes

A decrease in ctDNA TF during treatment has been associated with better survival outcomes in advanced BC^4–9^. When comparing survival outcomes between patients who had baseline TF above vs. below 20%, patients with baseline TF below 20% were found to have an improved progression-free survival (PFS) rate (median PFS 3.5 months vs. 1.3 months in patients with TF ≥ 20%, *p* = 0.002) (Fig. 3E). There was a trend toward improved overall survival (OS) with a baseline below 20%; however, it was not statistically significant (median OS 19.7 months vs. 4.5 months in patients with TF ≥ 20%, p = 0.067) (Fig. 3F). When patients were stratified based on ctDNA-RECIST, however, there was no significant difference in PFS (*p* = 0.112) or OS (*p* = 0.131) (Supplementary Fig. S1B-C). Similarly, when comparing patients with a TF decrease greater vs. less than 50% from baseline to day 9, there was no significant difference in PFS (*p* = 0.289) or OS (*p* = 0.492) (Supplementary Fig. S1D-E). These results point towards absolute TF levels being the most predictive of survival outcomes in this study, as opposed to ctDNA-RECIST or measured change in ctDNA TF from baseline.

## Discussion

Despite the emerging role of ctDNA as a promising minimally invasive predictive biomarker, little is known about early changes in TF during and after systemic therapy initiation. In this study, over 300 banked plasma timepoints from a phase 1b clinical trial were used for serial ctDNA analysis during and after systemic therapy infusion^15^. A unique aspect of this cohort was that the same patients received the targeted therapy onalespib alone (day -7), the cytotoxic chemotherapy paclitaxel alone one week later (day 1), and both onalespib and paclitaxel together (day 8), which facilitated tracking dynamics of TF in response to distinct agents/agent types in a consistent population. Onalespib infusion did not elicit any significant change in TF immediately before the EOI or at 6 h or 24 h after, regardless of DL. On the other hand, after paclitaxel infusion, there was a decline in TF at multiple timepoints that was not sustained at 24 h post-infusion suggesting that paclitaxel – or chemotherapy in general – may be associated with earlier TF changes. This is the first study to our knowledge to demonstrate distinct ctDNA dynamics based on therapy type within the same patient population.

With growing evidence that serial monitoring of ctDNA may guide therapy decisions^17–19^, it is intriguing to consider how early changes may be detectable. With the knowledge that the ctDNA half-life is two hours or less, it is striking that, post-paclitaxel infusion, there was TF decline as soon as 1 h post-infusion that remained at multiple subsequent timepoints (2 h, 4 h, 6 h, 8 h); however, this was not sustained at 24 h post-infusion^12,20^. Previous studies have reported transient peaks in ctDNA after radiotherapy and chemotherapy initiation, possibly due to rapid cell death^20^. In studies conducted by two independent groups, a subset of head and neck cancer patients had an initial rise in ctDNA levels during the first week after radiation therapy^11,21^. Unexpectedly, we did not observe an upswing or surge in TF post treatment^19,20^. It is especially striking that there was no surge in TF in patients who had a complete response to treatment. A possible explanation for this discrepancy may be the differing mechanism by which cancer cell death is induced; for example, radiation therapy may cause a more simultaneous or widespread cancer cell death, leading to the release of a higher proportion of ctDNA at once, while systemic therapy may induce cell death over a more extended time period. Furthermore, differences in baseline ctDNA levels across different cancer types may influence the likelihood of detecting a transient peak after therapy^20^.

The data in this study supports existing/established findings regarding ctDNA in advanced (and specifically triple-negative) BC. Higher TF (greater than 20%) in our study population was associated with significant worsening of PFS and a trend toward lower OS. This finding is in line with prior studies showing an association between higher TF and worse prognosis^1,3^. There was also a significant decline in TF observed on days 8 and 9 during C1 of treatment, a result that is consistent with prior studies in advanced solid tumors that have demonstrated an improved PFS in patients with decreased ctDNA after treatment^7^. Notably, characterization of TF change using ctDNA-RECIST or based on TF decline from baseline to C1D9 was not associated with significant differences in PFS or OS. This finding suggests the need for further studies to identify the ideal parameters to evaluate TF change and its correlation with survival outcomes.

There are several limitations to this study. While over 300 plasma timepoints were evaluated, the overall number of patients was limited. Due to sample availability and quality, there were missing timepoints that further reduced the number of samples in certain analyses. In addition, the lack of racial/ethic diversity in this study (all patients were White) and single institutional location may hinder its generalizability.

In summary, this study demonstrates that ctDNA evaluation 1-2 weeks after starting treatment may be more insightful than sooner timepoints as TF changes may not be evident for days to weeks. Strikingly, there was no “surge” in ctDNA that corresponded to acute cell death even in patients with clinical complete response. By characterizing early ctDNA kinetics during pre- and post-infusional therapy, this study provides valuable insight toward the development of ctDNA as a therapeutic biomarker.

## Methods

### Patient Selection and Study Design

Eligible patients were consented to and enrolled in a National Cancer Institute (NCI)-sponsored phase 1b clinical trial at four different academic medical centers and evaluated safety, recommended phase 2 dose and preliminary efficacy of the combination of onalespib, a small molecule inhibitor of HSP90, and paclitaxel in advanced TNBC (ClinicalTrials.gov ID: NCT02474173; date of registration was June 17^th^, 2015)^15^. A CONSORT 2025 checklist for this trial can be found in Supplementary Table 1. Patients with histologically confirmed advanced TNBC or HR-low BC who were 18 years of age or older were considered eligible for this study. TNBC was defined as <1% expression of estrogen receptor (ER) and progesterone receptor (PR), and HR-low was defined as <10% ER and PR. For both TNBC and HR-low BC, patients enrolled had no overexpression of HER2 (0 or 1 + ) based on immunohistochemistry (IHC) staining or 2 + IHC with negative HER2 fluorescent in-situ hybridization (FISH). Patients with prior exposure to any number of chemotherapy and endocrine treatments for metastatic disease were considered eligible. Paclitaxel and onalespib were given as intravenous infusions, each one over 60 minutes. Onalespib was administered as a single agent on day -7 and paclitaxel was administered as a single agent on C1, day 1 (C1D1). Both onalespib and paclitaxel were administered together on days 8 and 15 of a 28-day cycle. After C1, onalespib and paclitaxel were administered together on days 1, 8, and 15 of 28-day cycles (Fig. 1A). The starting dose of onalespib was 120 mg/m^2^ (DL1) and escalated to DL2-4 to a maximum of 260 mg/m^2^ based on toxicity, using standard 3 + 3 dose escalation study design. Paclitaxel was administered at the standard dose of 80 mg/m^2^. The full protocol, including a detailed study design and eligibility criteria, is available in Supplementary Fig. 3.

### Ethics

The study protocol was approved by the Institutional Review Board (IRB) at each of the participating institutions as well as the NCI Central IRB under common study number NCI9876. This study protocol followed the Declaration of Helsinki and International Conference on Harmonization Good Clinical Practice (ICH-GCP) guidelines. Written informed consent was obtained from all patients. The study was sponsored by the NCI and registered at ClinicalTrials.gov (NCT02474173).

### Sample Collection

Blood samples were collected in EDTA tubes and processed to components within 1 h of draw, from which plasma was isolated and stored at −80 °C for pharmacokinetic analysis. Blood was collected at multiple timepoints on days -7, 1, and 8. Samples were obtained on days -7 and 1 of C1 at pre-infusion, immediately before the EOI, and the following timepoints post-infusion: 0.5, 1, 2, 4, 6, 8, and 24 h (Fig. 1A). On C1, day 8, samples were obtained pre-infusion, immediately before the EOI, and the following timepoints post-infusion: 0.5, 1, 2, 4, 6, 8, and 24 h.

### Circulating Tumor DNA Extraction and Sequencing

Banked frozen aliquots of plasma were thawed at room temperature and centrifuged at 15,000 × *g* for 10 min at room temperature. To remove residual cells from the plasma, samples were centrifuged in low-bind tubes. The Qiagen Circulating DNA kit on the QIAsymphony liquid handling system was used to extract circulating free DNA (cfDNA) from 1 to 7 ml of plasma, which was eluted into 40–80 μL of resuspension buffer. Extracted ctDNA was quantified on the PicoGreen (Life Technologies) assay on a Hamilton STAR-line liquid handling system. The Kapa HyperPrep kit (IDT, Coralville, IA) with IDT’s duplex unique molecular identifier (UMI) adapters was used for library construction of cfDNA. For enzymatic cleanups, Beckman Coultier AMPure XP beads were used. The ctDNA library was quantified using the Invitrogen Quant-It broad-range dsDNA quantification assay kit (Thermo Scientific Catalog Q33130). The prepared libraries were pooled and sequenced on paired 151 base pair runs on HiSeq X (Illumina) to an average genome-wide fold coverage of ~0.3×. TF was estimated from sWGS using ichorCNA. ctDNA-RECIST was used to evaluate ctDNA response^16^. This method has undergone extensive prior analytical validation including evaluation of assay sensitivity, precision, repeatability, reproducibility, pre-analytic factors, and DNA quality/quantity^22^.

### Statistical Tests and Data Visualization

All plots were generated using R 4.4.2. Heatmaps were generated with the Broad Institute Morpheus online tool. Statistical analysis was performed with the Wilcoxon matched paired sign-rank test on Graphpad Prism. Statistical significance was defined as a two-sided alpha of 0.05 or less.

## Supplementary information

02.09.2026 Supp Material

## Data Availability

The human sequence data generated in this study are not publicly available due to patient privacy requirements but are available upon reasonable request from the corresponding author.

## Code availability

Not applicable to this project.
