# Supplementary material for: Acute circulating tumor DNA dynamics during and after systemic therapy initiation for advanced triple-negative breast cancer
Source: NPJ Breast Cancer. 2026 Apr 16;12:82. doi: 10.1038/s41523-026-00953-w (PMC13260934; doi:10.1038/s41523-026-00953-w)
Supplement: Supplementary file 1 — 02.09.2026 Supp Material [file 41523_2026_953_MOESM1_ESM.pdf]

Supplementary Figure 1

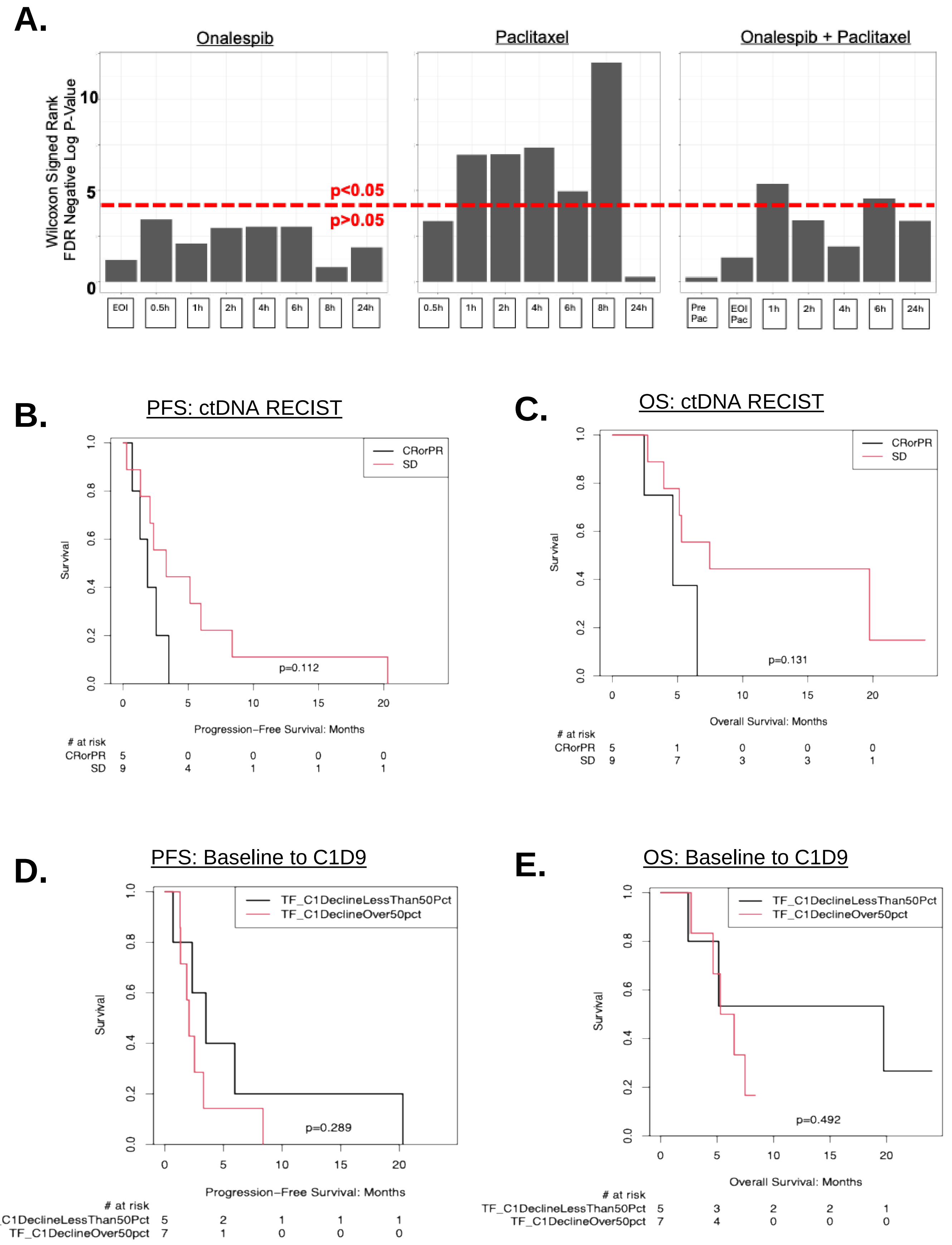

Supplementary Figure 1. No association between progression-free survival (PFS) and overall survival (OS) with ctDNA-RECIST. (**A**)The Wilcoxon signed-rank test, with false discovery rate (FDR) correction, was used to compare baseline tumor fraction (TF) and post-treatment TF. The resulting p-values are depicted in the bar graph as negative log-transformed values. Kaplan-Meier curve showing PFS **(B)** and OS (**C**) based on ctDNA-RECIST stratification. Kaplan-Meier analysis comparing PFS (**D**) and OS (**E**) between patients with a greater than 50% decline versus less than 50% decline in TF at C1D9.

Supplementary Figure 2

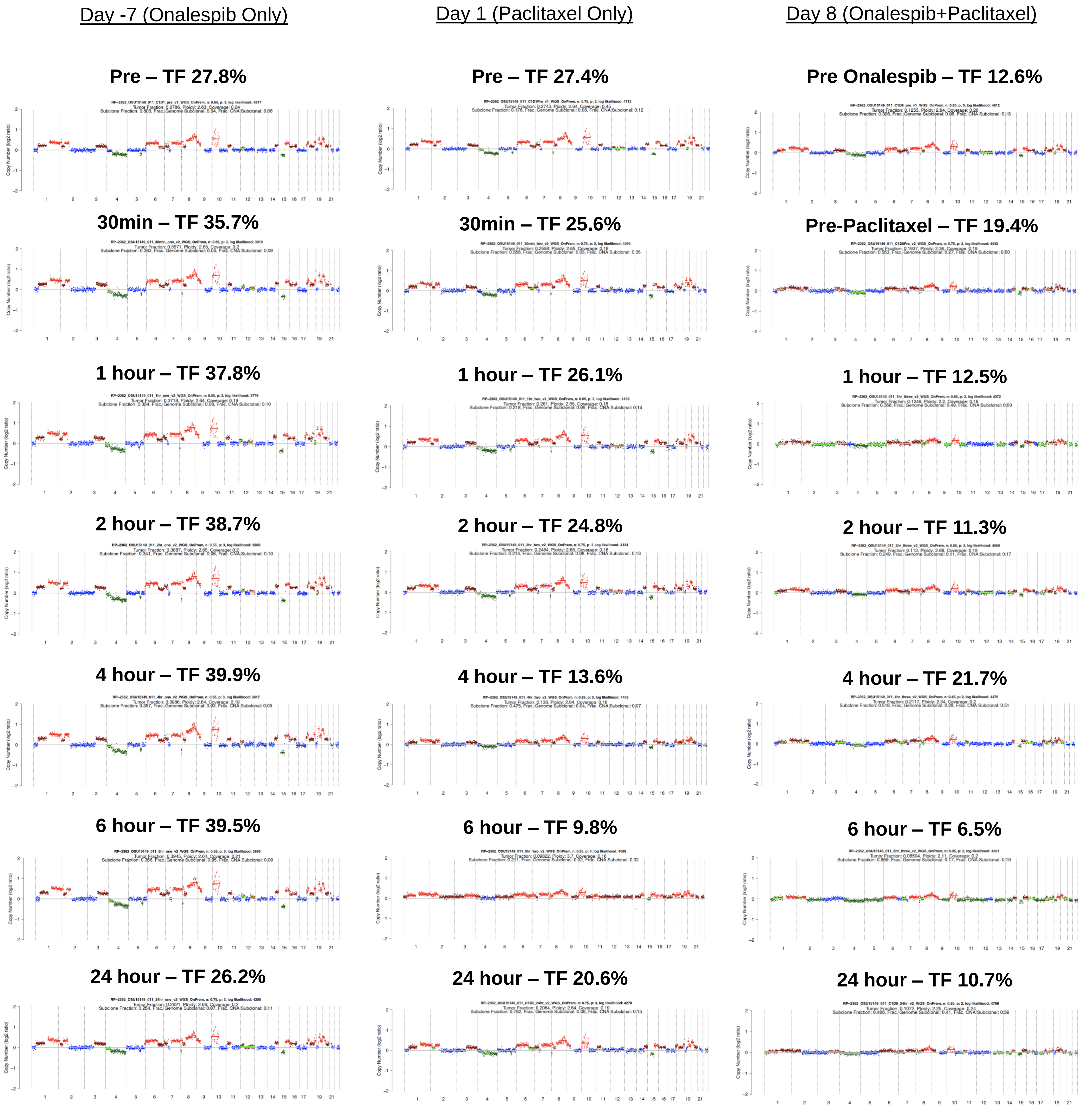

Supplementary Figure 2. Copy number alterations profiles. Representative copy number plots from plasma collected at various timepoints post-treatment for one patient.

Supplementary Figure 3: Clinical trial protocol from Phase Ib Study of HSP90 Inhibitor, AT13387 (onalespib) in Combination with Paclitaxel in Patients with Advanced, Triple Negative Breast Cancer (ClinicalTrials.gov ID: NCT02474173).

NCI Protocol #: 9876

Local Protocol #: OSU 15149

ClinicalTrials.gov Identifier: NCT02474173

TITLE: Phase 1b Study of HSP90 Inhibitor, AT13387 (onalespib) in Combination with Paclitaxel in Patients with Advanced, Triple Negative Breast Cancer.

Corresponding Organization: LAO-OH007 / Ohio State University Comprehensive Cancer Center LAO

Principal Investigator: Robert Wesolowski, MD
Division of Medical Oncology
B401 Starling Loving Hall
320 W. 10th Ave.
Columbus, OH 43210
(614) 293-9273
(614) 293-4372
Email: Robert.wesolowski@osumc.edu

Senior Co-Investigator: Bhuvaneswari Ramaswamy, MD
B406 Starling Loving Hall
320 W. 10th Ave.
Columbus, Ohio 43210
Tel: (614) 293-6401
Fax: (614) 293-7264
Bhuvaneswari.Ramaswamy@osumc.edu

Participating Organizations:

LAO-MD017 / JHU Sidney Kimmel Comprehensive Cancer Center LAO
LAO-OH007 / Ohio State University Comprehensive Cancer Center LAO
LAO-PA015 / University of Pittsburgh Cancer Institute LAO

Non-Member Collaborators*:* None

Statistician:
Julie Stephens, MS
Center for Biostatistics
Ohio State University
1800 Cannon Drive
Columbus OH 43210
Email: Julie.Stephens@osumc.edu
Tel: 614-293-9645
Fax: 614-293-0049

Study Coordinator:
Megan Laibach-Thompson
Comprehensive Cancer Center
Ohio State University
1145 Olentangy River Road, Rm 2054a
Columbus, OH 43212
Email: Megan.LaibachThompson@osumc.edu
Tel: 614-293-0069
Fax: 614-293-0049

Responsible Research Nurse:
Megan Laibach-Thompson
Comprehensive Cancer Center
Ohio State University
1145 Olentangy River Road, Rm 2054a
Columbus, OH 43212
Email: Megan.LaibachThompson@osumc.edu
Tel: 614-293-0069
Fax: 614-293-0049

Multi-Center Trial Coordinator:
OSU Multi-Site Team
Comprehensive Cancer Center
Ohio State University
600 Ackerman Rd., Rm 1050, Cube175
Email: OSUCCC-CTO-MCTP@osumc.edu
Tel: 614-293-3657
Fax: 614-366-6652

NCI-Supplied Agent: AT 13387 (NSC #749712)
Other Agent(s): Paclitaxel (NSC# 125973, Supplier: Commercial)

IND #: 127423
IND Sponsor: DCTD, NCI

Protocol Type:
Original / Version #1.0 / Version Date: 05/04/2015
Revision 1 / Version #1.1 / Version Date: 07/09/2015
Revision 1 / Version #1.2 / Version Date: 08/14/2015
Revision 2 / Version #2.0 / Version Date: 09/03/2015
Revision 3 / Version #3.0 / Version Date: 10/15/2015
Revision 4 / Version #4.0 / Version Date: 12/02/2015
Revision 5 / Version #5.0 / Version Date: 12/18/2015
Amendment 1 / Version #1 / Version Date: 06/10/2016
Amendment 2 / Version #1 / Version Date: 08/30/2016
Amendment 3 / Version #1 / Version Date: 09/30/2016
Amendment 4 / Version #1 / Version Date: 10/27/2016
Amendment 5 / Version #3 / Version Date: 11/21/2016

Amendment 6 / Version #1 / Version Date: 03/06/2017
Amendment 7 / Version #1 / Version Date: 05/23/2017
Amendment 8 / Version #1 / Version Date: 06/26/2017
Amendment 9 / Version #1 / Version Date: 07/12/2017
Amendment 10 / Version #1 / Version Date: 08/04/2017
Amendment 11 / Version #1 / Version Date: 01/17/2018
Amendment 12 / Version #1 / Version Date: 03/19/2018
Amendment 13 / Version #1 / Version Date: 05/07/2018
Amendment 14 / Version #1 / Version Date: 08/22/2018
Amendment 15 / Version #1 / Version Date: 01/22/2019
Amendment 16 / Version #1 / Version Date: 03/07/2019
Amendment 17 / Version #1 / Version Date: 05/03/2019
Amendment 18/ Version #1 / Version Date: 06/26/2019
Amendment 19 / Version #1 / Version Date: 08/21/2019
Amendment 20 / Version #1 / Version Date: 07/31/2020

STUDY SYNOPSIS:

STUDY OBJECTIVES:

Primary Objectives:

- To determine the recommended phase 2 dose (RP2D) of AT13387 in combination with paclitaxel in patients with advanced triple negative breast cancer (TNBC).
- To determine the toxicity profile (based on CTCAE v. 5.0) of AT13387 in combination with paclitaxel in patients with advanced TNBC.

Secondary Objectives:

- To determine the effect of AT13387 on pharmacokinetics of paclitaxel in the study population
- To determine the effect of paclitaxel on pharmacokinetics of AT13387 in the study population
- To determine the overall response rate (partial response + complete response), response duration and progression-free survival in the study patients

SUMMARY OF STUDY DESIGN:

This is a phase 1b trial in women or men with unresectable, locally advanced or metastatic triple negative breast cancer who received any number of prior lines of chemotherapy. The study will utilize 3+3 design and will test the combination of HSP90 inhibitor AT13387 and Paclitaxel given on days 1, 8, 15 on 28 day cycles.

In order to establish effects of paclitaxel on pharmacokinetics of AT13387, AT13387 will be given as a single agent on day -7 (+/- 3 days) prior to Cycle 1, Day 1 of treatment. During Cycle 1, Day 1, patients will receive paclitaxel alone. Both agents will then be administered on days 8 and 15 of a 28 day cycle for the remainder of cycle 1. Following cycle 1, all patients will receive treatment with AT13387 combined with paclitaxel on days 1, 8, and 15 of 28 day cycles.

This design will allow establishment of the following:

1) The effect of paclitaxel on PK of AT13387 by measuring PK of AT13387 during Day -7 (+/- 3 days) and Cycle 1, Day 8.
2) The effect of AT13387 on PK of paclitaxel by measuring PKs of paclitaxel during Cycle 1, Day 1 and Cycle 1, Day 8.

Please see Figure 1 for the diagram summary of the dose escalation part.

Astex Pharmaceuticals has discontinued the production of Onalespib (AT13387). Patients currently receiving onalespib, including those enrolled before December 1, 2019, will continue to receive the drug based on the current supply forecast. Drug availability should be confirmed with CTEP for any patient who is considering enrollment on/after September 1st, 2019. Investigators of all open ("Active") trials are asked to change the protocol status to "Closed to Accrual" by December 1, 2019. All patients must be off-treatment by May 31, 2020.

FIGURE 1: STUDY SCHEMA

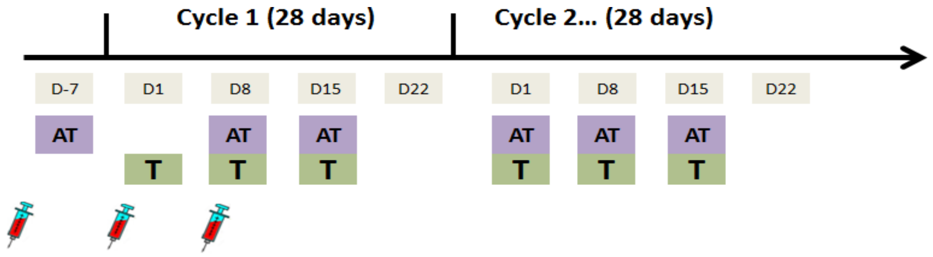

- AT 13387 will be given as a single agent on days -7 (+/- 3 days)
- Paclitaxel will be given as a single agent on day 1 of cycle 1.
- Paclitaxel and AT 13387 will be given on days 8, 15 on 28 day cycle (during cycle 1)
- Paclitaxel and AT 13387 will be given on days 1, 8, 15 on 28 day cycle starting with cycle 2.

D# Day of treatment and/or assessment

AT AT 13387 at escalating doses

Peripheral blood collection for pharmacokinetics

T Paclitaxel at 80 mg/m2 IV

Since adequate data to indicate no major drug-drug interactions have already been collected, pharmacokinetics of paclitaxel and AT13387 became optional starting with protocol version 17 dated 05/03/2019. Patients who opt out from pharmacokinetic studies will not need to receive day -7 of AT13387 and can start both AT13387 and paclitaxel on day 1 of cycle 1.

STUDY TREATMENT:
The doses of AT13387 will be gradually escalated while the doses of paclitaxel will be kept at 80 mg/m^2 throughout all dose levels.

Dose Escalation Schedule		
**Dose Level[b,c]**	**Dose[a]**	
	AT13387 (mg/m^2 IV on days 1, 8, 15)	***Paclitaxel (mg/m^2 IV on days 1, 8, 15)[d]***
Level -1	100	80
Level 1[e]	120	80
Level 2	150	80
Level 3	200	80
Level 4	260	80

[a]Doses are stated as exact dose in mg/m^2
[b]Cycle length will be 28 days (except for safety run-in part when single agent AT 13387 will be given on Day -7 +/- 3 days).
[c]Dose modifications or interruptions of AT13387 and Paclitaxel will not be allowed during the DLT period (cycle 1 of therapy) unless a patient experiences 1 or more dose limiting toxicities; or if the patient develops toxicities that require dose interruption based on dose modification tables in section 6, in which case either or both of the study medications may be held. If either study medication is held, all missed doses will not be made up upon resuming study therapy.
[d]In order to establish effects of AT13387 on pharmacokinetics of paclitaxel, paclitaxel will be given as a single agent during Cycle 1, Day 1.
[e]Starting Dose

ASSESSMENT OF DOSE LIMITING TOXICITIES:
Subjects who complete cycle 1 of therapy or those who have received at least 2 of the 3 doses of taxol and AT13387 in cycle 1 will comprise the DLT population, and will help determine the recommended phase 2 dose (RP2D). Dose modifications or interruptions of AT13387 and Paclitaxel will not be allowed during the DLT period unless a patient experiences 1 or more dose limiting toxicities; or if the patient develops toxicities that require dose interruption based on dose modification tables in section 6, in which case either or both of the study medications may be held. If either study medication is held, all missed doses will not be made up upon resuming study therapy. Patients who do not complete the DLT period for reasons other than toxicities will be replaced. However, patients who develop toxicities that are at least possibly related to study therapy will not be replaced and will count towards DLT determination, even if they do not complete all treatments in cycle 1. Grading of toxicities will be assessed by the use of Common TerminologyCriteria for Adverse Events (CTCAE) version 5.0. DLTs will consist of any adverse events that are at least possibly attributed to AT13387 and are further defined as follows:

Hematologic DLTs

- Grade 4 neutropenia lasting for ≥7 days in duration
- Grade ≥3 neutropenia complicated by a fever
- Grade 4 thrombocytopenia
- Grade 3 thrombocytopenia complicated by bleeding

Non-hematologic DLTs

- Any CTCAEv5.0 Grade ≥3 non-hematologic toxicity, unless the event is clearly unrelated to treatment EXCEPT thefollowing:

Grade ≥3 nausea, vomiting, or diarrhea that resolves to Grade ≤2 within 48 hours, with or without medical intervention or prophylaxis

- Grade 3 fatigue that resolves to Grade ≤2 within 14 days

Transient (<14 days) increase in LFTs (of ≤ one Grade in severity) compared to baseline levels in patients with baseline liver metastases.

- Grade 3 maculopapular rash for which symptoms are easily managed with supportive care and no evidence of superinfection or limitation of self-care ADLs.

If, during a DLT period, a patient experiences a toxicity that does not clearly fit any of the above DLT criteria but which in the opinion of the investigator is highly clinically significant, the toxicity may be considered a DLT after a discussion with the Principal Investigator.

The following dose escalation procedures will be followed:

Number of Patients with DLT at a Given Dose Level	Escalation Decision Rule
0 out of 3	Enter 3 patients at the next dose level.
≥2	Dose escalation will be stopped. This dose level will be declared the maximally administered dose (highest dose administered). Three (3) additional patients will be entered at the next lowest dose level if only 3 patients were treated previously at that dose.
1 out of 3	Enter at least 3 more patients at this dose level.<br>• If 0 of these 3 patients experience DLT, proceed to the next dose level.<br>• If 1 or more of this group suffer DLT, then dose escalation is stopped, and this dose is declared the maximally administered dose. Three (3) additional patients will be entered at the next lowest dose level if only 3 patients were treated previously at that dose.
≤1 out of 6 at highest dose level below the maximally administered dose	This dose will become maximum tolerated dose. At least 6 patients must be entered at the recommended phase 2 dose.

Once maximum tolerated dose is established, an additional 9 patients will be enrolled to that dose (for a total of 15 patients). This will allow the following: (1) further characterize the safety and tolerability of the combination; (2) provide additional data on effect of either agent on PK of the other agent and; (3) provide better estimates for anti-tumor activity with more precision levels. The toxicity monitoring guidelines for these additional 9 patients will be the same as for theother study patients. The DLT rate of ≥30 percent will be deemed unacceptable. Therefore, if 5 or more of 15 patients develop a DLT, the protocol will be amended to consider dose expansion part in the dose level below previous maximum tolerated dose. However, if <5 of 15 patients develop the DLT, the dose will be declared as the recommended phase 2 dose.

The R2PD will be defined as: (1) MTD, i.e. the highest doses of the combination of AT13387 and paclitaxel at which no more than one of 6 patients experiences a DLT or (2) doses of the combination below MTD, if in the opinion of the investigators, lower doses are better tolerated and safer. The first criterion will be given the priority when determining RP2D. The second criterion will only apply if an unexpected and highly clinically significant toxicity is noted that does not neatly fit the DLT criteria (for example, >1 patient in a given dose level develops grade 4 thrombocytopenia after cycle 2).

INCLUSION CRITERIA (ABBREVIATED):

- Patients must have histologically confirmed measurable or non-measurable advanced or metastatic breast cancer for which standard curative measures do not exist or are no longer effective.

- Primary and/or metastatic breast tumor must be negative for over-expression of estrogen and progesterone receptors. Patients with weak estrogen receptor and/or progesterone receptor expression (<10% on IHC) will be eligible.

- Primary and/or metastatic breast tumor must be negative for HER-2/neu over-expression based on immunohistochemistry (IHC) (0 or 1+, 2+ if Fluorescence In-Situ Hybridization (FISH) test is negative) or FISH (HER2/CEP17 ratio <2.0 or <4 Her-2/neu signals per nucleus).

- Any number of prior therapies for metastatic breast cancer is allowed. Patients with weakly estrogen receptor positive breast cancer who received any number of endocrine agents for metastatic breast cancer will also be eligible.

- Prior taxane is allowed (as long as the patient is not experiencing grade >1 neuropathy and had no history of disease progression on a taxane therapy within 3 months prior to study enrollment).

- Age ≥18 years.

- ECOG performance status ≤2 (Karnofsky ≥60%, see APPENDIX A: PERFORMANCE STATUS CRITERIA).

- Life expectancy of greater than 12 weeks

- Patients must have normal organ and marrow function as defined below:

– leukocytes	≥2,000/µL
– absolute neutrophil count	≥1,500/µL
– platelets	≥100,000/µL
– total bilirubin	less than or equal to the institution's upper limit of normal
– AST(SGOT)/ALT(SGPT)	≤2.5 × institutional upper limit of normal (except for patients with liver metastases in whom AST/ALT can be <5 x institutional upper limit ofnormal)
– Creatinine within normal institutional limits OR creatinine clearance	≥50 mL/min for patients with creatinine levels above institutional normal
– Left ventricular ejection fraction of	>50% on baseline echocardiography or multi-gated acquisition (MUGA) scan
– QTc of	<480 milliseconds

- Female subjects with child bearing potential must have a negative pregnancy test at screening.

- Women of child-bearing potential and men must agree to use adequate contraception prior

to study entry, for the duration of study participation and for 3 months after completion of study treatment administration. Adequate contraception includes methods such as oral contraceptives, double barrier method (condom plus spermicide or diaphragm), or abstaining from sexual intercourse.

- Ability to understand and the willingness to sign a written informed consent document.

EXCLUSION CRITERIA (ABBREVIATED):

- Patients who have had chemotherapy within 4 weeks (6 weeks for nitrosoureas or mitomycin C) prior to entering the study.

- Patients who are receiving any other investigational agents within 4 weeks or 5 half-lives (whichever is shorter) prior to the first dose of the study regimen.

- Prior radiation therapy within 2 weeks prior to the first dose of the study regimen.

- Patients in whom prior treatment related toxicities have not recovered to grade 1 or less (except for alopecia).

- Recent initiation of bone modifying therapy with a bisphosphonate or denosumab unless it has been started more than 4 weeks prior to the first dose of the study regimen. Patients who are already enrolled in this study can initiate bone modifying therapy after the first set of re-staging scans (≥8 weeks from cycle 1, day 1).

- Prior therapy with AT13387 or another HSP90 inhibitor.

- Patients with known brain metastases. However, patients with previously treated and stable brain metastases are eligible as long as they are no longer requiring steroids, completed radiation therapy more than 2 weeks prior to the first dose of study regimen and have no seizures or worsening neurologic symptoms.

- History of grade 3-4 immediate hypersensitivity reaction to paclitaxel.

- History of clinically significant allergic reactions attributed to compounds of similar chemical or biologic composition to AT13387 or paclitaxel.

- Uncontrolled intercurrent illness including, but not limited to, ongoing or active infection, symptomatic congestive heart failure, unstable angina pectoris, cardiac arrhythmia, or psychiatric illness/social situations that would limit compliance with study requirements.

- Pregnant women. Because there is an unknown but potential risk for adverse events in nursing infants secondary to treatment of the mother with AT13387 and paclitaxel,

breastfeeding should be discontinued if the mother is treated with AT13387 and paclitaxel.

- Patients who are HIV positive on highly active anti-retroviral therapy (HAART) will be excluded from the study because of the potential for pharmacokinetic interactions with AT13387.

- Inability to understand and sign informed consent.

- Any other medical or psychiatric condition that in the opinion of the investigator would make the study therapy unsafe for thepatient.

CORRELATIVE STUDIES:
The focus of this phase I trial will be assessment of safety, tolerability and determination of a recommended phase 2 dose of the study regimen. Given that this trial will enroll a small number of patients, extensive correlative studies will be deferred to a future phase 2 clinical trial. However, a limited number of correlatives performed that are outlined below.

a. Pharmacokinetics:
The effect of AT13387 on pharmacokinetics of paclitaxel and the effect of paclitaxel on pharmacokinetics of AT13387 will be evaluated to ensure that these agents do not have clinically significant interactions. Blood samples for pharmacokinetics will be collected on Day -7 (+/- 3 days), Cycle 1, Day 1 and Cycle 1, Day 8. Since adequate data to indicate no major drug-drug interactions have already been collected, pharmacokinetics of paclitaxel and AT13387 became optional starting with protocol version 17 dated 05/03/2019.

NUMBER OF PATIENTS:
Depending on the number of dose limiting toxicities and recommended phase 2 dose, approximately 6 to 33 patients will be enrolled in this study.

TABLE OF CONTENTS

STUDY SYNOPSIS 4
FIGURE 1: STUDY SCHEMA 5
1. OBJECTIVES 14
1.1 Primary Objectives 14
1.2 Secondary Objectives 14
2. BACKGROUND 14
2.1 Study Disease 14
2.2 CTEP IND Agent 17
2.3 Other Agent 27
2.4 Rationale 28
2.5 Correlative Studies Background 29
3. PATIENT SELECTION 29
3.1 Eligibility Criteria 29
3.2 Exclusion Criteria 31
3.3 Inclusion of Women and Minorities 32
4. REGISTRATION PROCEDURES 33
4.1 Investigator and Research Associate Registration with CTEP 33
4.2 Site Registration 34
4.3 Patient Registration 36
4.4 General Guidelines 38
5. TREATMENT PLAN 38
5.1 Screening Procedures 38
5.2 Agent Administration 39
5.3 Definition of Dose-Limiting Toxicity 43
5.4 General Concomitant Medication and Supportive Care Guidelines 46
5.5 Duration of Therapy 46
5.6 Duration of Follow Up 46
5.7 Criteria for Removal from Study 47
6. DOSING DELAYS/DOSE MODIFICATIONS 47
6.1 Dose Modifications of AT13387 47
6.2 Dose Modifications of Paclitaxel 48
6.3 Guidelines for dose modifications of AT13387 and Paclitaxel for toxicities 49
7. ADVERSE EVENTS: LIST AND REPORTING REQUIREMENTS 52
7.1 Comprehensive Adverse Events and Potential Risks List(s) (CAEPRs) 52
Comprehensive Adverse Events and Potential Risks list (CAEPR) for AT13387 (Onalespib, NSC 749712) 52
7.2 Adverse Event Characteristics 59
7.3 Expedited Adverse Event Reporting 60
7.4 Routine Adverse Event Reporting 62
7.5 Secondary Malignancy 62
7.6 Second Malignancy 62

8. PHARMACEUTICAL INFORMATION ... 62
8.1 CTEP IND Agent ... 62
Availability ... 64
8.2 Other Investigational Agent(s) ... 66
8.3 Commercial Agent(s) ... 66
9. BIOMARKER, CORRELATIVE, AND SPECIAL STUDIES ... 67
9.1 Integral Laboratory or Imaging Studies ... 68
9.2 Integrated Correlative Studies ... 68
9.3 Exploratory/Ancillary Correlative Studies ... 71
9.4 Special Studies ... 71
10. STUDY CALENDAR ... 72
11 MEASUREMENT OF EFFECT ... 74
11.1 Antitumor Effect – Solid Tumors ... 74
For Patients with Measurable Disease (*i.e.*, Target Disease) ... 79
11.2 Antitumor Effect – Hematologic Tumors ... 80
11.3 Other Response Parameters ... 80
12. STUDY OVERSIGHT AND DATA REPORTING / REGULATORY REQUIREMENTS ... 80
12.1 Study Oversight ... 80
12.2 Data Reporting ... 81
12.3 Data and Safety Monitoring ... 83
12.4 Collaborative Agreements Language ... 83
13. STATISTICAL CONSIDERATIONS ... 85
13.1 Study Design/Endpoints ... 85
13.2 Sample Size/Accrual Rate ... 86
13.3 Stratification Factors ... 87
13.4 Analysis of Secondary Endpoints ... 87
14. REFERENCES ... 89

APPENDIX A: PERFORMANCE STATUS CRITERIA ... 93
APPENDIX B: INFORMATION ON POSSIBLE DRUG INTERACTIONS ... 94
APPENDIX C: PHARMACOKINETIC (PK) SAMPLES REQUISITION FORM ... 96
APPENDIX D: LIST OF CLINICALLY SIGNIFICANT OR STRONG CYP 3A4 AND CYP2C8 INHIBITORS AND INDUCERS ... 97

1 OBJECTIVES

1.1 Primary Objectives

1.1.1 To determine the recommended phase 2 dose (RP2D) of AT13387 in combination with paclitaxel in patients with advanced triple negative breast cancer (TNBC).

1.1.2 To determine the toxicity profile (based on CTCAE v. 5.0) of the combination of AT13387 in combination with paclitaxel in patients with advanced TNBC.

1.2 Secondary Objectives

1.2.1 To determine the effect of AT13387 on pharmacokinetics of paclitaxel in the study patient population.

1.2.2 To determine the effect of paclitaxel on pharmacokinetics of AT13387 in the study patient population.

1.2.3 To observe anti-tumor activity by determining the overall response rate (partial response + complete response), response duration and progression-free survival. Although the clinical benefit of the combination of AT13387 and Paclitaxel has not yet been established, the intent of offering this treatment is to provide a possible therapeutic benefit, and thus the patients will be carefully monitored for tumor response and symptom relief in addition to safety and tolerability.

2. BACKGROUND

2.1 Study Disease

Triple negative breast cancer (TNBC) represents about 15-20% of approximately 240,000 new breast cancer cases diagnosed in the United States annually. Compared to other types of breast cancer, TNBC is associated with increased likelihood of early local and distant recurrence, poorer prognosis and lack of molecular based treatment options. There are currently no curative therapy options for patients with stage IV TNBC who have a median survival of approximately 1-2 years (compared to patients diagnosed with hormone receptor positive or HER-2/neu amplified breast cancer whose median survival approaches 3 or more years) [1, 2]. Unlike hormone receptor positive or HER-2/neu over-expressing breast cancer, there are no approved targeted therapies for patients diagnosed with TNBC and conventional chemotherapy remains the mainstay of treatment. This is likely one of the reasons why there has been little improvement in survival of patients with TNBC over the last several decades. Better, more effective therapeutic options for patients with advanced TNBC that would lead to clinically significant advances in patient outcomes are highly needed and clearly represent an unmet need. The development of therapies that target aberrant pathways thought to drive the development and growth of TNBC hold promise in improving long

term outcomes of such patients.

2.1.1 Heat Shock Protein 90

The highly abundant and ubiquitously expressed molecular chaperone, heat shock protein 90 (HSP90) is required for the functional stabilization of many client proteins, which include many oncogenic drivers (e.g. c-Kit, EGFR, ALK, androgen receptor) as well as proteins in key signaling pathways (e.g. protein kinase B [AKT]) [3-5]. Both HSP90α and heat shock protein 70 (HSP70) are induced under conditions of cellular stress to ensure the cell has an increased capacity to maintain proper protein folding. Heat shock factor-1 (HSF-1) is bound by HSP90 under non-stressed conditions. In response to a variety of cellular stresses (e.g. hypoxia, nutrient stress, proteotoxic stress and genetic instability), or direct inhibition of HSP90, HSF-1 dissociates from HSP90 and as a consequence upregulates HSP90α, HSP70, and HSP40 gene transcription.

In contrast to other chaperones, which generally act to aid the folding and refolding of proteins, HSP90 is only involved in the final maturation of a distinct set of "client" proteins. HSP90-dependent client proteins are involved in multiple processes such as growth factor independence, invasion and metastasis, sustained angiogenesis, cell survival, and resistance to anti-growth signals. The precise manner in which HSP90 influences the folding of these proteins is not fully understood, but the process is known to be adenosine triphosphate (ATP)-dependent. In the absence of HSP90, these clients are ubiquitinated and targeted for degradation via the proteasome [6].

Despite the ubiquitous expression of HSP 90 in all cells, cancer cells are thought to have a greater dependence on HSP90 than untransformed cells [7]. The increased levels of mutated proteins in tumor cells also potentially make them more dependent on HSP90 for protein stabilization. As a result, HSP90 is overexpressed and exists in a more active complex within tumor cells compared with normal cells [8, 9]. This form of HSP90 apparently has a greater affinity for HSP90 inhibitors, which have been observed to preferentially accumulate in xenograft tumor tissue.

2.1.2 Heat Shock Protein Inhibitors

Inhibition of HSP90 in tumor cells simultaneously affects multiple signaling pathways and processes within the cell. The treatment of tumor cells *in vitro* with AT13387 caused degradation of multiple known HSP90 client proteins and inhibition of cell signaling pathways known to be critical for cell growth and survival [10, 11]. Inhibition of HSP90 can bring about degradation of drug targets such as c-Kit, EGFR or ALK irrespective of resistance mutations whilst also affecting key signaling pathways, making this a potential method for inhibiting drug sensitive and resistant malignancy simultaneously[11].

HSP90 inhibitors, from several different classes, have been tested extensively in pre-clinical and clinical studies and shown to have anti-tumor activity [12, 13]. The first generation compounds were natural-product-derived ansamycins and have been used to validate HSP90 as a therapeutic target in many tumor types. Although clinical activity has been observed with these first-

generation compounds, they suffer from off-target toxicity and formulation issues, indicating a need for inhibitors with an improved pharmacological profile such as AT13387. AT13387 is a synthetic non-ansamycin potent HSP90 inhibitor (for more information, please see section 2.2).

2.1.3 Heat Shock Proteins and triple negative breast cancer

Heat Shock Protein 90 (HSP90) is a chaperon that participates in the proper folding and stabilization of many client proteins that are mediators of signal transduction known to be over-activated in triple negative breast cancer cell lines (such as AKT, EGFR, members of RAS/MAPK signaling pathways Androgen Receptor and others) [14]. Expression of HSP90 has been found to be upregulated in multiple triple negative breast cancer cell lines [15] and its over-expression has been associated with poor outcome of breast cancer patients for more information, please see section 9) · [9].

2.1.4 Heat Shock Protein 90 inhibitors in Triple Negative Breast Cancer

HSP90 inhibitors have been shown to retard growth of TNBC as shown in experiments in which MDA-MB-468, MDA-MB-231, and HCC-1806 human triple negative breast cancer cell lines were implanted and established in 4-6 week old *nu/nu* athymic mice that were subsequently treated with novel HSP90 inhibitor PU-H71. PU-H71 killed 80%, 65%, and 80% of the initial population of MDA-MB-468, MDA-MB-231, and HCC-1806 cells, respectively and was associated with G2-M cell cycle arrest. In comparison to other murine xenograft breast cancer models HU-H71 inhibited tumor growth of triple negative breast cancer to much higher extent compared to other types of breast cancer, such as HER-2/neu positive or hormone receptor positive types where this agent lead to only limited cytotoxic effects [16].

Multiple HSP90 inhibitors are currently in clinical development and are undergoing testing in phase I-III clinical trials. An HSP90 inhibitor ganetespib has been studied in a currently ongoing, open label phase 2 trial (ENCHANT 1) in patients with advanced triple negative or HER-2/neu positive breast cancer. As of summer 2014, the study showed that 18 of 31 triple negative breast cancer patients achieved metabolic response to single agent ganetespib (58%). Of 28 patients evaluable for response, clinical benefit rate was 50% (13 patients, including 2 patients with partial responses).

2.1.5 HSP90 Inhibitors and Taxanes

Paclitaxel is one of the most active agents in breast cancer and is often given early in the management of stage IV disease. Unfortunately, only approximately 40-50% of patients treated with paclitaxel in the first or second line setting have objective responses and all patients eventually develop progression after an average of 5-9 months of therapy [17]. In-vitro experiments demonstrated that activation of HSP90 client proteins, AKT and c-Raf confer resistance to tubulin polymerizing agents, including paclitaxel. This provides a rationale for combining paclitaxel with HSP90 inhibitors [18]. In pre-clinical studies, HSP90 inhibitors have been shown to sensitize breast cancer cells to paclitaxel. In a murine model of established breast

carcinoma (BT-474 breast cells), addition of HSP90 inhibitor 17-allylamino-17-demethoxygeldanamycin (17-AAG) at 50 mg/kg weekly in addition to paclitaxel at 25 mg/kg was associated with a mean of 83% reduction in tumor volume on day 35 of treatment and a complete response in 20% of the animals. In contrast, paclitaxel alone showed mainly stabilization of the tumor volume and no significant responses. Treatment with 17-AAG alone did not result in any growth inhibition in this tumor model [19]. In addition, experiments in murine models of CB.17/SCID female mice implanted with triple negative breast carcinoma cell line MDA-MB-231 demonstrated that combining HSP90 inhibitors with paclitaxel or docetaxel leads to significantly greater and more durable tumor responses than treatment with these chemotherapeutic agents alone [20]. In conclusion, the above pre-clinical evidence supports clinical development of HSP90 inhibitors in combination with paclitaxel in treatment of triple negative breast cancer. Such combination could increase sensitivity to paclitaxel leading to higher response rates and longer response duration.

2.2 CTEP IND Agent

2.2.1 AT13387

The following information about AT13387 is summarized from the investigator's brochure dated February 28, 2014.

AT13387 (2,4-dihydroxy-5-isopropyl-phenyl)-[5-(4-methyl-piperazin-1-ylmethyl)-1,3-dihydro-isoindol-2-yl]-methanone, L-lactic acid salt) is a synthetic non-ansamycin small molecule that acts as an inhibitor of heat shock protein 90 (HSP90) [21]. Its molecular weight is 499.61 kD. It is supplied as a sterile lyophilized solid in clear glass vials. Prior to administration, the lyophilized powder is to be reconstituted with D5W and then further diluted in D5W for infusion. Once reconstituted and diluted, AT13387 is administered to patients by peripheral or central IV infusion over 1 hour. Please refer to section 8.0 for additional information about AT13387.

2.2.1.1 Mechanism of Action

AT13387 binds to the ATP site on the N-terminal domain of the protein (dissociation constant [K_d]=0.71 nM) leading to improper folding of its client proteins and their degradation that results in growth arrest and apoptosis in a wide range of cancer cell lines as described below.

AT13387 was shown not to be a substrate of P-glycoprotein (Pgp) multidrug transporter and causes only modest inhibition of the transporter. In addition, based on investigator's brochure, AT13387 has no significant effects on inhibition or induction of cytochrome P450 (CYP), including 1A2, 3A4, 2D6, 2C9, and 2C19 at IC_{50} >10 µM.

2.2.1.2 Pre-Clinical Efficacy Studies

In-vitro studies demonstrated that AT13387 induces loss of oncogenic client proteins in low concentrations (30 – 100nM range). Mutated oncogenes such as BRAF or EGFR appear to be dependent on HSP90 for their stability. Preclinical *in vitro* and *in vivo* studies confirmed activity in multiple cell lines and animal tumor models, including lung cancer, hepatocellular carcinoma,

gastrointestinal stromal tumor, prostate cancer and breast cancer. *In vivo* studies also showed synergy of AT13387 with several small molecule inhibitors of tyrosine kinases that are clients of HSP90 such as crizotinib (NCI-H2228 - ALK-EML4 lung cancer cell lines with acquired resistance to crizotinib) and vemurafenib (SK-MEL-28R - BRAF mutated melanoma cell lines with acquired resistance to vemurafenib). These studies suggested that inhibition of HSP90 may result in increased sensitivity and delay in resistance to several anti-neoplastic agents. The combination of paclitaxel (20 mg/kg weekly) and AT13387 (55 mg/kg) was studied in human lung adenocarcinoma (NCI-H1975) xenograft models and led to complete tumor growth inhibition during the 3-week dosing period [22]. In studies involving BT474 breast carcinoma cell line (HER-2/neu over-expressing), AT13387 reduced the expression of oncoproteins that are common in malignant breast carcinoma cells such as HER2, CDK4 while inducing the compensatory expression of HSP70 [Please see investigator's brochure for more details].

2.2.1.3 Pre-clinical Pharmacokinetics and Metabolism of AT13387

In mice, the half-life of the terminal phase ranged from 0.9 to 3.2 hrs after a single IV bolus dose. AT13387 was administered to nude (Nu/Nu) Balb/c mice bearing HCT116 human colorectal carcinoma xenografts by the IP route and putative terminal half-life in plasma was similar to that observed after a single IV dose (4 vs. 3.2 hrs respectively). After 9 repeated doses following the schedule 3 days on treatment, 3 days off repeated 3 times by the IP route, the plasma half-life remained unchanged but the half-life in tumor tissue was substantially increased (Figure 2.2.1.3). The extensive distribution into and persistence in tumor tissue was thought likely to favor anti-tumor activity consistent with extended pharmacodynamic effects described previously.

Figure 2.2.1.3: Plasma, tumor and muscle compound concentration-time profile of AT13387 in Nu/Nu Balb/c Mice Bearing HCT116 xenografts after IP administration of 60 mg/kg followed over 14 days:

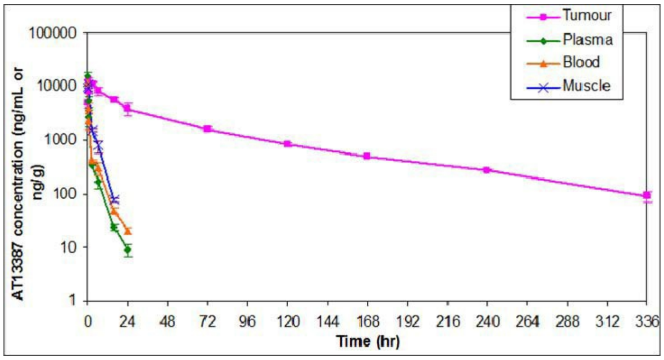

Concentration-time profile in muscle was assessed as a representative of normal tissue and the resultant half-life was 3 hrs. There was essentially no difference in the concentration-time profile for plasma or whole blood. Further distribution studies have shown that AT13387 has a similar PK profile in A375 (human melanoma cell line) and NCI-H1975 (human lung adenocarcinoma cell line) - xenograft-bearing mice. After IP administration of 80 mg/kg, the half-life of AT13387 in A375 tumors was up to 38 hrs (Study Report: MPK/2009/144) and up to 75 hrs in NCI-H1975 tumors (Study Report: DMPK/2009/165). Considerably shorter half-lives were observed in the normal tissues analyzed

Where measured, plasma clearance in the mouse was high, however, following a 50 mg/kg oral dose some bioavailability was observed (31%).

In rats, plasma compound concentration-time profiles appeared multi-phasic following IV bolus administration of doses ranging from 10 mg/kg to 200 mg/kg. The half-life of the terminal phase ranged from 1.2 to 4.5 hrs and was slightly longer after infusion (3.2 to 4.5 hrs) than bolus administration (1.2 to 1.6 hrs). The plasma clearance and volume of distribution at steady state (Vd_{ss}) appeared higher by IV infusion compared with IV bolus. In rats from the CNS study (CRL

JLS00035), exposure to AT13387 as measured by maximum concentration (C_{max}), increased in an approximately dose-related manner except between 90-125 mg/kg in females which was sub dose proportional. However, exposure as measured by area under concentration-time curve from time 0 to the last data point (AUC_{0-t}) increased in an approximately dose proportional manner. In females, the minimum and maximum exposure to AT13387 occurred at the lowest and highest dose of 10 and 125 mg/kg respectively. C_{max} and AUC_{0-t} values ranged from 1050 to 6890 ng/mL and 1450 to 19000 hr.ng/mL, respectively. For the single group of males (200 mg/kg), C_{max} and AUC_{0-t} values of 17000 ng/mL and 34100 hr.ng/mL were observed for AT13387. Gender differences in exposure to AT13387 could not be determined as male and female rats were not given comparable doses.

In Beagle dogs, the plasma compound concentration-time profile also appeared multi-phasic. When administered as an IV infusion, half-life appeared shorter at 4.5 hr than after bolus administration (half-life of 11 hr). In dogs from the CV/R study (CRL JLS00036), exposure to AT13387 increased with dose in an approximately dose-proportional manner across the dose range 1 to 15 mg/kg/dose. Mean C_{max} and AUC_{0-t} values ranged from 65.4 to 1930 ng/mL and 295 to 4570 hr.ng/mL for the dose range 1 to 15 mg/kg respectively. The % AUC extrapolated was low at <17%.

In all species assessed plasma clearance after IV bolus administration was high, generally at or above nominal liver blood flow (LBF) in each species. Clearance was calculated to be 112 to 200 mL/min/kg in the mouse, 60 to 76 mL/min/kg in the rat and 42 mL/min/kg in the dog.

In order to confirm the PK parameters derived from bolus IV administration, the PK properties were also examined after a 1hr IV infusion in the rat and dog. High plasma clearance was also observed in both species at greater than LBF, but interpretation of this finding is complicated by the potential for AT13387 to show some concentration-dependent partitioning into red-blood cells. For both species, clearance was slightly higher after IV infusion compared with bolus administration. The reason for this is not clear. Oral bioavailability of AT13387 in the rat was 9%, whereas in the dog it was 29% when dosed at 50 mg/kg and 1 mg/kg, respectively.

Volume of distribution at steady state (Vd_{ss}) was relatively high (greater than total body water) across all species assessed after both bolus and continuous IV infusion indicating distribution of AT13387 into tissues. AT13387 was essentially completely cleared from plasma by 24 hr (23 hr post-infusion) in both species.

2.2.1.4 Drug Interaction Studies

No formal pharmacodynamic drug interaction studies have been conducted with AT13387.

2.2.1.5 Distribution

Following incubations in mouse, rat, dog and human whole blood *in vitro*, the blood/plasma ratio of AT13387 across species ranged from 0.8, indicating approximately equal distribution between the plasma and cellular fraction, to 5.0, showing that AT13387 favored partitioning into the red blood cells. There was a concentration-dependent effect in the distribution of AT13387 in whole blood; the general trend was for AT13387 to have higher distribution into the cellular fraction at 0.2 μM compared with 2 μM. The binding to plasma proteins was assessed by equilibrium dialysis using mouse, rat, dog and human plasma. Results showed comparatively moderate binding in all species assessed, ranging from 77.2% in dog plasma to 90.1% in mouse plasma.

After a single IV 1-hr infusion to male Sprague-Dawley rats, [^{14}C]AT13387-derived radioactivity was detected in blood and plasma up to 24 hrs post-dose for male rats. The majority of the [^{14}C]AT13387-derived radioactivity excreted within 24 hrs. For the intact mass balance group, approximately 94% of the total [^{14}C]AT13387-derived radioactivity was excreted by 168 hrs, and the main route of excretion was through feces. For the bile-duct cannulated group, approximately 95% of the total [^{14}C] AT13387-derived radioactivity was excreted by 72 hrs and the main route of excretion was through bile.

After a single IV dose of [^{14}C]AT13387 to male rats, the tissue, plasma exposure ratios for [^{14}C]AT13387-derived radioactivity were ≥1.0 for most tissues, suggesting that high levels of [^{14}C]AT13387 and/or its metabolites were widely distributed to tissues in rats, regardless of strain. However, it was noted that the tissues involved in the endocrine, ocular, and secretory systems had higher exposure levels. The pattern of radioactivity distribution was consistent with the preferential uptake of [^{14}C]AT13387 by melanin-containing structures of the eye for pigmented rats. The majority of tissues for the male Sprague-Dawley (albino) rats did not have concentrations of radioactivity below the quantifiable limit at 24 hrs after dosing. The majority of tissues for the LE (pigmented) rats had concentrations of radioactivity below the limit of quantitation (BLQ) at 72 hrs after dosing; however, all tissues were BLQ by 504 hrs after dosing except for the adrenal gland, eye, meninges, retina, pituitary gland, and uveal tract.

Following a single 1-hour intravenous infusion of 3 mg/kg (20 μCi/kg) [^{14}C]AT13387 to male and female Beagle dogs (n = 1 each), [^{14}C]AT13387-derived radioactivity was measurable in blood and plasma up to 48 and 72 hr post-dose, respectively. The peak concentration for both genders was found at 1.0 hr when infusion stopped, and the C_{max} values of the plasma total radioactivity were determined at 721 ng-Eq/g and 795 ng-Eq/g for male and female dogs, respectively. The AUC_{0-t} values were 5924 hr•ng-Eq/g and 7590 hr•ng-Eq/g for male and female dogs, respectively, and the t½ values were 21.3 hr and 20.5 hr, respectively. The majority (approximately 83% in the male and 72% in the female) of [^{14}C]AT13387-derived radioactivity was excreted via feces, with less found in urine (approximately 15% in the male and 22% in the female). Up to 168 hr post-dose, approximately 97% of the total radioactivity dosed was recovered.

In conclusion, toxicokinetic data showed good characterization of AT13387 concentration-time profiles achieved for 1 hr IV infusion and plasma concentration-time profiles were consistent with this mode of administration. Exposure was confirmed in all test article dosed groups on Study Days sampled and increased an approximately dose-related manner. No AT13387 was detected in any control vehicle-dosed animal samples. There were no consistent gender differences. There was no significant accumulation or decrease in exposure upon repeat IV administration. AT13387 was essentially completely cleared from plasma by 24 hrs.

2.2.1.6 Metabolism

The *in vitro* intrinsic clearance was shown to be high across all the species tested (mouse, rat, dog and human) when incubated at 1 μM with intact viable hepatocytes. *In vivo* intrinsic clearance, normalized for liver size and body weight ("scaled"), ranged from 35 mL/min/kg in human to 184 mL/min/kg in rat, suggesting that AT13387 may have high metabolic clearance in animals and humans.

To determine UDP-glucuronosyltransferase (UGT) isoforms involved in the metabolism (glucuronidation) of AT13387, the compound was incubated with recombinant human UGT supersomes (microsomes prepared from baculovirus infected insect cells). Some degree of instability of AT13387 was observed during incubation in preparations of UGT1A1 and UGT1A3 (CL_{int} 21 and 18 μL/min/mg microsomal protein, respectively), with consistent, measurable depletion of AT13387 over the course of the incubations. The remaining three major isoforms tested, UGT1A6, UGT1A9, and UGT2B7 did not appear to measurably biotransform AT13387.

Preliminary non-GLP studies using non-radiolabeled AT13387 have been carried out to establish the routes of metabolism apparent in rat, dog and human cryopreserved hepatocytes. Early studies indicated the presence of glucuronide, sulphate and N-oxide metabolites.

The predominant metabolites in all species appeared to be conjugates of the resorcinol hydroxyl groups. N-oxidation of the methyl-piperazine ring also appeared to occur. In rat hepatocytes, the major metabolite appeared to be the sulphate conjugate M2 with minor amounts of glucuronide conjugate M3 and N-oxide M4. In dog hepatocytes, no putative metabolites reached 10% abundance although each of the metabolites M1-M4 were detected in minor amounts. In human hepatocytes, the major metabolite appeared to be the glucuronide conjugate M3 with the sulphate M2 and glucuronide M1 also present in minor amounts. Of the glucuronide regioisomers, human and dog hepatocytes produced the isomer M1, whereas the rat produced the isomer M2. The putative human metabolites detected were all represented in either rat or dog preparations or both.

In summary, the routes of AT13387 metabolism appear to be glucuronidation, sulphation and N-oxidation. The putative human metabolites detected were all represented in either rat or dog preparations or both.

2.2.1.7 Excretion

No formal excretion studies using AT13387 have been conducted.

2.2.1.8 Effect of AT13387 on Cytochrome P450 Enzymes

The potential of AT13387 to inhibit recombinant expressed human cytochromes P450 *in vitro* was examined as an indicator of potential drug-drug interactions. The potential for AT13387 to inhibit CYPs 1A2, 3A4, 2D6, 2C9 and 2C19 was assessed using fluorescent probe substrates. Results indicated a low potential for inhibition (IC_{50}>10μM), corresponding to a low potential for clinically significant drug-drug interactions mediated by these enzymes.

The potential of AT13387 to induce liver CYP enzymes 1A2, 2B6, and 3A4 in cultures of human hepatocytes (n=3 donors) was evaluated. The experiment was conducted at concentrations of 0.3, 3, and 30 μM. This concentration range did not result in cytotoxicity and was selected for this study based on emerging clinical exposure data available for AT13387. After 2 days of exposure, enzyme induction was determined in situ in conjunction with probe substrates selective for these drug-metabolizing enzymes and real time RT-PCR. AT13387 demonstrated no increase in CYP1A2, 2B6, and 3A4 activity in all 3 donors. The RT-PCR data corroborated the activity results for all CYP enzymes.

2.2.1.9 Summary of Pre-clinical Toxicology Studies

Two GLP compliant studies examined effect of AT13387 on central nervous system and cardiovascular/respiratory system in rats and dogs. Rats were dosed through a tail vein (early studies) or through a femoral vein (main studies). Dogs were dosed through a peripheral vein. The general toxicities in these studies are summarized in table 2.2.1.9:

Table 2.2.1.9: Summary of cardiovascular/respiratory and central nervous system safety and pharmacology studies in animals

**Type of Study**	**Species and Strain**	**Doses/Groups (mg/kg/dose)**	**Regimen (IV)**	**Key Findings**
Central Nervous System (CNS)<br><br>CRL JLS00035<br><br>GLP compliant	Sprague-Dawley Rats	0 (saline) (6M)<br>200 (6M)<br>0 (saline) (6F)<br>30 (6F)<br>90 (6F)<br>125 (6F)	Single 60-min infusion	Reduction in mean body temperature was observed after dosing at ≥90 mg/kg.<br><br>24 hr post-dose: body temperatures for treated groups were similar to controls.<br><br>Day 2: Body weight loss at ≥90 mg/kg compared to control. Soft or liquid feces observed in males at 200 mg/kg and females at 125 mg/kg. These findings were considered to be indicative of a systemic toxicity rather than a direct CNS effect.<br><br>No adverse CNS effects were noted at the doses studied.
Cardiovascular and Respiratory (CV/R)<br>CRL JLS00036<br>GLP compliant	Beagle Dogs (conscious telemetered)	One group of 4 non-naïve males, given 4 doses:<br>(1) saline<br>(2) 1<br>(3) 4<br>(4) 15	Four 60-min Infusions, approx. 1 week apart:	IV doses of AT13387 had no significant effect on arterial blood gases or QT interval or QTc at any dose tested.<br><br>Heart rate: Dose-related increase observed at 4 mg/kg and above from 10 minutes after the infusion start, which peaked at the end of infusion and returned to control levels by approximately 5 hrs after the infusion end. At 15 mg/kg, there was a concomitant decrease in blood pressure (systolic, diastolic, mean arterial).<br><br>At 15 mg/kg, respiratory rate and heart rate were decreased from 20-24 hrs after the end of infusion.<br><br>AT13387 was extensively eliminated from plasma by 23 hrs post-infusion, so the relationship of these findings to AT13387 was unclear. The increase in heart rate at ≥4 mg/kg and concomitant decrease in blood pressure appeared to be the primary AT13387 effect, corresponding to the maximal plasma compound concentration at the end of the IV infusion.

The remaining toxicology studies are summarized below:

The onset of AT13387 toxicity was characterized by adverse clinical signs, reduced food consumption, hematological and clinical chemistry effects. Data were obtained in an acute pilot study (clinical observations and necropsy), dose-range-finding studies (clinical observations and clinical pathology and necropsy), and definitive studies (full toxicological investigations). Results from these studies provide a clear picture of the safety and toxicity of AT13387 given by a 1-hr infusion.

The toxicity profile of AT13387 was similar in rats and dogs. Both species exhibited decreased body weight and food consumption, and decreases in white blood counts, as well as clinical pathology changes. The overall effects observed in surviving rats and dogs were transient and reversible, with the exception of the testicular lesions observed in dogs at high doses which had

not reversed during 14 days recovery.

In rats, AT13387 was administered over 1 hr, either through the tail vein or a femoral vein cannula, as a single dose or repeated. Both methods of administration were problematic in rats, as tail vein administration led to severe toxicity and femoral vein administration led to thrombus formation. In the definitive rat study, a surgically implanted femoral vein cannula was associated with thrombus formation and occasional early deaths, as confirmed by histopathologic investigations. The main results from these studies were the following:

- Clinical pathology changes suggestive of adverse effects in various organs including bone marrow, kidney and liver were observed at doses ≥50 mg/kg, but histopathology was not examined
- No unambiguous target organs of toxicity were identified, and the no observed adverse effect level (NOAEL) was estimated to be 50 mg/kg/dose given twice weekly for 3weeks
- Consequently a dose severely toxic to 10% of rodents (STD_{10}) could not be confidently determined in rats although safety was established in this species

In dogs, a clear dose-effect relationship was established for AT13387 (administration through a peripheral vein). Dogs were more sensitive than rats on a dose basis (mg/kg and mg/m^2) and an exposure basis (C_{max} and AUC). Histopathology in dogs revealed changes in the bone marrow, thymus, testes, gall bladder, and kidney at 3, 10/7, and 12.5/10/7 mg/kg. The nominal highest non-severely toxic dose (HNSTD) was 3 mg/kg/dose given twice weekly for 3 weeks. The NOAEL was taken to be 1mg/kg/dose on the same schedule.

A subsequent repeat-dose study of two new AT13387 formulations to assess potential acute toxicityand local infusion site irritation in rabbits revealed that higher pH (less acidic) phosphate-buffered formulation caused less infusion-site irritation. However, exposures in rabbits at the 25 mg/kg dose were more than 2-fold higher than in humans (at the MTD dose of 260 mg/m$_2$) and as a consequence, the repeat-dose regimen was not tolerated in rabbits, as mortalities occurred in all groups.

2.2.1.10 Justification of the Starting Dose for Human Studies

Due to adverse findings secondary to cannulation in rats, as well as no detection of unambiguous target organs of toxicity in rats, an STD_{10} could not be confidently determined in the rat. A clear dose-related toxicity was established in dogs, including identification of target organs of toxicity; the NOAEL was 1 mg/kg/dose given 2QWx3 and the nominal highest non-severely toxic dose (HNSTD) was 3 mg/kg/dose given 2QWx3. The dog was found to be more sensitive than the rat on a mg/kg and mg/m^2 basis, as well as in exposure terms (C_{max} and AUC).

The human clinical start dose was calculated on a mg/m^2 basis using accepted body surface area conversion factors [FDA, 2005]. Given its high sensitivity to AT13387, the dog was deemed an appropriate species for this purpose; therefore, the start dose was estimated as one-sixth of the nominal dog HNSTD (HNSTD = 3 mg/kg = 60 mg/m^2), giving a human start dose of 10 mg/m^2/dose given 2QWx3. The human start dose calculated from the dog HNSTD is 3-fold lower

than would be derived from the rat NOAEL (start dose based on one tenth of the rat NOAEL on a body surface area basis = 30 mg/m^2/dose).

2.2.1.11 Clinical Experience with AT13387

Three different schedules of AT13387 (administered as a 1 hour IV infusion) are being studied clinical trials:

- Schedule 1: Day 1, 8, 15 on a 28 daycycle
- Schedule 2: Twice weekly several days apart for 3 weeks (days 1, 4, 8, 11, 15, 18 on a 28 day cycle)
- Schedule 3: Daily for 2 consecutive days each week during weeks 1-3 of a 4 week cycle (days 1, 2, 8, 9, 15, 16 on a 28 day cycle).

As of 3/4/15, AT13387 was being studied in 6 clinical trials (including 1 NCI sponsored phase I trial):

1. AT13387-01 is a phase I trial in patients with treatment refractory solid tumors. Patients are treated with escalating doses of AT13387 monotherapy on schedule 1 or2.

2. AT13387-02 is a randomized phase 2 trial in patients with unresectable, malignant gastrointestinal stromal tumors after progression on 3 or less tyrosine kinase inhibitors. AT13387 is given at escalating doses alone or in combination with imatinib on schedule 1.

3. AT13387-03 CTEP 8828 is a phase I dose escalation study in patients with treatment refractorysolid tumors treated with AT13387 given in escalating doses on schedule 3. Dose expansion cohort at the maximum tolerated dose (MTD) of 160 mg/m^2 is currentlyaccruing patients with HER2 expressing tumors.

4. AT13387-04 is a phase 1/2 trial testing escalating doses of AT13387 as monotherapy or in combination with abiraterone patients with castration resistant prostate cancer that is no longer responding to single agent abiraterone. AT13387 is given on schedule 1 or 3.

5. AT13387-05 is a phase 1/2 study of escalating doses of AT13387 given as monotherapy on schedule 1 or in combination with crizotinib in patients with ALK+NSCLC.

6. A phase I study that enrolls patients with melanoma to the combination of AT13387 on schedule 1 and dabrafenib and trametinib.

Clinical Safety Profile:

The maximum tolerated doses of AT13387 have been established for all 3 schedules (260 mg/m^2, 120 mg/m^2, 160 mg/m^2 for schedules 1, 2, 3 respectively). DLTs that led to establishing these MTDs were as follows:

(1) The DLT in schedule 1 was a combined effect of moderate toxicities mostly grade 2 diarrhea, nausea, vomiting, fatigue and systemic infusion reactions.

(2) The DLT in schedule 2 was grade 3 visual impairment (fuzzy vision, green hue, and peripheral flashing) associated with grade 3 changes in electroretinogram.

(3) The DLT in schedule 3 was elevated transaminases.

In the twice-weekly dosing regimen, grade 3 blurred vision (chloropsia and photopsia) were seen at 120 mg/m^2 dosing cohort. Of note, mild (Grade 1 or 2) treatment-related visual disturbances were reported in approximately 45% of study patients. They appeared to be reversible in all subjects except for 1 subject treated at a dose of 220 mg/m^2 on schedule 1 who still had grade 1 visual impairment event (white spots) at the last follow up visit known to us as communicated during Project Team teleconferences (in July 2014). Approximately 30% of subjects experienced Grade 1 or 2 treatment-related systemic infusion reaction and injection site events which were manageable and led to the formulation change. Other common (>10%) grade 1-2 toxicities included diarrhea, fatigue, nausea, dizziness, dry mouth, anemia, muscle spasms, insomnia, abdominal pain, headache, rash, weight decreased, appetite, dysphonia, hemoglobin decreased, hypotension and pruritus. None of the deaths that occurred on the study were attributed to AT13387, although in one case (grade 5 metastatic colon cancer), the contribution of AT13387 could not be completely ruled out.

Human Pharmacokinetics (PK):
The PK of AT13387 showed dose-dependent increase in area under the plasma concentration-time curve from time 0 to the last data point ($AUC_{0\text{-}t}$). There was inter-individual variability of PKsby 2–5-fold for $AUC_{0\text{-}t}$ and elimination half-life. Maximum serum concentration (C_{max}) varied by up to 9-fold within the cohorts. The elimination half-life was dose-independent with mean cohort values ranging from 6.6 to 11.5 hours and maximum half-life of 14 hours. Plasma clearance of AT13387 was independent of dose with less than 5% of the administered dose excreted in the urine during 48 hours post-dose. The volume of distribution (V_d) was high (9.8-22.9 L/kg) suggesting high distribution of the agent to the extravascular space.

Clinical Activity:
Based on limited data from AT13387-01, AT13387-02 and AT13387-03 trials, responses were seen in treatment refractory patient population. In AT13387-01 trial 1 patient with GIST treated with weekly AT13387 (220 mg/m^2) had a partial response lasting 113 days and 2 patients had stable disease for over 6 months. One patient with metastatic melanoma and one patient with adenoid cystic adenoma had stable disease for over 6 months. Of 10 patients with non-small cell lung carcinoma, 4 had stable disease lasting 47-85 days. Mostly stable disease was observed in the other 2 trials.

2.3 Other Agent

2.3.1 Paclitaxel

Taxanes (including paclitaxel, docetaxel and *nab*-paclitaxel) are among the most active cytotoxic agents for the treatment of breast cancer [23-25]. Paclitaxel was originally isolated from the bark of the Pacific yew tree (*taxus brevifolia*) and subsequently found to have anti-tumor properties in the early 1970s [26, 27]. Its mechanism of action relates to binding and stabilization of microtubules causing inhibition of their depolymerization and leading to mitotic arrest [28]. Paclitaxel is hydrophobic and has poor solubility in water. Therefore this agent is solubilized in 50 % polyoxyethylated castor oil (Cremophor EL) and 50 % ethanol. Cremaphor EL vehicle is associated with hypersensitivity reactions which require premedication with corticosteroids and histamine receptor blockers to minimize its incidence and severity [29]. The realization that the majority of hypersensitivity reactions occur during the first 1-2 administrations of paclitaxel led to studies that demonstrated safety of withdrawing premedications in patients in whom hypersensitivity to paclitaxel formulation does not develop after the first 2 treatments [30].

Paclitaxel administered intravenously at a dose of 175 mg/m2 for 3 hours every third week emerged as an active and safe initial and salvage therapy for metastatic breast cancer with associated response rates around 30-40% and survival of about 19 months in patients with untreated metastatic breast cancer [23, 31]. Since the activity of paclitaxel is directly related to the cell cycle, shortening the interval between treatments, might improve efficacy [32]. In addition, paclitaxel administered in a more continuous manner exhibits proapoptotic and antiangiogenic properties, increasing its antineoplastic effects [33]. Indeed, subsequent randomized studies confirmed that weekly paclitaxel administration was superior to every-3-week schedules in treatment of metastatic and operable breast cancer [17, 34, 35]. Weeklypaclitaxel almost doubled the time to progression and increased the response rate from 29% to 42% without affecting quality of life in patients with metastatic breast cancer [17].

2.3.1.1 Adverse Events of Paclitaxel

The common adverse events of weekly paclitaxel are hypersensitivity reactions (including flushing, edema, hypotension, back pain), alopecia, rash, fatigue, nausea, diarrhea, sensory peripheral neuropathy, nail changes, bone marrow suppression (neutropenia, anemia and thrombocytopenia) and transient elevations in transaminases, alkaline phosphatase and bilirubin. About 3% of patients treated with paclitaxel can develop bradycardia. Hypersensitivity reactions are immediate and attributed to the vehicle cremaphor EL and can be mitigated by administration of diphenhydramine (25-50 mg IV), dexamethasone (20 mg IV) and famotidine (20 mg IV). Severe reactions occur in about 1-2% of patients who receive pre-mediation. When they occur, stopping the infusion of paclitaxel and administration of additional steroids and anti-histamines results in resolution of the symptoms in >90% of patients. Re-infusion of paclitaxel at 50% infusion rate with subsequent gradual escalation is commonly employed in routine practice and was found to be safe. Patients who do not experience hypersensitivity reactions during the first 1- 2 administrations are not likely to have them with subsequent infusions [30].

2.3.1.2 Dose and Schedule of Paclitaxel:

This study will utilize the weekly schedule of paclitaxel because it has been extensively studied and found to have superior efficacy to every 3 weekly paclitaxel [17, 34, 35]. There are some potential overlapping toxicities between AT13387 and paclitaxel, including infusion reaction (albeit it likely has a different etiology), diarrhea, nausea, fatigue, bone marrow suppression, transient LFTs elevation, rash, decrease appetite, dry mouth and vision disturbance. However, these toxicities are generally mild (≤ grade 2) and transient with paclitaxel dosed at 80 mg/m2 weekly. They are manageable with interventions [35]. In addition, this dose has been well tolerated in studies that used paclitaxel alone and in combinations with other chemotherapy agents (such as carboplatin) and targeted therapy (lapatinib, bevacizumab or trastuzumab) [36-38]. We are therefore proposing using the standard dose of paclitaxel at 80 mg/m2 on the weekly schedule. The information on safety profile of the combination and the effect of AT13387 on pharmacokinetics of paclitaxel will be collected in this trial. The dose escalation ofAT13387 will also provide safety and tolerability information of the combination treatment and will aim at establishing the maximum tolerated dose of AT13387 given together with standard dose and schedule of paclitaxel in patient with metastatic, triple negative breast cancer.

2.4 Rationale

Paclitaxel is one of the most active agents in breast cancer. Despite that, only approximately 40-50% of patients treated with paclitaxel for metastatic breast cancer in the first or second line setting will have an objective response [17]. Even if patients with metastatic breast cancer respond to paclitaxel, disease progression develops after a median of 5-9 months of therapy. Combining paclitaxel with agents that have the potential to increase the response rates and prolong response duration are greatly needed because they can lead to improvement in disease control (with possible improvement in cancer related symptoms) and potentially have a clinically significant impact on patient survival. Heat Shock Protein 90 (HSP90) is a chaperone that is expressed in triple negative breast cancers. Expression of HSP90 correlates with poorer survival of breast cancer patients. HSP90 has been shown to play an important role in post-translational modification necessary for proper function of multiple key oncoproteins (for example, EGFR, RAF, AKT, androgen receptor and others) responsible for tumor growth and resistance to conventional anti-neoplastic agents including paclitaxel. Preclinical studies showed that combining HSP 90 inhibitors with paclitaxel resulted in improved anti-tumor activity [19, 20]. **We hypothesize that combination therapy with HSP90 inhibitor, AT13387 and paclitaxel will have an acceptable toxicity profile and will result in durable responses in patients with unresectable, advanced triple negative breast cancer.**

Based on the above pre-clinical and clinical data, we propose a phase 1b clinical trial in patients with advanced triple negative breast cancer who have no curative treatment options. The study will test the combination of AT13387 and paclitaxel given on schedule 1 (see section 2.2.1.11) to establish safety profile and recommended phase 2 dose, measure the pharmacokinetics of each agent alone and in combination (to study the effect of each agent on the pharmacokinetics of the other agent) and preliminarily assess the response rate, response duration and progression free survival. Patients who previously received paclitaxel for metastatic breast cancer will be eligible

to participate in this trial, as long as they do not have grade >1 peripheral neuropathy and did not have disease progression on a taxane within the last 3 months. These patients would provide preliminary clinical data on whether combining HSP90 with paclitaxel could potentially lead to reversal of tumor resistance to paclitaxel.

2.5 Correlative Studies Background

The focus of this phase I trial will be assessment of safety, tolerability and determination of a recommended phase 2 dose of the study regimen. Given that this trial will enroll a small number of patients, extensive correlative studies will be deferred to a future phase 2 clinical trial. However, we are proposing a limited number of correlative studies that are outlinedbelow.

2.5.1 Pharmacokinetics

The effect of AT13387 on pharmacokinetics of paclitaxel and the effect of paclitaxel on pharmacokinetics of AT13387 will be evaluated to ensure that these agents do not have clinically significant interactions.

3. PATIENT SELECTION

3.1 Eligibility Criteria

3.1.1 Patients must have histologically confirmed measurable or unmeasurable advanced or metastatic breast cancer for which standard curative measures do not exist or are no longer effective.

Measurable disease is defined as at least one lesion that can be accurately measured in at least one dimension (longest diameter to be recorded for non-nodal lesions and short axis for nodal lesions) as ≥20 mm (≥2 cm) with conventional techniques or as ≥10 mm (≥1 cm) with spiral CT scan, MRI, or calipers by clinical exam. See Section 11 for the evaluation of measurable disease.

3.1.2 Primary and/or metastatic breast tumor must be negative for over-expression of estrogen and progesterone receptors. Patients with weak estrogen receptor and/or progesterone receptor expression (<10% on IHC) will be eligible.

3.1.3 Primary and/or metastatic breast tumor must be negative for HER-2/neu over-expression based on immunohistochemistry (IHC) (0 or 1+, 2+ if Fluorescence In-Situ Hybridization (FISH) test is negative) or FISH (HER2/CEP17 ratio <2.0 or <4 Her-2/neu signals per nucleus).

3.1.4 Any number of prior therapies for metastatic breast cancer is allowed. Patients with weakly estrogen receptor positive breast cancer who received any number of endocrine agents for metastatic breast cancer will also be eligible.

3.1.5 Prior taxane is allowed (as long as the patient is not experiencing grade >1 neuropathy and had no history of disease progression on a taxane therapy within 3 months prior to study enrollment).

3.1.6 Age ≥18 years. Based on Surveillance Epidemiology and End Results (SEER) database, approximately 1.9% of all cases of breast cancer are diagnosed in women that are younger than 25 years of age. Given that breast cancer is extremely rare in individuals younger than 18 years of age and because no dosing or adverse event data are currently available on the use of AT13387 in combination with paclitaxel in patients <18 years of age, children are excluded from this study but will be eligible for future pediatric trials.

3.1.7 ECOG performance status ≤2 (Karnofsky ≥60%, see APPENDIX A: PERFORMANCE STATUS CRITERIA).

3.1.8 Life expectancy of greater than 12 weeks

3.1.9 Patients must have normal organ and marrow function as defined below:

– leukocytes	≥2,000/μL
– absolute neutrophil count	≥1,500/μL
– platelets	≥100,000/μL
– total bilirubin	less than or equal to the institution's upper limit of normal
– AST(SGOT)/ALT(SGPT)	≤2.5 × institutional upper limit of normal (except for patients with liver metastases in whom AST/ALT can be <5 x institutional upper limit ofnormal)
– Creatinine within normal institutional limits OR creatinine clearance	≥50 mL/min for patients with creatinine levels above institutional normal
– Left ventricular ejection fraction of	>50% on baseline echocardiography or multi-gated acquisition (MUGA) scan
– QTc of	<480 milliseconds

3.1.10 Female subjects with child bearing potential must have a negative pregnancy test at screening. Child bearing potential is defined as sexually active patients with menses less than 1 year prior to enrollment, <65 years of age, have no history of oophorectomy or hysterectomy.

3.1.11 The effects of AT13387 on the developing human fetus are unknown. For this reason and because paclitaxel are known to be teratogenic, women of child-bearing potential and men must agree to use adequate contraception prior to study entry, for the duration of study

participation and 3 months after completion of study treatment administration. Adequate contraception includes methods such as oral contraceptives, double barrier method (condom plus spermicide or diaphragm), or abstaining from sexual intercourse. Should a woman become pregnant or suspect she is pregnant while she or her partner is participating in this study, she should inform her treating physician immediately.

3.1.12 Ability to understand and the willingness to sign a written informed consent document.

3.2 Exclusion Criteria

3.2.1 Patients who have had chemotherapy within 4 weeks (6 weeks for nitrosoureas or mitomycin C) prior to entering the study.

3.2.2 Patients who are receiving any other investigational agents within 4 weeks or 5 half-lives (whichever is shorter) prior to the first dose of the study regimen.

3.2.3 Prior radiation therapy within 2 weeks prior to the first dose of the study regimen.

3.2.4 Patients in whom prior treatment related toxicities have not recovered to grade 1 or less (except for alopecia).

3.2.5 Recent initiation of bone modifying therapy with a bisphosphonate or denosumab unless it has been started more than 4 weeks prior to the first dose of the study regimen. Patients who are already enrolled in this study can initiate bone modifying therapy after the first set of re-staging scans (≥8 weeks from cycle 1, day 1).

3.2.6 Prior therapy with AT13387 or another HSP90 inhibitor.

3.2.7 Patients with known brain metastases should be excluded from this clinical trial because of their poor prognosis and because they often develop progressive neurologic dysfunction that would confound the evaluation of neurologic and other adverse events. However, patients with previously treated and stable brain metastases are eligible as long as they are no longer requiring steroids, completed radiation therapy more than 2 weeks prior to the first dose of study regimen and have no seizures or worsening neurologic symptoms.

3.2.8 History of grade 3-4 immediate hypersensitivity reaction to paclitaxel.

3.2.9 History of clinically significant allergic reactions attributed to compounds of similar chemical or biologic composition to AT13387 or paclitaxel.

3.2.10 Based on investigator's brochure, AT13387 has no significant effects on inhibition or activation of cytochrome P450 (CYP), including 1A2, 3A4, 2D6, 2C9, and 2C19 at IC_{50} >10 µM. Preclinical studies indicated that AT13387 is only a modest inhibitor of P-gp. Paclitaxel is a substrate of CYP2C8 and CYP3A4. The use of CYP2C8 and CYP3A4

inhibitors/inducers while not prohibited in this study, is discouraged whenever feasible. Concurrent use of strong CYP2C8 and CYP3A4 inhibitors/inducers should be documented and the Principal Investigator (PI) of the study shall be notified prior to dosing. As part of the enrollment/informed consent procedures, the patients will be counseled on the risk of interactions with other agents, and what to do if new medications need to be prescribed or if the patient is considering a new over-the-counter medicine or herbal product. Refer to APPENDIX D: LIST OF CLINICALLY SIGNIFICANT OR STRONG CYP3A4 and CYP2C8 INHIBITORS AND INDUCERS for a list strong CYP2C8 and CYP3A4 inhibitors/inducers.

3.2.11 Uncontrolled intercurrent illness including, but not limited to, ongoing or active infection, symptomatic congestive heart failure, unstable angina pectoris, cardiac arrhythmia, or psychiatric illness/social situations that would limit compliance with studyrequirements.

3.2.12 Pregnant women are excluded from this study because Paclitaxel is a class D agent with the potential for teratogenic or abortifacient effects. Because there is an unknown but potential risk for adverse events in nursing infants secondary to treatment of the mother with AT13387 and paclitaxel, breastfeeding should be discontinued if the mother is treated with AT13387 and paclitaxel.

3.2.13 Patients who are HIV positive on highly active anti-retroviral therapy (HAART) will be excluded from the study because of the potential for pharmacokinetic interactions with AT13387. In addition, these patients are at increased risk of lethal infections when treated with marrow-suppressive therapy. Appropriate studies will be undertaken in patients receiving combination antiretroviral therapy when indicated.

3.2.14 Inability to understand and sign informed consent.

3.2.15 Any other medical or psychiatric condition that in the opinion of the investigator would make the study therapy unsafe for thepatient.

3.3 Inclusion of Women and Minorities

NIH policy requires that women and members of minority groups and their subpopulations be included in all NIH-supported biomedical and behavioral research projects involving NIH-defined clinical research unless a clear and compelling rationale and justification establishes to the satisfaction of the funding Institute & Center (IC) Director that inclusion is inappropriate with respect to the health of the subjects or the purpose of the research. Exclusion under other circumstances must be designated by the Director, NIH, upon the recommendation of an IC Director based on a compelling rationale and justification. Cost is not an acceptable reason for exclusion except when the study would duplicate data from other sources. Women of childbearing potential should not be routinely excluded from participation in clinical research. Please see http://grants.nih.gov/grants/funding/phs398/phs398.pdf.

4. REGISTRATION PROCEDURES

4.1 Investigator and Research Associate Registration with CTEP

Food and Drug Administration (FDA) regulations and National Cancer Institute (NCI) policy require all individuals contributing to NCI-sponsored trials to register and to renew their registration annually. To register, all individuals must obtain a Cancer Therapy Evaluation Program (CTEP) Identity and Access Management (IAM) account (https://ctepcore.nci.nih.gov/iam). In addition, persons with a registration type of Investigator (IVR), Non-Physician Investigator (NPIVR), or Associate Plus (AP) (i.e., clinical site staff requiring write access to OPEN or RAVE or acting as a primary site contact) must complete their annual registration using CTEP's web-based Registration and Credential Repository (RCR) (https://ctepcore.nci.nih.gov/rcr). Documentation requirements per registration type are outlined in the table below.

**Documentation Required**	**IVR**	**NPIV R**	**AP A**
FDA Form 1572	✔	✔	
Financial Disclosure Form	✔	✔	✔
NCI Biosketch (education, training, employment, license, and certification)	✔	✔	✔
HSP/GCP training	✔	✔	✔
Agent Shipment Form (if applicable)	✔		
CV (optional)	✔	✔	✔

An active CTEP-IAM user account and appropriate RCR registration is required to access all CTEP and CTSU (Cancer Trials Support Unit) websites and applications. In addition, IVRs and NPIVRs must list all clinical practice sites and IRBs covering their practice sites on the FDA Form 1572 in RCR to allow the following:

- Added to a site roster
- Assigned the treating, credit, consenting, or drug shipment (IVR only)tasks in OPEN
- Act as the site-protocol PI on the IRB approval
- Assigned the Clinical Investigator (CI) role on the Delegation of Tasks Log(DTL)

Additional information can be found on the CTEP website at

https://ctep.cancer.gov/investigatorResources/default.htm. For questions, please contact the RCR ***Help Desk*** by email at < RCRHelpDesk@nih.gov >.

4.2 Site Registration

This study is supported by the NCI Cancer Trials Support Unit (CTSU).

Each investigator or group of investigators at a clinical site must obtain IRB approval for this protocol and submit IRB approval and supporting documentation to the CTSU Regulatory Office before they can be approved to enroll patients.

Assignment of site registration status in the CTSU Regulatory Support System (RSS) uses extensive data to make a determination of whether a site has fulfilled all regulatory criteria including but not limited to the following:

- An active Federal Wide Assurance (FWA) number
- An active roster affiliation with the Lead Network or a participating organization
- A valid IRB approval
- Compliance with all protocol specific requirements

In addition, the site-protocol Principal Investigator (PI) must meet the following criteria:

- Active registration status
- The IRB number of the site IRB of record listed on their Form FDA 1572
- An active status on a participating roster at the registering site

Sites participating on the NCI CIRB initiative that are approved by the CIRB for this study are not required to submit IRB approval documentation to the CTSU Regulatory Office. For sites using the CIRB, IRB approval information is received from the CIRB and applied to the RSS in an automated process. Signatory Institutions must submit a Study Specific Worksheet for Local Context (SSW) to the CIRB via IRBManager to indicate their intent to open the study locally. The CIRB's approval of the SSW is then communicated to the CTSU Regulatory Office. In order for the SSW approval to be processed, the Signatory Institution must inform the CTSU which CIRB-approved institutions aligned with the Signatory Institution are participating in the study.

4.2.1 <u>Downloading Regulatory Documents</u>

Site registration forms may be downloaded from the NCI protocol # 9876 protocol page located on the CTSU Web site. Permission to view and download this protocol is restricted and is based on person and site roster data housed in the CTSU RSS. To participate, Investigators and Associates must be associated with the Corresponding or Participating protocol organization in the RSS.

- Go to https://www.ctsu.org and log in using your CTEP IAM username and password.

- Click on the Protocols tab in the upper left of your screen.
- Either enter the protocol # in the search field at the top of the protocol tree, or
- Click on the By Lead Organization folder to expand, then select LAO-OH007, and protocol #9876.
- Click on LPO Documents, select the Site Registration documents link, and download and complete the forms provided. (Note: For sites under the CIRB initiative, IRB data will load to RSS as described above.)

4.2.2 Submitting Regulatory Documents

Submit required forms and documents to the CTSU Regulatory Office, where they will be entered and tracked in the CTSU RSS.

Regulatory Submission Portal: www.ctsu.org (members' area) →Regulatory Tab →Regulatory Submission

When applicable, original documents should be mailed to:
CTSU Regulatory Office
1818 Market Street, Suite 3000
Philadelphia, PA 19103

Institutions with patients waiting that are unable to use the Portal should alert the CTSU Regulatory Office immediately at 1-866-651-2878 in order to receive further instruction and support

Requirements for 9876 Site Registration:

- IRB approval (For sites not participating via the NCI CIRB; local IRB documentation, an IRB-signed CTSU IRB Certification Form, Protocol of Human Subjects Assurance Identification/IRB Certification/Declaration of Exemption Form, or combination is accepted)

4.2.3 Checking Site Registration Status

You can verify your site registration status on the members' section of the CTSU website.

- Go to https://www.ctsu.org and log in to the members' area using your CTEP-IAM username and password
- Click on the Regulatory tab at the top of your screen
- Click on the Site Registration tab
- Enter your 5-character CTEP Institution Code and click on Go

Note: The status given only reflects compliance with IRB documentation and institutional

compliance with protocol-specific requirements as outlined by the Lead Network. It does not reflect compliance with protocol requirements for individuals participating on the protocol or the enrolling investigator's status with the NCI or their affiliated networks.

4.3 Patient Registration

4.3.1 General Registration Instructions

Astex Pharmaceuticals has discontinued the production of Onalespib (AT13387). Drug availability should be confirmed with CTEP for any patient who is considering enrollment on/after September 1st, 2019. Investigators of all open ("Active") trials are asked to change the protocol status to "Closed to Accrual" by December 1, 2019. All patients must be off-treatment by May 31, 2020.

Patients will be registered after meeting all entry requirements, clearance by the Protocol Coordinator, and signing of the informed consent.

OSU patients will be registered by the OSU research coordinator, as per their standard practice.

Subsite patients will have eligibility verified and will be entered on study centrally at the Ohio State University by the Multi-Institution Program Coordinator. All subsites must email theMulti-Institution Program Coordinator to verify slot availabilities prior to consenting patients. The required forms, including Eligibility Criteria Checklist and Registration Form, can be found in the Supplemental Forms Document.

To register a subsite patient, the following documents must be completed by the subsite research team and faxed or securely e-mailed to the Multi-Institution Program Coordinator:

- Copy of all baseline tests required per the protocol calendar. Tests must be within the specified window.
- Signed Patient Consent Form
- Signed Patient HIPAA Authorization Form
- Consent Documentation Note
- Completed & Signed Eligibility Checklist (refer to Supplemental Forms Document)
- Registration Form (refer to Supplemental Forms Document)
- Source documents verifying every inclusion & exclusion criteria

Note: every inclusion and exclusion criteria must be documented in the patient's medical record

Upon receipt of registration documents, the Multi-Institution Program Coordinator will send an email confirmation of receipt. If confirmation of receipt is not received within 1 hour of submission, please call or page the Multi-Institution Program Coordinator.

Upon receipt of all required registration documents and upon verification the subsite patient meets all eligibility criteria, the Multi-Institution Program Coordinator will:

- Assign the patient a study sequence ID

- Register the patient on the study
- Fax and/or e-mail to the subsite the completed Registration Form with the assigned study sequence ID as confirmation of patient registration

Each participating institution will order study agents directly. Agents may be ordered by a participating site only after the initial IRB approval for the site has been forwarded to the Multi- Institution Program Coordinator.

Patient sequence IDs will be assigned in the following fashion:

- A-BCD
  - A = CTEP Site ID
  - BCD = sequential numbers by order of enrollment

4.3.2 OPEN / IWRS

Patient enrollment will be facilitated using the Oncology Patient Enrollment Network (OPEN). OPEN is a web-based registration system available to users on a 24/7 basis. It is integrated with the CTSU Enterprise System for regulatory and roster data interchange and with the Theradex Interactive Web Response System (IWRS) for retrieval of patient registration. Patient enrollment data entered by Registrars in OPEN / IWRS will automatically transfer to the NCI's clinical data management system, Medidata Rave.

OSU will register all patients, including subsite patients, using IWRS.

For trials with slot reservation requirements, OPEN will connect to IWRS at enrollment initiation to check slot availability. Registration staff should ensure that a slot is available and secured for the patient before completing an enrollment.

The OPEN system will provide the site with a printable confirmation of registration and treatment information. Please print this confirmation for your records.

4.3.3 OPEN/IWRS User Requirements

OPEN/IWRS users must meet the following requirements:

- Have a valid CTEP-IAM account (*i.e.*, CTEP username and password).
- To enroll patients or request slot reservations: Be on an ETCTN Corresponding or Participating Organization roster with the role of Registrar. Registrars must hold a minimum of an AP registration type.
- To approve slot reservations or access cohort management: Be identified to Theradex as the "Client Admin" for the study.
- Have regulatory approval for the conduct of the study at their site.

Prior to accessing OPEN/IWRS, site staff should verify the following:

- All eligibility criteria have been met within the protocol stated timeframes.
- If applicable, all patients have signed an appropriate consent form and HIPAA

authorization form.

4.3.4 OPEN/IWRS Questions?

Further instructional information on OPEN is provided on the OPEN tab of the CTSU website at https://www.ctsu.org or at https://open.ctsu.org. For any additional questions contact the CTSU Help Desk at 1-888-823-5923 or ctsucontact@westat.com.

Theradex has developed a Slot Reservations and Cohort Management User Guide, which is available on the Theradex website: http://www.theradex.com/clinicalTechnologies/?National-Cancer-Institute-NCI-11.

This link to the Theradex website is also on the CTSU website OPEN tab. For questions about the use of IWRS for slot reservations, contact the Theradex Helpdesk: 609-619-7862 or Theradex main number 609-799-7580; CTMSSupport@theradex.com.

4.4 General Guidelines

Following registration, patients should begin protocol treatment within 14 days. Issues that would cause treatment delays should be discussed with the Principal Investigator. If a patient does not receive protocol therapy following registration, the patient's registration on the study may be canceled. The Study Coordinator should be notified of cancellations as soon as possible.

5. TREATMENT PLAN

5.1 Screening Procedures

After signing an informed consent, patients will be registered and will undergo screening evaluation. The following must be obtained within 14 days prior to the first dose of the investigational therapy:

- Demographics
- Concurrent medications
- Physical examination
- Vital signs
- Height
- Weight
- Eastern Cooperative Group performance status
- Complete blood count with differential
- Serum chemistry
- PTT and INR
- 12-lead EKG

- Pregnancy test in women with childbearing potential

In addition, the following screening procedures must be obtained within 28 days prior to the first dose of investigational therapy:

- Assessment of left ventricular systolic function (echocardiogram or multi-gated acquisition [MUGA] scan)
- CT scans of chest, abdomen and pelvis
- Whole body nuclear bone scan

Of note, other imaging at screening evaluation may be ordered if needed based on the investigator's discretion. Please see study calendar in section 10 for the summary of screening procedures.

5.2 Agent Administration

Patients who are eligible to continue on the study following screening evaluation will receive study therapy. Treatment will be administered on an outpatient basis. Reported adverse events and potential risks are described in Section 7. Appropriate dose modifications are described in Section **6.** No investigational or commercial agents or therapies other than those described below may be administered with the intent to treat the patient's malignancy.

The starting dose of AT13387 will be 120 mg/m^2. The dose of paclitaxel will be 80 mg/m^2 in all cohorts. Body Surface Area (BSA) calculations will be done using the Dubois formula for both agents. Dose will be calculated based on BSA on day 1 of every cycle, and dose rounding and dose adjustment due to weight change will be permitted based on institutional guidelines.

The following equation represents Dubois formula:

BSA(m2) = [height(in meters) x 100]$^{0.725}$ x weight(in kilograms)$^{0.425}$ x 0.007184

During Day -7 (+/-3 days), single agent AT13387 will be administered as an intravenous infusion given over approximately 1 hour (+/- 10 minutes) (safety run-in part).The dose will be based on the dose level as summarized in the table 5.2 below. Vital signs will be assessed prior to the start of AT13387 infusion, at 15, 30 minutes after start of infusion and immediately at the end of the effusion. Patients will remain and be observed in the infusion area for at least 90 minutes following the end of AT13387 administration.

Following the run-in part, administration schedule will consists of days 1, 8, and 15 on 28 day cycles. During Cycle 1, Day 1 only paclitaxel will be administered as a single agent at a dose of 80 mg/m^2 intravenously over approximately 1 hour (+/- 10 minutes) as per standard institutional administration guidelines. This is done to assess the effect of AT13387 on the pharmacokinetics of paclitaxel. Starting with Cycle 1, Day 8 and during all subsequent treatments, AT13387 and Paclitaxel will be given in combination. The figure below summarizes the schedule of the study therapy:

Figure 5.2:

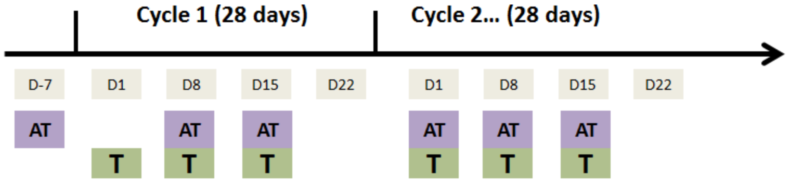

- AT 13387 will be given as a single agent on days -7 (+/- 3 days)
- Paclitaxel will be given as a single agent on day 1 of cycle 1.
- Paclitaxel and AT 13387 will be given on days 8, 15 on 28 day cycle (cycle 1)
- Paclitaxel and AT 13387 will be given on days 1, 8, 15 on 28 day cycle with subsequent cycles (starting with cycle 2).

D# Day of treatment and/or assessment

AT AT 13387 at escalating doses

T Paclitaxel at 80 mg/m2 IV

Since adequate data to indicate no major drug-drug interactions have already been collected, pharmacokinetics of paclitaxel and AT13387 became optional starting with protocol version 17 dated 05/03/2019. Patients who opt out from pharmacokinetic studies will not need to receive day -7 of AT13387 and can start both AT13387 and paclitaxel on day 1 of cycle 1.

When AT13387 and paclitaxel are given together, AT13387 will be given first as an intravenous infusion over approximately 1 hour (+/- 10 minutes). Vital signs will be assessed prior to administration of AT13387, at 15 and 30 minutes after the start of infusion of AT13387 and immediately after end of effusion of AT13387. Premedication for paclitaxel will be given at 30 minutes after completion of AT13387 administration per institutional guidelines. Paclitaxel will subsequently be administered at a dose of 80 mg/m^2 as an intravenous infusion over approximately 1 hour (+/- 10 minutes) as per standard institutional guidelines. Vital signs will be measuredprior to paclitaxel infusion, at 15, 30 minutes and immediately after end of infusion. Patients will be observed for 90 minutes after end of treatment. Treatment will be continued until disease progression, development of unacceptable adverse events, patient's decision to withdraw from the study or if the investigator determines that it is in the best patient's interest to discontinue the treatment (please see section 5.5 for more details). Dose escalation schema is presented in the following table.

Table 5.2 Dose Escalation Schedule:

Dose Escalation Schedule		
**Dose Level[b,c]**	**Dose[a]**	
	AT13387 (mg/m² IV on days 1, 8, 15)	***Paclitaxel (mg/m² IV on days 1, 8, 15)[d]***
Level -1	100	80
Level 1[e]	120	80
Level 2	150	80
Level 3	200	80
Level 4	260	80

[a]Doses are stated as exact dose in mg/m²
[b]Cycle length will be 28 days (except for safety run-in part when single agent AT13387 will be given on Day -7 +/- 3 days).
[c]Dose modifications or interruptions of AT13387 and Paclitaxel will not be allowed during the DLT period (cycle 1 of therapy) unless a patient experiences 1 or more dose limiting toxicities.
[d]In order to establish effects of AT13387 on pharmacokinetics of paclitaxel, paclitaxel will be given as a single agent during Cycle 1, Day 1.
[e]Starting dose

5.2.1 AT13387

The dose of AT13387 will be calculated based on patient's actual body weight. The Dose will need to be recalculated if patient's weight changes by >10%. AT13387 will be initially administered as a 250 mL IV infusion over approximately 1 hour (+/- 10 minutes).Treatment with AT13387 may be associated with local infusion-related irritation, as well as systemic infusion reactions, which occur either during the infusion or shortly afterwards (same day). The local infusion site irritation may be formulation-related (pH of current formulation ~5); Based on data from Studies AT13387-01 and -02, systemic reactions are often characterized by flushing, itching, rigors, chills, nausea, tachycardia/bradycardia, alterations in blood pressure and dizziness; events in AT13387-01 and -02 were reversible (data for AT13387 04 and -05 are not yet available). The incidence of these reactions increases at higher dose levels, and the severity. These effects may be reduced by slowing down the infusion or by IV hydration and pre-medications with dexamethasone, anti- histamines and 5HT3 inhibitors.

If extravasation is discovered or the patient develops localized infusion reaction the following cold compression protocol may be considered:

- □ Immediately after medical treatment is completed, apply ice pack to the affected for 15-20 minutes at least 4 times per day for the first 24-48 hours by any of the following means:
  a. Cool wash cloth
  b. Instant cool/ice pack

- □ The limb should be elevated at all times and exercised at least every 4-6 hours to

reduce immobility.

No pre-medications to prevent infusion reactions or nausea/vomiting of AT13387 would be required. If the patient develops systemic infusion reactions, the infusion may be diluted to 500 mL and/or premedication can be initiated. The suggested pre-medications would be dexamethasone 20 mg IV famotidine 20 mg IV, diphenhydramine 25-50 mg IV for allergic manifestations and IV ondansetron or similar agent for nausea/vomiting. These are suggestions and they can be modified based on treating physician's discretion and based on the specific systemic reaction symptoms.

5.2.2 Other Agent

Paclitaxel intravenous infusion will be given approximately 60-90 minutes after completion of AT13387 infusion. Given known risks of hypersensitivity to cremaphor EL in the formulation of paclitaxel, all patients will be pre-medicated prior to paclitaxel administration in order to prevent severe hypersensitivity reactions. Such premedication will consist of IV dexamethasone 10-20 mg, IV diphenhydramine (or its equivalent) 25-50 mg, and IV famotidine 20 mg 30 to 60minutes before paclitaxel. Paclitaxel will be administered as an IV infusion over approximately 1 hour (+/- 10 minutes) as per institutional standards for paclitaxel administration. Pre-medications can be discontinued after 2 paclitaxel treatments in patients who do not experience hypersensitivity reactions per discretion of the treating physician.

The management of infusion reaction and hypersensitivity reaction will be based on institutional standards. The table 5.2.2 below provides suggested management strategy.

Hypersensitivity Reactions: When hypersensitivity reactions occur to paclitaxel or its vehicle (Cremophor), they present almost universally during the first few minutes of infusion. Continued treatment may be considered only if the reactions are not life-threatening at treating physician's discretion; however, patients must be cautioned of potential recurrences of the reaction. Additional doses of steroids and famotidine (or equivalent) may be administered. The table below are suggested guidelines for management of patients with paclitaxel induced hypersensitivity reactions.

Table 5.2.2: Suggested management of hypersensitivity reactions during paclitaxel infusion.

Mild symptoms (Grade 1)	Moderate symptoms (Grade 2)	Severe symptoms (> Grade 2)
• *e.g* transient flushing, mild fever, mild rash<br>• May or may not interrupt infusion; observe patient until symptoms have resolved; no medical intervention required, resume and complete paclitaxel infusion	• *e.g.* rash, flushing, urticaria, shortness of breath, back pain, chest discomfort<br>• Interrupt paclitaxel infusion. Administer IV diphenhydramine 25 mg and IV dexamethasone 10-20 mg (or equivalent)<br>• Resume paclitaxel at a slower rate (25-50% previous rate) after symptoms resolved to < grade 1. If reactions did not recur, may escalate to original rate. Stop and discontinue paclitaxel if symptoms recur after re- challenge	• life-threatening symptoms; *e.g.* hypotension, angioedema, respiratory distress, anaphylaxis<br>• Stop and discontinue paclitaxel<br>• Treat and support according to institutional standards

5.3 Definition of Dose-Limiting Toxicity

Subjects who complete cycle 1 of therapy or those who have received at least 2 of the 3 doses of taxol and AT13387 in cycle 1 will comprise the DLT population, and this will help determine the recommended phase 2 dose (RP2D). Dose modifications or interruptions of AT13387 and Paclitaxel will not be allowed during the DLT period unless a patient experiences 1 or more dose limiting toxicities; or if the patient develops toxicities that require dose interruption based on dose modification tables in section 6, in which case either or both of the study medications may be held. If either study medication is held, all missed doses will not be made up upon resuming study therapy. Patients who do not complete the DLT period for reasons other than toxicities will be replaced. However, patients who develop toxicities that are at least possibly related to study therapy will not be replaced and will count towards DLT determination, even if they do not complete all treatments in cycle 1. Grading of toxicities will be assessed by the use of Common Terminology Criteria for Adverse Events (CTCAE) version 5.0. DLTs will be comprised of any adverse events that are at least possibly attributed to AT13387 and defined as follows:

Hematologic DLTs

- Grade 4 neutropenia lasting for ≥7 days in duration
- Grade ≥3 neutropenia complicated by a fever
- Grade 4 thrombocytopenia
- Grade 3 thrombocytopenia complicated by bleeding

Non-hematologic DLTs

- Any CTCAEv5.0 Grade ≥3 non-hematologic toxicity, unless the event is clearlyunrelated to treatment EXCEPT thefollowing:
  - Grade ≥3 nausea, vomiting, or diarrhea that resolves to Grade ≤2 within 48 hours, with or without medical intervention or prophylaxis
  - Grade 3 fatigue that resolves to Grade ≤2 within 14 days
  - Transient (<14 days) increase in LFTs (of ≤ one Grade in severity) compared to baseline levels in patients with baseline liver metastases.
  - Grade 3 maculopapular rash for which symptoms are easily managed with supportive care and no evidence of superinfection or limitation of self-care ADLs.

If, during a DLT period, a patient experiences a toxicity that does not clearly fit any of the above DLT criteria but which in the opinion of the investigator is highly clinically significant, the toxicity may be considered a DLT after a discussion with the Principal Investigator.

Management and dose modifications associated with the above adverse events are outlined in Section 6. Patients who experience dose limiting toxicities will be allowed to continue on study provided that (1) in the opinion of the investigator the benefit of continuing study therapy outweighs potential risks to the studysubject and the treatment related toxicities improve to grade 1 or less within 2 weeks of experiencing the event and (2) the case is discussed with the study Principal Investigator.

Dose escalation will proceed within each cohort according to the following scheme. Dose- limiting toxicity (DLT) is defined above.

Table 5.3 Dose escalation decision rules:

Number of Patients with DLT at a Given Dose Level	Escalation Decision Rule
0 out of 3	Enter 3 patients at the next dose level.
≥2	Dose escalation will be stopped. This dose level will be declared the maximally administered dose (highest dose administered). Three (3) additional patients will be entered at the next lowest dose level if only 3 patients were treated previously at that dose.
1 out of 3	Enter at least 3 more patients at this dose level.<br>• If 0 of these 3 patients experience DLT, proceedto the next dose level.<br>• If 1 or more of this group suffer DLT, then dose escalation is stopped, and this dose is declared the maximally administered dose. Three (3) additional patients will be entered at the next lowest dose level if only 3 patients were treated previously at that dose.
≤1 out of 6 at highest dose level below the maximallyadministered dose	This will be the recommended phase 2 dose. At least 6 patients must be entered at the maximum tolerated dose.

Once maximum tolerated dose is established, an additional 9 patients will be enrolled to that dose (for a total of 15 patients). This will allow the following: (1) further characterize the safety and tolerability of the combination; (2) provide additional data on effect of either agent on PK of the other agent and; (3) provide better estimates for anti-tumor activity with more precision levels. The toxicity monitoring guidelines for these additional 9 patients will be the same as for theother study patients. The DLT rate of ≥30 percent will be deemed unacceptable. Therefore, if 5 ormore of 15 patients develop a DLT, the protocol will be amended to consider dose expansion part in the dose level below previous maximum tolerated dose. However, if <5 of 15 patients develop the DLT, the dose will be declared as the recommended phase 2 dose.

5.3.1 Determination of Recommended Phase 2 Dose:

The R2PD will be defined as: (1) MTD, i.e. the highest doses of the combination of AT13387 and paclitaxel at which no more than one of 4 of 15 patients experiences a DLT or (2) doses of the combination below MTD, if in the opinion of the investigators, lower doses are better tolerated and safer. The first criterion will be given the priority when determining RP2D. The second criterion will only apply if an unexpected and highly clinically significant toxicity is noted that does not neatly fit the DLT criteria (for example, >1 patients in a given dose level develop grade 4 thrombocytopenia after cycle 2).

5.4 General Concomitant Medication and Supportive Care Guidelines

Preclinical data suggested that AT13387 has no significant effects on inhibition or activation of cytochrome P450 (CYP), including 1A2, 3A4, 2D6, 2C9, and 2C19 at IC_{50} >10 µM. Preclinical studies indicated that AT13387 is only a modest inhibitor of P-gp. Paclitaxel is a substrate of CYP2C8 and CYP3A4. The use of CYP2C8 and CYP3A4 inhibitors/inducers while not prohibited in this study, is discouraged whenever feasible. Concurrent use of strong CYP2C8 and CYP3A4 inhibitors/inducers should be documented and the Principal Investigator (PI) of the study shall be notified prior to dosing. As part of the enrollment/informed consent procedures, the patient will be counseled on the risk of interactions with other agents, and what to do if new medications need to be prescribed or if the patient is considering a new over-the-counter medicine or herbal product. APPENDIX B: INFORMATION ON POSSIBLE DRUG INTERACTIONS is a sample patient information sheet that can be tailored to this specific protocol and presented to the patient. Refer to APPENDIX D: LIST OF CLINICALLY SIGNIFICANT OR STRONG CYP3A4 and CYP2C8 INHIBITORS AND INDUCERS for a list strong CYP2C8 and CYP3A4 inhibitors/inducers.

Because there is a potential for interaction of AT13387 with other concomitantly administered drugs, the case report form must capture the concurrent use of all other drugs, over-the-counter medications, or alternative therapies.

Patients are not allowed to receive any investigational therapy other than AT13387 in combination with paclitaxel. Use of approved concurrent cytotoxic therapy (other than paclitaxel), endocrine therapy, radiation therapy or biologic therapy for breast cancer will also be prohibited. Supportive treatment should be given as per the institution standards and at the Investigator's discretion. Dexamethasone, 5HT3 inhibitors, and/or antihistamines could be given to treat or prevent the systemic infusion reactions per investigator's discretion. Antiemetics, anti-diarrheal agents, etc. may be given to treat or prevent gastrointestinal toxicities.

5.5 Duration of Therapy

In the absence of treatment delays due to adverse event(s), treatment may continue until one of the following criteria applies:

- Disease progression,
- Intercurrent illness that prevents further administration of treatment,
- Unacceptable adverse event(s),
- Patient decides to withdraw from the study, or
- General or specific changes in the patient's condition render the patient unacceptable for further treatment in the judgment of the investigator.
- All Patients must be off treatment by May 31, 2020.

5.6 Duration of Follow Up

Patients will be followed every 3 months after removal from study treatment or until progression or death, whichever occurs first. Patients removed from study for unacceptable adverse event(s) will be followed until resolution or stabilization of the adverse event.

5.7 Criteria for Removal from Study

Patients will be removed from study treatment when any of the criteria listed in Section 5.5 apply. However, patients who are removed from study treatment will continue to be followed for progression or death as specified in section 5.6. The followingcriteria will be used for removal of patients from the study: (1) patient withdraws their participation; (2) patient has a disease progression or dies (whichever occurs first); (3) the study is terminated by CTEP or the Principal Investigator. The reason for study removal and the date the patient was removed must be documented in the Case Report Form.

6. DOSING DELAYS/DOSE MODIFICATIONS

6.1 Dose Modifications of AT13387

Dose reductions or interruptions of AT13387 will not be permitted in cycle 1 unless a patient experiences a dose limiting toxicity. If the patient experiences a DLT during cycle 1, the treatment with AT13387 can be interrupted until the toxicity improves to grade 1 or lower; or if the patient develops toxicities that require dose interruption based on dose modification tables in section 6, in which case either or both of the study medications may be held. If either study medication is held, all missed doses will not be made up upon resuming study therapy. The study treatment can be resumed if (1) in the opinion of the investigator the benefit of continuing studytherapy outweighs potential risks to the study subject and the treatment related toxicities improve to grade 1 or less within 2 weeks of experiencing the event and (2) the case is discussed with the study Principal Investigator.

Following cycle 1, day 28, dose reductions or interruptions for adverse events may take place at any time. AT13387 may be held for up to 2 weeks in patients who experience grade 2 hematologic or non-hematologic toxicities per physician's discretion to manage intolerable or clinically significant toxicity. No dose reduction is required when resuming treatment. If either study medication is held, all missed doses will not be made up upon resuming studytherapy.

In patients who require dose reduction of AT13387, the dose would be reduced to one dose level below the current dose. For patients in dose level -1, AT13387 could be reduced to 80 mg/m^2. No reductions to dose below 80 mg/m^2 will be allowed. The dose modification guidelines are summarized in the table in section 6.3.

6.1.1. Dose reductions for QTc Elevations

A 12-lead ECG will be performed prior to the start of AT13387 infusion on day -7, cycle 1, day1 and day 1 of each subsequent cycles. If a subject's QTc is >480 msec but <500 msec, theinfusion will be withheld until the subject's QTc has decreased to ≤480 msec and then the subject may be dosed at the same dose level. If a subject's QTc is >500 msec, the treatment will be withheld until the subject's QTc has decreased to ≤480 msec and then the subject will be dosed at one dose level below their assigned treatment.

6.1.2. Visual Disturbances

Based on prior experience with AT 13387 on other phase I trials, approximately 45% of patients treated with the study drug experienced visual disturbances. These were predominantly mild (grade 1-2) and quickly reversible after the study agent has been discontinued. For that reason, anypatient who develops new visual impairment should be immediately referred to ophthalmology for an eye examination and follow up. Patients with grade 1 visual impairment will be allowed to continue study therapy. Patients who develop grade 2 or greater visual impairment will be managed based on table 6.3A below.

6.2 Dose Modifications of Paclitaxel

Dose modifications or interruptions of paclitaxel are not permitted in cycle 1 unless the patient experiences a dose limiting toxicity or if the patient develops toxicities that require dose interruption based on dose modification tables in section 6, in which case either or both of the study medications may be held. If either study medication is held, all missed doses will not be made up upon resuming study therapy. In order to receive weeklypaclitaxel the following criteria must be met:

- ANC ≥ 1000/µL
- Platelets ≥ 75,000/µL

If these criteria are not met, treatment must be delayed until counts recover to this level. Paclitaxel should be held for all instances of febrile neutropenia (ANC <1000/µL). Filgrastim growth factor support is encouraged for ANC <1500/µL and may be used at the discretion of the treating physician in order to maintain adequate blood counts EXCEPT during the first cycle. Filgrastim is not permitted within 24 hours prior to or following any paclitaxel infusion. Pegfilgrastim (Neulasta) is not permitted in this study.

If paclitaxel is held due to neutropenia, AT13387 may still be administered. The table below summarizes dose modifications of paclitaxel.

Dose Level	Paclitaxel Dose
-2	56 mg/m^2(30% reduction)
-1	64 mg/m^2 (20% reduction)
0	80 mg/m^2

Once dose has been reduced, dose re-escalation is not permitted.

6.3 Guidelines for dose modifications of AT13387 and Paclitaxel for toxicities

The following tables summarize guidelines for dose modifications of AT 13387 and Paclitaxel due to toxicities. Since each patient is unique and it is not possible to predict the risks of each individual case, the purpose of these guidelines is to provide a framework for dose modifications and should not replace the physician's experience and clinical judgment. The treating physician is allowed to hold or reduce the dose of both agents in patients who do not meet the above criteria IF in the opinion of the treating physician this would be required to ensure safety and reduce serious risks to the patient.

When paclitaxel is held due to an adverse event, AT13387 can be administered if in the opinion of the treating physician, the adverse event is clearly related to paclitaxel. Similarly, if AT13387 is held due to an adverse event, paclitaxel can be administered if in the opinion of the treating physician the adverse event is clearly related to AT13387. If the attribution of toxicity in not clear, both drugs should be held. If either study medication is held, all missed doses will not be made up upon resuming study therapy.

Table 6.3A: Recommended dose modifications of AT13387 and Paclitaxel for non-hematologic toxicities (excluding peripheral neuropathy):

**Non-hematologic Toxicities**	**Management/Next Dose for *AT13387*[a]**	**Management/Next Dose for Paclitaxel[a]**
Grade ≤1	No change in dose	No change in dose
Grade 2 (First occurrence)[b]	Hold* until grade ≤1. Resume at same dose level.	Hold until grade ≤1. Resume at same dose level.
Grade 2 (Second and subsequent occurrence)[b]	Hold* until grade ≤1. Resume at one dose level lower.**	Hold* until grade ≤1. Resume at one dose level lower.**
Grade 3 (First occurrence)	Hold* until grade ≤1.* Resume at one dose level lower.**	Hold* until grade ≤1. * Resume at one dose level lower.**
Grade 3 (Second occurrence)	Off protocol therapy	Off protocol therapy.
Grade 4	Off protocol therapy	Off protocol therapy
[a]Dose reduction applies only to the agent that is deemed to be the cause of the adverse event based on treating physician's opinion. If the attribution of the adverse event to either agent is not clear, both drugs should be held.<br>[b]Patients who develop grade 2 fatigue may continue treatment without dose reduction per discretion of treating physician.<br>*Patients requiring a delay of >2 weeks should go off protocol therapy.<br>**Patients requiring > two dose reductions of the same agent should go off protocol therapy.		

Table 6.3B: Recommended dose modifications of AT13387 and Paclitaxel for peripheral

neuropathy:

Peripheral Neuropathy	Management/Next Dose for *AT13387*[a]	Management/Next Dose for Paclitaxel[a]
≤ Grade 1	No change in dose	No change in dose
Grade 2	Hold* until grade ≤1. Resume at same dose level.	Hold until grade ≤1. Resume at one dose level lower.
Grade 2 (Second and subsequent occurrence)	Hold* until grade ≤1. Resume at one dose level lower.**	Hold* until grade ≤1. Resume at one dose level lower.**
Grade 3 (First occurrence)	Hold* until grade ≤1.* Resume at one dose level lower.**	Hold* until grade ≤1. * Resume at one dose level lower.**
Grade 3 (Second occurrence)	Off protocol therapy	Off protocol therapy.
Grade 4	Off protocol therapy	Off protocol therapy
[a]Dose reduction applies only to the agent that is deemed to be the cause of the adverse event based on treating physician's opinion. If the attribution of the adverse event to either agent is not clear, both drugs should be held.<br>*Patients requiring a delay of >2 weeks should go off protocol therapy.<br>**Patients requiring > two dose reductions of the same agent should go off protocol therapy.		

Table 6.3C: Dose modifications of AT13387 and Paclitaxel for patients who experience neutropenia:

Neutropenia	Management/Next Dose for AT13387[a]	Management/Next Dose for Paclitaxel[a]
≤ Grade 2	No change in dose	No change in dose
Grade 3 (First occurrence)	Hold* until grade ≤2. Resume at same dose level. Consideration may be given to growth factor support. If neutrophil count does not recover to ≤Grade 2 within 7 days, reduce by 1 dose level.**	Hold until grade ≤2. Resume at same dose level. Consideration may be given to growth factor support. If neutrophil count does not recover to grade ≤2 within 7 days, reduce by 1 dose level.**
Grade 3 (Second occurrence)	Hold* until grade ≤1. Resume at one dose level lower.** Provide growth factor support	Hold* until grade ≤1. Resume at one dose level lower.** Provide growth factor support
Grade 4	Off protocol therapy	Off protocol therapy
[a]Dose reduction applies only to the agent that is deemed to be the cause of the adverse event. If the attribution of the adverse event to either agent is not clear, both drugs should be held.<br>*Patients requiring a delay of >2 weeks of the same agent should go off protocol therapy.<br>**Patients requiring > two dose reductions of the same agent should go off protocol therapy.		

Table 6.3D: Dose modifications of AT13387 and Paclitaxel for patients who experience febrile neutropenia

Febrile Neutropenia	Management/Next Dose for AT13387	Management/Next Dose for Paclitaxel
Grade 3 or 4 (First and second occurrence)	Hold* until grade ≤2. Resume at the same dose level. If neutrophil count does not recover to grade ≤2, reduce by 1 dose level below.** Provide growth factor support.	Hold until grade ≤ 2. Resume at same dose level. If neutrophil count does not recover to grade ≤2 within 7 days, reduce by 1 dose level.** Provide growth factor support.
Grade 3 or 4 (second occurrence)	Hold* until grade ≤2. Resume at 1 dose level below.** Provide growth factor support.	Hold until grade ≤2. Resume at same dose level. If neutrophil count does not recover to grade ≤2 within 7 days, reduce by 1 dose level.** Provide growth factor support.
Grade 3 or 4 (Third occurrence)	Off protocol therapy	Off protocol therapy
[a]Dose reduction applies only to the agent that is deemed to be the cause of the adverse event. If the attribution of the adverse event to either agent is not clear, both drugs should be held.<br>*Patients requiring a delay of >2 weeks of the same agent should go off protocol therapy.<br>**Patients requiring > two dose reductions of the same agent should go off protocol therapy.		

Table 6.3E: Dose modifications of AT13387 and Paclitaxel for other hematologic toxicities

<u>Other Hematologic</u> Toxicities[a]	Management/Next Dose for *AT13387*[b]	Management/Next Dose for Paclitaxel[b]
≤ Grade 1	No change in dose	No change in dose
Grade 2 (First occurrence)	Hold until grade ≤1. * Resume at same dose level.	Hold until grade ≤1. Resume at same dose level.
Grade 2 (Second and subsequent occurrence)	Hold* until grade ≤1. Resume at one dose level lower.**	Hold* until grade ≤1. Resume at one dose level lower.**
Grade 3 (First occurrence)	Hold* until grade ≤1.* Resume at one dose level lower.**	Hold* until grade ≤1 * Resume at one dose level lower.**
Grade 3 (Second occurrence)	Off protocol therapy	Off protocol therapy.
Grade 4	Off protocol therapy	Off protocol therapy

[a]These guidelines exclude anemia for which dose adjustments are left to the discretion of the treating physician. Supportive care such as pRBC transfusion for symptomatic anemia or hgb <7g/dL is suggested. In addition, grade 2 leukopenia is not a criterion for holding or reducing the dose of study therapy.<br>[b]Dose reduction applies only to the agent that is deemed to be the cause of the adverse event based on treating physician's opinion. If the attribution of the adverse event to either agent is not clear, both drugs should be held.<br>[*]Patients requiring a delay of >2 weeks of the same agent should go off protocol therapy.<br>[**]Patients requiring > two dose reductions of the same agent should go off protocol therapy.

7. ADVERSE EVENTS: LIST AND REPORTING REQUIREMENTS

Adverse event (AE) monitoring and reporting is a routine part of every clinical trial. The following list of AEs (Section 7.1) and the characteristics of an observed AE (Section 7.2) will determine whether the event requires expedited reporting via the CTEP Adverse Event Reporting System (CTEP-AERS) **in addition** to routine reporting.

7.1 Comprehensive Adverse Events and Potential Risks List(s) (CAEPRs)

The Comprehensive Adverse Events and Potential Risks list (CAEPR) provides a single list of reported and/or potential adverse events (AE) associated with an agent using a uniform presentation of events by body system. In addition to the comprehensive list, a subset, the Specific Protocol Exceptions to Expedited Reporting (SPEER), appears in a separate column and is identified with bold and italicized text. This subset of AEs (SPEER) is a list of events that are protocol specific exceptions to expedited reporting to NCI (except as noted below). Refer to the 'CTEP, NCI Guidelines: Adverse Event Reporting Requirements' http://ctep.cancer.gov/protocolDevelopment/electronic_applications/docs/aeguidelines.pdf for further clarification. The CAEPR does not provide frequency data; refer to the Investigator's Brochure for this information. Below is the CAEPR for AT13387 (Onalespib).

NOTE: Report AEs on the SPEER **ONLY IF** they exceed the grade noted in parentheses next to the AE in the SPEER. If this CAEPR is part of a combination protocol using multiple investigational agents and has an AE listed on different SPEERs, use the lower of the grades to determine if expedited reporting is required.

7.1.1 CAEPRs for CTEP IND Agent(s)

7.1.1.1 CAEPR for AT13387

Comprehensive Adverse Events and Potential Risks list (CAEPR)
for AT13387 (Onalespib, NSC 749712)

The Comprehensive Adverse Events and Potential Risks list (CAEPR) provides a single list of reported and/or potential adverse events (AE) associated with an agent using a uniform presentation of events by body system. In addition to the comprehensive list, a subset, the Specific Protocol Exceptions to Expedited Reporting (SPEER), appears in a separate column and is identified with bold and italicized text. This subset of AEs (SPEER) is a list of events that are

protocol specific exceptions to expedited reporting to NCI (except as noted below). Refer to the 'CTEP, NCI Guidelines: Adverse Event Reporting Requirements' http://ctep.cancer.gov/protocolDevelopment/electronic_applications/docs/aeguidelines.pdf for further clarification. *Frequency is provided based on 119 patients.* Below is the CAEPR for AT13387 (Onalespib).

NOTE: Report AEs on the SPEER **ONLY IF** they exceed the grade noted in parentheses next to the AE in the SPEER. If this CAEPR is part of a combination protocol using multiple investigational agents and has an AE listed on different SPEERs, use the lower of the grades to determine if expedited reporting is required.

Version 2.1, December 28, 2018[1]

Adverse Events with Possible Relationship to AT13387 (Onalespib) (CTCAE 5.0 Term) [n= 119]			Specific Protocol Exceptions to Expedited Reporting (SPEER)
**Likely (>20%)**	**Less Likely (<=20%)**	**Rare but Serious (<3%)**	
BLOOD AND LYMPHATIC SYSTEM DISORDERS			
Anemia			***Anemia (Gr 2)***
EYE DISORDERS			
	Blurred vision		
	Vision decreased		***Vision decreased (Gr 2)***
GASTROINTESTINAL DISORDERS			
	Abdominal pain		***Abdominal pain (Gr 2)***
	Constipation		***Constipation (Gr 2)***
Diarrhea			***Diarrhea (Gr 2)***
	Dry mouth		***Dry mouth (Gr 2)***
	Dyspepsia		***Dyspepsia (Gr 2)***
	Flatulence		***Flatulence (Gr 2)***
	Gastrointestinal hemorrhage[2]		
	Hemorrhoids		***Hemorrhoids (Gr 2)***
Nausea			***Nausea (Gr 2)***
	Vomiting		***Vomiting (Gr 2)***
GENERAL DISORDERS AND ADMINISTRATION SITE CONDITIONS			
	Edema limbs		
Fatigue			***Fatigue (Gr 2)***
	Fever[3]		***Fever[3] (Gr 2)***
Injection site reaction[4]			***Injection site reaction[4] (Gr 2)***
	Malaise		***Malaise (Gr 2)***
INFECTIONS AND INFESTATIONS			
	Infection[5]		***Infection[5] (Gr 2)***
INJURY, POISONING AND PROCEDURAL COMPLICATIONS			
	Infusion related reaction[3]		***Infusion related reaction[3] (Gr 2)***
INVESTIGATIONS			
	Alanine aminotransferase increased		***Alanine aminotransferase increased (Gr 2)***
	Alkaline phosphatase increased		***Alkaline phosphatase increased (Gr 2)***
	Aspartate aminotransferase increased		***Aspartate aminotransferase increased (Gr 2)***
	CPK increased		***CPK increased (Gr 2)***
	Electrocardiogram QT corrected interval prolonged		

Adverse Events with Possible Relationship to AT13387 (Onalespib) (CTCAE 5.0 Term) [n= 119]			Specific Protocol Exceptions to Expedited Reporting (SPEER)
**Likely (>20%)**	**Less Likely (<=20%)**	**Rare but Serious (<3%)**	
	Lymphocyte count decreased		***Lymphocyte count decreased (Gr 2)***
	Platelet count decreased		***Platelet count decreased (Gr 2)***
	Weight loss		***Weight loss (Gr 2)***
	White blood cell decreased		
METABOLISM AND NUTRITION DISORDERS			
	Anorexia		***Anorexia (Gr 2)***
	Dehydration		***Dehydration (Gr 2)***
	Hypocalcemia		***Hypocalcemia (Gr 2)***
	Hypokalemia		
	Hypomagnesemia		
	Hyponatremia		***Hyponatremia (Gr 2)***
MUSCULOSKELETAL AND CONNECTIVE TISSUE DISORDERS			
	Muscle cramp		***Muscle cramp (Gr 2)***
	Myalgia		***Myalgia (Gr 2)***
NERVOUS SYSTEM DISORDERS			
	Dizziness		***Dizziness (Gr 2)***
	Dysgeusia		
	Headache		***Headache (Gr 2)***
PSYCHIATRIC DISORDERS			
	Insomnia		***Insomnia (Gr 2)***
RESPIRATORY, THORACIC AND MEDIASTINAL DISORDERS			
	Cough		***Cough (Gr 2)***
	Dyspnea		***Dyspnea (Gr 2)***
	Hiccups		***Hiccups (Gr 2)***
SKIN AND SUBCUTANEOUS TISSUE DISORDERS			
	Dry skin		***Dry skin (Gr 2)***
	Hyperhidrosis[3]		***Hyperhidrosis[3] (Gr 2)***
	Rash acneiform		
	Rash maculo-papular		***Rash maculo-papular (Gr 2)***
VASCULAR DISORDERS			
	Flushing		***Flushing (Gr 2)***

[1]This table will be updated as the toxicity profile of the agent is revised. Updates will be distributed to all Principal Investigators at the time of revision. The current version can be obtained by contacting PIO@CTEP.NCI.NIH.GOV. Your name, the name of the investigator, the protocol and the agent should be included in the e-mail.

[2]Gastrointestinal hemorrhage includes Anal hemorrhage, Cecal hemorrhage, Colonic hemorrhage, Duodenal hemorrhage, Esophageal hemorrhage, Esophageal varices hemorrhage, Gastric hemorrhage, Hemorrhoidal hemorrhage, Ileal hemorrhage, Intra-abdominal hemorrhage, Jejunal hemorrhage, Lower gastrointestinal hemorrhage, Oral hemorrhage, Pancreatic hemorrhage, Rectal hemorrhage, Retroperitoneal hemorrhage, and Upper gastrointestinal hemorrhage under the GASTROINTESTINAL DISORDERS SOC.

[3]Infusion-related reactions may include, tachycardia/bradycardia, hypotension/hypertension, flushing, chills, fever, hyperhidrosis, itching, rigors, and abdominal cramps.

[4]Injection site reaction may include injection site irritation, injection site pain, injection site inflammation or redness, or erythema.

[5]Infection may include all 75 sites of infection under the INFECTIONS AND INFESTATIONS SOC.

Adverse events reported on AT13387 (Onalespib) trials, but for which there is insufficient evidence to suggest that there was a reasonable possibility that AT13387 (Onalespib) caused the adverse event:

BLOOD AND LYMPHATIC SYSTEM DISORDERS - Febrile neutropenia
CARDIAC DISORDERS - Cardiac disorders - Other (atrioventricular block NOS); Left ventricular systolic dysfunction; Palpitations
EYE DISORDERS - Dry eye; Eye disorders - Other (color distortion); Eye disorders - Other (diplopia); Eye disorders - Other (halos); Eye disorders - Other (loss of visual acuity during changes in ambient light levels); Eye disorders - Other (tunnel vision); Eye disorders - Other (visual color darkening); Eye disorders - Other (visual disturbances); Eye pain; Flashing lights; Floaters; Keratitis; Night blindness; Papilledema; Photophobia; Retinopathy
GASTROINTESTINAL DISORDERS - Colitis; Mucositis oral; Oral dysesthesia; Oral pain; Salivary duct inflammation
GENERAL DISORDERS AND ADMINISTRATION SITE CONDITIONS - Chills[3]; Flu like symptoms
HEPATOBILIARY DISORDERS - Hepatic hemorrhage
INVESTIGATIONS - Activated partial thromboplastin time prolonged; Blood bilirubin increased; Creatinine increased; Ejection fraction decreased; Neutrophil count decreased
METABOLISM AND NUTRITION DISORDERS - Hyperglycemia; Hypoalbuminemia; Hypophosphatemia
MUSCULOSKELETAL AND CONNECTIVE TISSUE DISORDERS - Back pain; Bone pain; Generalized muscle weakness
NERVOUS SYSTEM DISORDERS - Seizure; Syncope; Tremor
PSYCHIATRIC DISORDERS - Anxiety
RENAL AND URINARY DISORDERS - Acute kidney injury; Proteinuria
RESPIRATORY, THORACIC AND MEDIASTINAL DISORDERS - Pneumonitis
SKIN AND SUBCUTANEOUS TISSUE DISORDERS - Palmar-plantar erythrodysesthesia syndrome; Pruritus; Skin hyperpigmentation
VASCULAR DISORDERS - Hypertension[3]

Note: AT13387 (Onalespib) in combination with other agents could cause an exacerbation of any adverse event currently known to be caused by the other agent, or the combination may result in events never previously associated with either agent

There are no genotoxicity, carcinogenicity, developmental and reproductive studies conducted with AT13387. Women of childbearing potential should not become pregnant or breastfeed and men should not father a child during the study. All subjects must use acceptable contraceptive measures during the treatment of AT13387 and 3 months after the last dose of the investigational drug.

If a patient is suspected to be pregnant, AT13387 should be IMMEDIATELY discontinued and the study physician contacted. A positive urine test must be confirmed by a serum pregnancy test. If it is confirmed that the patients is not pregnant, the patient may resume dosing with AT13387.

If a female patient becomes pregnant during therapy or within 3 months after the last dose of AT13387, or if the female partner of a male patient exposed to the drug becomes pregnant while the

male patient is receiving AT13387 or within 3 months after the last dose of AT13387, the investigator must be notified in order to facilitate outcome follow-up.

- Abortion, whether accidental, therapeutic, or spontaneous, should always be classified as serious. Any congenital anomaly/birth defect in a child conceived during the study or within 12 months after the last dose of AT13387 to a female patient or to a female partner of a male patient exposed to the agent during treatment or within 12 months after the last dose of AT13387 should be recorded and reported as an SAE.

- Female patients should not breastfeed a baby while on this study.

- Female patients must NEVER donate ova while or after being treated with AT13387.

All sexually active male patients should utilize a barrier form of contraception **during study treatment and for 3 months after the last dose** as it is not known whether AT13387 that may be present in seminal fluid would cause teratogenic effects in a fetus born to the female partner of a male patient. Males should also not donate sperm during treatment or up to 12 months after the last dose.

All patients are prohibited from donating blood while on study and for 12 months after the last dose of AT13387.

Local infusion-related irritation and systemic infusion reactions may occur during or shortly after the administration of AT13387. The local infusion adverse events are a formulation-related (pH of current formulation is ~ 4.2). Systemic adverse events (e.g.; flushing, itching, rigors, chills, nausea, tachycardia/bradycardia, dizziness) are reversible. If that occurs, slow the infusion rate and/or hydrate with D5W. Premedication with dexamethasone, antihistamine and 5HT3 antagonists can also be given.

Adverse events also reported on AT13387 (Onalespib) trials but with the relationship to AT13387 (Onalespib) still undetermined:

BLOOD AND LYMPHATIC SYSTEM DISORDERS - Febrile neutropenia
CARDIAC DISORDERS - Atrioventricular block first degree; Cardiac disorders - Other (atrioventricular block NOS); Left ventricular systolic dysfunction; Palpitations; Sinus bradycardia
EAR AND LABYRINTH DISORDERS - Ear and labyrinth disorders - Other (motion sickness); Ear and labyrinth disorders - Other (right ear sensation of blockage)
EYE DISORDERS - Dry eye; Eye disorders - Other (color distortion); Eye disorders - Other (diplopia); Eye disorders - Other (halos); Eye disorders - Other (loss of visual acuity during changes in ambient light levels); Eye pain; Flashing lights; Keratitis; Night blindness; Papilledema; Photophobia; Retinopathy
GASTROINTESTINAL DISORDERS - Abdominal distension; Colitis; Dysphagia; Gastrointestinal disorders - Other (altered saliva); Oral dysesthesia; Oral pain; Salivary duct inflammation
GENERAL DISORDERS AND ADMINISTRATION SITE CONDITIONS - Edema face; Flu like symptoms
INVESTIGATIONS - Creatinine increased; Ejection fraction decreased; INR increased
METABOLISM AND NUTRITION DISORDERS - Hyperkalemia; Hypermagnesemia; Hypoalbuminemia; Hypoglycemia; Hypophosphatemia
MUSCULOSKELETAL AND CONNECTIVE TISSUE DISORDERS - Arthralgia; Chest wall pain; Generalized muscle weakness; Pain in extremity
NERVOUS SYSTEM DISORDERS - Dysesthesia; Paresthesia; Seizure; Syncope; Tremor

PSYCHIATRIC DISORDERS - Confusion; Depression
RENAL AND URINARY DISORDERS - Acute kidney injury
RESPIRATORY, THORACIC AND MEDIASTINAL DISORDERS - Nasal congestion; Pneumonitis; Sinus disorder
SKIN AND SUBCUTANEOUS TISSUE DISORDERS - Palmar-plantar erythrodysesthesia syndrome; Pruritus; Skin hyperpigmentation
VASCULAR DISORDERS - Hypotension

Note: AT13387 (Onalespib) in combination with other agents could cause an exacerbation of any adverse event currently known to be caused by the other agent, or the combination may result in events never previously associated with either agent.

7.1.1.2 CAEPR for CTEP IND Agent #2

Not Applicable

7.1.2 Adverse Event List(s) for Other Investigational Agent(s)

Not Applicable

7.1.3 Adverse Event List(s) for Paclitaxel

Consult the package insert for the most current and comprehensive list of side effects.

Hematologic: The most common dose limiting toxicityis myelosuppression, primarilyleukopenia.

Anaphylaxis and severe hypersensitivity reactions: Patients with a history of severe hypersensitivity reactions to products containing Cremophor® EL (e.g., cyclosporin for injection concentrate and teniposide for injection concentrate) should not be treated with paclitaxel. In order to avoid the occurrence of severe hypersensitivity reactions, all patients treated with paclitaxel should be premedicated with corticosteroids (such as dexamethasone), diphenhydramine and H2 antagonists (such as famotidine or ranitidine). Since most of these reactions occur during the first 2 infusions, discontinuation of these premedications may be a consideration at the discretion of the treating physician. Minor symptoms such as flushing, skin reactions, dyspnea, hypotension, or tachycardia do not require interruption of therapy. However, severe reactions, such as hypotension requiring treatment, dyspnea requiring bronchodilators, angioedema, or generalized urticaria require immediate discontinuation of paclitaxel and aggressive symptomatic therapy. Patients who have developed severe hypersensitivity reactions should not be re-challenged withpaclitaxel.

Cardiac: Cardiovascular events observed with paclitaxel include hypotension and bradycardia. Severe conduction abnormalities have been documented in < 1% of patients during Paclitaxel therapy and in some cases requiring pacemaker placement. If patients develop significant conduction abnormalities during paclitaxel infusion, appropriate therapy should be administered and continuous cardiac monitoring should be performed during subsequent therapy with

paclitaxel.

Neurologic: The frequency and severity of neurologic events are dose-dependent. Peripheral neuropathy is rarely severe and may be the cause of paclitaxel discontinuation in 1% of patients. Sensory symptoms usually improve or resolve within several months of completion of treatment. Serious neurologic events such as grand mal seizures, syncope, ataxia and neuroencephalopathy are rare. Although the occurrence of peripheral neuropathy is frequent, the development of severe symptomatology is unusual and requires a dose reduction of 20% or greater for all subsequent courses of paclitaxel.

Gastrointestinal: The most common GI toxicities, which include nausea, vomiting, diarrhea and mucositis, are typically mild or moderate in severity. These respond well to supportive care with anti- emetics such as prochlorperazine, anti-diarrheal agents such as loperamide and topical analgesics for mucositis. Weekly administration of paclitaxel at 80 mg/m^2 is rarely associated with nausea/vomiting and antiemetic pre-medication is generally not needed prior to infusion. If patient experience significant nausea/vomiting during or post-infusion, appropriate antiemetic(s) may be added as prophylaxis prior to infusion at the treating physician's discretion.

Injection Site Reaction: Injection site reactions, including reactions secondary to extravasation, were usually mild and consisted of erythema, tenderness, skin discoloration, or swelling at the injection site. These reactions have been observed more frequently with the 24-hour infusionthan with the 3-hour infusion. Recurrence of skin reactions at a site of previous extravasation following administration of paclitaxel at a different site, i.e., ―recall‖, has been reported rarely. Rare reports of more severe events such as phlebitis, cellulitis, induration, skin exfoliation, necrosis, and fibrosis have been received as part of the continuing surveillance of paclitaxel safety. In some cases the onset of the injection site reaction either occurred during a prolonged infusion or was delayed by a week to ten days. Given the possibility of extravasation, it is advisable to closely monitor the infusion site for possible infiltration during drug administration. If extravasation is discovered or the patient develops localized infusion reaction the following cold compression protocol is recommended:

- Immediately after medical treatment is completed, apply ice pack to the affected for 15-20 minutes at least 4 times per day for the first 24-48 hours by any of the following means:
    a. Cool wash cloth
    b. Instant cool/ice pack
- The limb should be elevated at all times and exercised at least every 4-6 hours to reduce immobility.

Other: Although 60% of all patients experience arthralgia and myalgia, there is no consistent relationship between the dose or schedule of paclitaxel and the frequency or these events. The symptoms, which usually begin 2 or 3 days after paclitaxel treatment, are generally transient. Almost all patients receiving paclitaxel experience alopecia. Nail changes (changes in pigmentation or discoloration of nail bed; lifting of the nails) are uncommon with frequency of

about 2%. Occasionally, edema is seen at a rate of about 5%. It is usually of mild severity and gradually reversible following discontinuation of paclitaxel.

Pregnancy: Paclitaxel can cause fetal harm when administered to a pregnant woman. Administration of paclitaxel during the period of organogenesis to rabbits at doses of 3.0 mg/kg/day (about 0.2 the daily maximum recommended human dose on a mg/m2 basis) caused embryo and fetotoxicity, as indicated by intrauterine mortality, increased resorptions, and increased pregnancy loss. There are no adequate and well-controlled studies in pregnant women. If paclitaxel is used during pregnancy, or if the patient becomes pregnant while receiving this drug, the patient should be apprised of the potential hazard to the fetus. Women of childbearing potential should be advised to avoid becoming pregnant.

Carcinogenesis, Mutagenesis, Impairment of Fertility: The carcinogenic potential of paclitaxel has not been studied. Paclitaxel has been shown to be clastogenic *in vitro* (chromosome aberrations in human lymphocytes) and *in vivo* (micronucleus test in mice). Paclitaxel was not mutagenic in the Ames test or the CHO/HGPRT gene mutation assay. Administration of paclitaxel prior to and during mating produced impairment of fertility in male and female rats at doses equal to or greater than 1 mg/kg/day (about 0.04 the daily maximum recommended human dose on a mg/m2 basis). At this dose, paclitaxel caused reduced fertility and reproductive indices, and increased embryo- and fetotoxicity.

Pregnancy: Pregnancy — “Category D”.

7.2 Adverse Event Characteristics

CTCAE term (AE description) and grade: The descriptions and grading scales found in the revised NCI Common Terminology Criteria for Adverse Events (CTCAE) version 4.0 will be utilized until March 31, 2018 for AE reporting. CTCAE version 5.0 will be utilized for AE reporting beginning April 1, 2018. All appropriate treatment areas should have access to a copy of the CTCAE version 5.0. A copy of the CTCAE version 5.0 can be downloaded from the CTEP web site http://ctep.cancer.gov/protocolDevelopment/electronic_applications/ctc.htm.

- **For expedited reporting purposes only:**
  - AEs for the agent that are ***bold and italicized*** in the CAEPR (*i.e.*, those listed in the SPEER column, Section 7.1.1) should be reported through CTEP-AERS only if the gradeis above the grade provided in the SPEER.
  - Other AEs for the protocol that do not require expedited reporting are outlined in section 7.3.4.

- **Attribution** of the AE:
  - Definite – The AE *is clearly related* to the studytreatment.
  - Probable – The AE *is likely related* to the studytreatment.
  - Possible – The AE *may be related* to the studytreatment.
  - Unlikely – The AE *is doubtfully related* to the studytreatment.
  - Unrelated – The AE *is clearly NOT related* to the studytreatment.

7.3 Expedited Adverse Event Reporting

7.3.1 Expedited AE reporting for this study

Expedited AE reporting for this study must use CTEP-AERS (CTEP Adverse Event Reporting System), accessed via the CTEP Web site (https://eapps- ctep.nci.nih.gov/ctepaers). The reporting procedures to be followed are presented in the "NCI Guidelines for Investigators: Adverse Event Reporting Requirements for DCTD (CTEP and CIP) and DCP INDs and IDEs" which can be downloaded from the CTEP Web site (http://ctep.cancer.gov/protocolDevelopment/electronic_applications/adverse_events.htm). These requirements are briefly outlined in the tables below (Section 7.3.3).

In the rare occurrence when Internet connectivity is lost, a 24-hour notification is to be made to CTEP by telephone at 301-897-7497. Once Internet connectivity is restored, the 24-hour notification phoned in must be entered electronically into CTEP-AERS by the original submitter at the site.

7.3.2 Distribution of Adverse Event Reports

CTEP-AERS is programmed for automatic electronic distribution of reports to the following individuals: Principal Investigator and Adverse Event Coordinator(s) (if applicable) of the Corresponding Organization or Lead Organization, the local treating physician, and the Reporter and Submitter. CTEP-AERS provides a copy feature for other e-mail recipients.

The Coordinating Center of the Corresponding Organization is responsible for submitting to the CTSU documentation of AEs that they deem reportable for posting on the CTSU protocol web page and inclusion on the CTSU bi-monthly broadcast.

7.3.3 Expedited Reporting Guidelines

Use the NCI protocol number and the protocol-specific patient ID assigned during trial registration on all reports.

Note: A death on study requires both routine and expedited reporting, regardless of causality. Attribution to treatment or other cause must be provided.

Death due to progressive disease should be reported as **Grade 5 "Disease progression"** in the system organ class (SOC) "General disorders and administration site conditions." Evidence that the death was a manifestation of underlying disease (*e.g.*, radiological changes suggesting tumor growth or progression: clinical deterioration associated with a disease process) should be submitted.

Phase 1 and Early Phase 2 Studies: Expedited Reporting Requirements for Adverse Events that Occur on Studies under an IND/IDE within 30 Days of the Last Administration of the Investigational Agent/Intervention [1]

FDA REPORTING REQUIREMENTS FOR SERIOUS ADVERSE EVENTS (21 CFR Part 312)

NOTE: Investigators **MUST** immediately report to the sponsor (NCI) **ANY** Serious Adverse Events, whether or not they are considered related to the investigational agent(s)/intervention (21 CFR 312.64)

An adverse event is considered serious if it results in **ANY** of the following outcomes:

1) Death
2) A life-threatening adverse event
3) An adverse event that results in inpatient hospitalization or prolongation of existing hospitalization for ≥ 24 hours
4) A persistent or significant incapacity or substantial disruption of the ability to conduct normal life functions
5) A congenital anomaly/birth defect.
6) Important Medical Events (IME) that may not result in death, be life threatening, or require hospitalization may be considered serious when, based upon medical judgment, they may jeopardize the patient or subject and may require medical or surgical intervention to prevent one of the outcomes listed in this definition. (FDA, 21 CFR 312.32; ICH E2A and ICH E6).

ALL SERIOUS adverse events that meet the above criteria MUST be immediately reported to the NCI via electronic submission within the timeframes detailed in the table below.

Hospitalization	Grade 1 and Grade 2 Timeframes	Grade 3-5 Timeframes
Resulting in Hospitalization ≥ 24 hrs	10 Calendar Days	24-Hour 5 Calendar Days
Not resulting in Hospitalization ≥ 24 hrs	Not required	

NOTE: Protocol specific exceptions to expedited reporting of serious adverse events are found in the Specific Protocol Exceptions to Expedited Reporting (SPEER) portion of the CAEPR.

Expedited AE reporting timelines are defined as:

- "24-Hour; 5 Calendar Days" - The AE must initially be submitted electronically within 24 hours of learning of the AE, followed by a complete expedited report within 5 calendar days of the initial 24-hour report.
- "10 Calendar Days" - A complete expedited report on the AE must be submitted electronically within 10 calendar days of learning of the AE.

[1]Serious adverse events that occur more than 30 days after the last administration of investigational agent/intervention and have an attribution of possible, probable, or definite require reporting as follows:

Expedited 24-hour notification followed by complete report within 5 calendar days for:

- All Grade 3, 4, and Grade 5 AEs

Expedited 10 calendar day reports for:

- Grade 2 AEs resulting in hospitalization or prolongation of hospitalization

Effective Date: May 5, 2011

7.3.4 Additional Protocol-Specific Expedited Adverse Event Reporting Exclusions

All SAEs, regardless of causality, must be reported to the OSU PI and Multi-Institution Coordinator within 24 hours of knowledge of the event. Initial 24 hour notification using secure email or fax is acceptable. For SAEs not requiring expedited reporting via CTEP-AERS, a complete report accompanied by the SAE Submission Form (refer to Supplemental Forms Document) must be submitted to the OSU PI and Multi-Institution Coordinator via secure email or fax within 5 days of knowledge of the event.

All sites will directly report SAEs requiring expedited reporting to CTEP as outlined in the previous sections.

7.4 Routine Adverse Event Reporting

All Adverse Events **must** be reported in routine study data submissions. **AEs reported expeditiously through CTEP-AERS must also be reported in routine study data submissions.**

Adverse event data collection and reporting, which are required as part of every clinical trial, are done to ensure the safety of patients enrolled in the studies as well as those who will enroll in future studies using similar agents. AEs are reported in a routine manner at scheduled times during the trial using Medidata Rave. For this trial the Adverse Event CRF is used for routine AE reporting in Rave.

7.5 Secondary Malignancy

A secondary malignancy is a cancer caused by treatment for a previous malignancy (*e.g.*, treatment with investigational agent/intervention, radiation or chemotherapy). A secondary malignancy is not considered a metastasis of the initial neoplasm.

CTEP requires all secondary malignancies that occur following treatment with an agent under an NCI IND/IDE be reported expeditiously via CTEP-AERS. Three options are available todescribe the event:

- Leukemia secondary to oncology chemotherapy (*e.g.*, acutemyelocytic leukemia [AML])
- Myelodysplastic syndrome (MDS)
- Treatment-related secondary malignancy

Any malignancy possibly related to cancer treatment (including AML/MDS) should also be reported via the routine reporting mechanisms outlined in each protocol.

7.6 Second Malignancy

A second malignancy is one unrelated to the treatment of a prior malignancy (and is **NOT** a metastasis from the initial malignancy). Second malignancies require **ONLY** routine AE reporting unless otherwise specified.

8 PHARMACEUTICAL INFORMATION

A list of the adverse events and potential risks associated with the investigational and commercial agents administered in this study can be found in Section 7.1.

8.1 CTEP IND Agent

8.1.1. AT13387 (NSC 749712)

Chemical Name: (2,4-dihydroxy-5-isopropyl-phenyl)-[5-(4-methyl-piperazin-1-ylmethyl)-1,3-dihydro-isoindol -2-yl]-methanone, *L*-lactic acid salt

Other Name: AT13387AU, Onalespib

Classification: Heat shock protein 90 (HSP90) inhibitor

Molecular Formula: $C_{24}H_{31}N_3O_3.C_3H_6O_3$ **M.W.:** 499.61

Mode of Action: AT13387 is a synthetic non-ansamycin small molecule that inhibits heat shock protein 90 (HSP90. HSP90 seems to affect multiple aberrant signaling pathways and therefore may be of clinical benefit in several cancer treatments.

How Supplied: AT13387 is supplied by Astex Pharmaceuticals Inc. and distributed by CTEP, NCI as a 265 mg free base equivalent (L-lactic acid salt) vial, containing white to off-white lyophilized powder. The agent is formulated in **pH 5.0 (red cap).**

Preparation: Reconstitute the 265-mg lyophilized powder with 10 mL of Sterile Water for Injection (SWFI) resulting in 25.7 mg/mL concentration (10.3 mL total volume). A sticky mass will be formed. Vigorously shake the vial. Agitate until the contents are fully dissolved (about 5 minutes). Leave the diluted vial at ambient temperature for 15-30 minutes to allow any foam to dissipate. If not used immediately, store the reconstituted vial(s) at 2° to 8° C not to exceed 8 hours.

Withdraw the calculated dose of AT13387 and further dilute it in 250 mL of D5W or 0.9% NS. The prepared IV solution is compatible in PVC or non-PVC infusion bags.

Store the prepared IV solution at 2° to 8° C (not to exceed 8 hours) if not used immediately. When removed from the refrigerator, allows the prepared IV solution to sit at room temperature between 15 to 30 minutes before administering to patients. The prepared IV solution must be used within 24 hours -i.e., from the time the drug vial is diluted to the time the IV administration is complete. Protection from light during the infusion period is not required

Storage: Store the intact vials at 15^0 to 25ºC (59 to 77ºF). Protect from light.

If a storage temperature excursion is identified, promptly return AT13387 to 15^0 to 25ºC (59 to 77ºF) and quarantine the supplies. Provide a detailed report of the excursion (including documentation of temperature monitoring and duration of the excursion) to PMBAfterHours@mail.nih.gov for determination of suitability.

Stability: Shelf life surveillance of the intact vials is ongoing**.**
Route of Administration: Intravenous

Method of Administration: Infuse over 1 hour through a central line or a well-defined peripheral vein (Note: an in-line filter is NOT required). If use a peripheral line, be sure to aspirate venous blood prior to starting the infusion. Check the infusion site every 15 minutes. Change infusion site should evidence of swelling or discoloration is observed.

Potential Drug Interactions: AT13387 is a substrate of UGT with a relatively low affinity for UGT isoforms. In vitro data demonstrate that AT13387 is a weak inhibitor of UGT1A1, UGT1A3 and UGT1A9. AT13387 is also a weak inhibitor of CYP1A2, -3A4, -2D6, -2C9 and -2C19. AT13387 appears to metabolize via the glucuronidation, sulphation and N-oxidation.

Pre-clinical studies suggest that AT13387 is a substrate of P-gp, the efflux ratios was above 2 (ranging from 3.4 to 4.6); a moderate inhibitor of BCRP (35.9% +/- 2%, p=0.0001) and P-gp (31.3% +/- 1.2%, p= 0.0009), and a strong inhibitor of MATE1 (94.6% +/- 0.2%, p=0.0001) and MATE2-K (91.2% +/- 1.2%, p= 0.0002).

Patient Care Implications: There are no genotoxicity, carcinogenicity, developmental and reproductive studies conducted with AT13387. Women of childbearing potential should not become pregnant or breastfeed and men should not father a child during the study. All subjects must use acceptable contraceptive measures during the treatment of AT13387 and 3 months after the last dose of the investigational drug.

Systemic infusion reactions (e.g., vomiting, itching skin, or swelling), slow the infusion and/or administer NS or D5W through a "Y" connector in parallel to AT13387 IV infusion. Pre-medication (e.g., dexamethasone, H-1 and H-2 antagonist) may be given before subsequent infusions and/or administer additional volume of 500 mL over 1 hour if medically appropriate.

Avoid extravasation. For local irritation, apply cold compress or topical pain medication. Change infusion site if any evidence of swelling or discoloring is observed.

Availability

AT13387 is an investigational agent supplied to investigators by the Division of Cancer Treatment and Diagnosis (DCTD), NCI.

AT13387 is provided to the NCI under a Collaborative Agreement between the Pharmaceutical Collaborator and the DCTD, NCI (see Section 12.3).

8.1.2 CTEP IND Agent #2

Not Applicable

8.1.3 Agent Ordering and Agent Accountability

8.1.3.1 NCI-supplied agents may be requested by the responsible investigator (or their authorized designee) at each participating institution. Pharmaceutical Management Branch (PMB) policy requires that agent be shipped directly to the institution where the patient is to be treated. PMB does not permit the transfer of agents between institutions (unless prior approval from PMB is obtained). The CTEP-assigned protocol number must be used for ordering all CTEP-supplied investigational agents. The responsible investigator at each participating institution must be registered with CTEP, DCTD through an annual submission of FDA Form 1572 (Statement of Investigator), Biosketch, Agent Shipment Form, and Financial Disclosure Form (FDF). If there are several participating investigators at one institution, CTEP-supplied investigational agents for the study should beordered under the name of one lead investigator at that institution.

Submit agent requests through the PMB Online Agent Order Processing (OAOP) application. Access to OAOP requires the establishment of a CTEP Identity and Access Management (IAM) account and the maintenance of an "active" account status, a "current" password, and active person registration status. For questions about drug orders, transfers, returns, or accountability, call or email PMB any time. Refer to the PMB's website for specific policies and guidelines related to agentmanagement.

8.1.3.2 Agent Inventory Records – The investigator, or a responsible party designated by the investigator, must maintain a careful record of the inventory and disposition of all agents received from DCTD using the NCI Drug Accountability Record Form (DARF). (See the NCI Investigator's Handbook for Procedures for Drug Accountability and Storage.)

8.1.4 <u>Investigator Brochure Availability</u>

The current versions of the IBs for the agents will be accessible to site investigators and research staff through the PMB OAOP application. Access to OAOP requires the establishment of a CTEP IAM account and the maintenance of an "active" account status, a "current" password and active person registration status. Questions about IB access may be directed to the PMB IB Coordinator via email.

8.1.5 <u>Useful Links and Contacts</u>

- CTEP Forms, Templates, Documents: http://ctep.cancer.gov/forms/
- NCI CTEP Investigator Registration: RCRHelpDesk@nih.gov
- PMB policies and guidelines: http://ctep.cancer.gov/branches/pmb/agent_management.htm
- PMB Online Agent Order Processing (OAOP) application: https://ctepcore.nci.nih.gov/OAOP

- CTEP Identity and Access Management (IAM) account: https://ctepcore.nci.nih.gov/iam/
- CTEP IAM account help: ctepreghelp@ctep.nci.nih.gov
- IB Coordinator: IBCoordinator@mail.nih.gov
- PMB email: PMBAfterHours@mail.nih.gov
- PMB phone and hours of service: (240) 276-6575 Monday through Friday between 8:30 am and 4:30 pm (ET)

8.2 Other Investigational Agent(s)

Not Applicable

8.3 Commercial Agent(s)

8.3.1 Paclitaxel

Chemical Name: 5ß,20-Epoxy-1,2α,4,7ß,10ß,13α-hexahydroxytax-11-en-9-one 4,10-diacetate 2-benzoate 13-ester with (2*R*,3*S*)-*N*-benzoyl-3-phenylisoserine.

Classification: taxane, antimicrotubule agent

Molecular Formula: $C_{47}H_{51}NO_{14}$ **Molecular Weight:** 853.9

Product description: Paclitaxel Injection is a clear, colorless to slightly yellow viscous solution. It is supplied as a nonaqueous solution intended for dilution with a suitable parenteral fluid prior to intravenous infusion. Paclitaxel is available in 30 mg (5 mL), 100 mg (16.7 mL), and 300 mg (50 mL) multidose vials. Each mL of sterile nonpyrogenic solution contains 6 mg paclitaxel, 527 mg of purified Cremophor® EL* (polyoxyethylated castor oil) and 49.7% (v/v) dehydrated alcohol, USP.

Solution preparation: Paclitaxel Injection, USP must be diluted prior to infusion. Paclitaxel Injection, USP should be diluted in 0.9% Sodium Chloride Injection, USP, 5% Dextrose Injection, USP, 5% Dextrose and 0.9% Sodium Chloride Injection, USP, or 5% Dextrose in Ringer's Injection to a final concentration of 0.3 to 1.2 mg/mL. Upon preparation, solutions may show haziness, which is attributed to the formulation vehicle. No significant losses in potency have been noted following simulated delivery of the solution through IV tubing containing an in- line (0.22 micron) filter.

Data collected for the presence of the extractable plasticizer DEHP [di-(2ethylhexyl)phthalate] show that levels increase with time and concentration when dilutions are prepared in PVC containers. Consequently, the use of plasticized PVC containers and administration sets is not recommended. Paclitaxel solutions should be prepared and stored in DEHP-free containers, such as glass, polypropylene, or polyolefin containers. Non-PVC containing administration sets, such

as those which are polyethylene-lined, should be used.

Paclitaxel should be administered through an in-line filter with a microporous membrane not greater than 0.22 microns. Use of filter devices such as IVEX-2® filters which incorporate short inlet and outlet PVC-coated tubing has not resulted in significant leaching of DEHP.

The Chemo Dispensing Pin™ device or similar devices with spikes should not be used with vials of TAXOL since they can cause the stopper to collapse resulting in loss of sterile integrity of the TAXOL solution.

Storage and stability: Intact vials are stored at 20° to 25°C (68° to 77°F) and protected from light/retained in carton until time of use. The reconstituted solutions are physically and chemically stable for up to 27 hours at ambient temperature (approximately 25° C) and room lighting conditions.

Route of administration: Short intravenous infusion over approximately 60 minutes

Method of administration: All patients will be pre-medicated prior to paclitaxel administration in order to prevent severe hypersensitivity reactions. The following are suggestions for pre-medication, but investigators should exercise their own judgement and change this as appropriate: dexamethasone 20 mg IV, diphenhydramine (or its equivalent) 50 mg IV, and famotidine 20 mg IV (or its equivalent) administered 30 to 60 minutes before paclitaxel, Paclitaxel will be administered as an IV infusion over approximately 1 hour (+/- 10 minutes) as per institutional guidelines. Body Surface Area (BSA) calculations will be done using the Dubois formula. Dose will be calculated based on BSA on day 1 of every cycle, and dose rounding and dose adjustment due to weight change will be permitted based on institutional guidelines. At a discretion of treating physician, pre-medications may be discontinued if patients develop no hypersensitivity reactions during the first 2 infusions ofpaclitaxel.

Agent Ordering: Paclitaxel is commercially available in 5 mL (30mg), 16.7 mL (100 mg) and 50 mL (300mg) multidose vials. Each ml contains 6 mg of paclitaxel, 527 mg of purified Cremaphor EL® and 50% dehydrated alcohol, USP. Generic form of paclitaxel can be purchased from various vendors.

9 BIOMARKER, CORRELATIVE, AND SPECIAL STUDIES

The focus of this phase I trial will be assessment of safety, tolerability and determination of a recommended phase 2 dose of the study regimen. Given that this trial will enroll a small number of patients, extensive correlative studies will be deferred to a future phase 2 clinical trial. However, we are proposing a limited number of correlatives performed that are outlinedbelow.

Pharmacokinetics

The effect of AT13387 on pharmacokinetics of paclitaxel and the effect of paclitaxel on

pharmacokinetics of AT13387 are not known. Interactions between AT13387 and paclitaxel could result in change in the metabolism of either agent and it is important to investigate them. Pharmacokinetics of both agents given alone and their effects on the metabolism of each other will be evaluated to ensure that there are no clinically significant interactions.

9.1 Integral Laboratory or Imaging Studies

Not Applicable.

9.2 Integrated Correlative Studies

9.2.1 <u>Pharmacokinetics of AT13387 and Paclitaxel</u>

9.2.1.1 Collection of Specimen(s)

Since adequate data to indicate no major drug-drug interactions have already been collected, pharmacokinetics of paclitaxel and AT13387 became optional starting with protocol version 17 dated 05/03/2019. Patients who opt out from pharmacokinetic studies will not need to receive day -7 of AT13387 and can start both AT13387 and paclitaxel on day 1 of cycle 1.

Effect of AT13387 on the pharmacokinetics of paclitaxel as well as the effect of paclitaxel on the pharmacokinetics of AT13387 will be studied. Plasma samples will be collected during Day -7 (Run-in), Cycle 1, Day 1 and Cycle 1, Day 8 to permit PK assessment of AT13387 alone (Day -7), paclitaxel alone (Cycle 1, Day 1) and a combination of AT13387 with paclitaxel (Cycle 1, day 8). The PK assessments of AT13387 and paclitaxel are summarized in the schematicbelow.

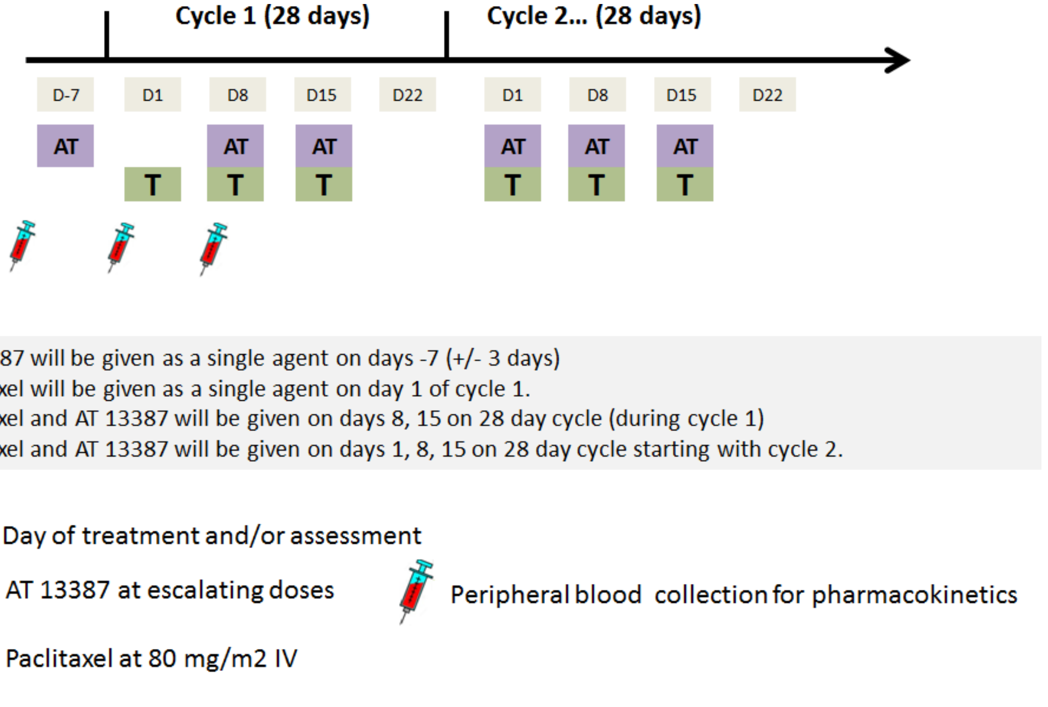

Samples should be collected via a peripheral blood draw in the contralateral arm to the infusion. In the event when a patient has poor venous access and has an existing and functional central venous catheter, sampling through the central line is acceptable. Patients without venous access

will be allowed to have a port placed prior to initiation of study therapy per discretion of the treating physician. Patients without a port will have an option of having a central line inserted during treatment visits, especially on days that require timed blood draws (Day -7, Cycle 1, day 1 and Cycle 1, Day 8). Each sample will be collected into a 6-mL lithium heparin (green top) vacutainer tube. Immediately after collection, the tube is inverted 8-10 times to ensure proper mixing and place tube on ice until centrifugation. The PK specimen will then be centrifuged at 1,200 xg for 10 min at 4 ºC within 1 hour of sample collection. The plasma layer will be transferred into three (3) 2-mL cryovials in roughly equal proportions and frozen immediately at (-70) ºC. An accompany PK requisition form (APPENDIX C: PHARMACOKINETIC (PK) SAMPLES REQUISITION FORM) must be completed for each course of PK sampling. A copy of the completed form must be included with the shipment of the samples for analysis.

The PK of AT13387 will be measured during Day -7 (AT13387 given as a single agent) at following times:

- Pre-dose (within 15 min)
- Immediately prior to end of infusion (EOI) (within 5 min)
- 0.5 hour after end of infusion (±5 min)
- 1 hour after end of infusion (±5 min)
- 2 hours after end of infusion (±5 min)
- 4 hours after end of infusion (±5 min)
- 6 hours after end of infusion (±10 min)
- 8 hour after end of infusion (±10 min)
- 24 hours after end of infusion. (±2 hr)

PK of paclitaxel will be obtained on Cycle 1, Day 1 (paclitaxel given as a single agent) at the following times:

- Pre-dose (within 15 min)
- Immediately prior to end of infusion (EOI) (within 5 min)
- 0.5 hour after end of infusion (±5 min)
- 1 hour after end of infusion (±5 min)
- 2 hours after end of infusion (±5 min)
- 4 hours after end of infusion (±5 min)
- 6 hours after end of infusion (±10 min)
- 8 hour after end of infusion (±10 min)
- 24 hours after end of infusion (±2 hr)

PK of AT13387 and paclitaxel will be obtained on Cycle 1, Day 8 (AT13387 given in combination with paclitaxel) at the following times:

- Pre-dose (within 15 min)

- Immediately prior to end of infusion (EOI) of AT13387 (within 5 min)
- Prior to starting paclitaxel infusion (within 5 min)
- Immediately prior to end of infusion (EOI) of paclitaxel (within 5 min)
- 1 hour after end of paclitaxel infusion (±5 min)
- 2 hours after end of paclitaxel infusion (±5 min)
- 4 hours after end of paclitaxel infusion (±5 min)
- 6 hours after end of paclitaxel infusion (±10 min)
- 24 hours after end of AT13387 infusion (±2 hr)

9.2.1.2 Handling and Shipping of PK Specimen(s)

For study sub-sites, PK samples may be shipped in batches but preferentially no later than 3 months after sample collection. PK samples should be shipped only after all PK samples have been collected for an individual patient. Completed PK samples requisition form must be included in shipment. At the time of shipping, an email notification of the shipment will be sent to **PhASR@osumc.edu**. Samples will then be shipped in batches on dry ice with next-day delivery Monday through Thursday to:

The OSU Pharmacoanalytical Shared Resource Attn: Ming Poi / Jiang Wang
441 Biomedical Research Tower 460 West 12th Avenue
Columbus, Ohio 43210
Phone: (614) 688-0578
Fax: (614) 292-7766
Email: PhASR@osumc.edu

Quantitative liquid chromatography/tandem mass spectrometry (LC/MS/MS) assays for plasma AT13387 and paclitaxel will be developed and validated in the OSUCCC PhASR prior to study accrual and sample analysis.

9.2.1.3 PK comparisons:

To assess the effects of AT13387 on paclitaxel, comparison of paclitaxel PK will be made between Cycle 1, Day 1 (administration of paclitaxel alone) Cycle 1, Day 8 (paclitaxel administered in combination with AT13387). To determine effects of paclitaxel on AT13387, comparison of AT13387 PK on Day -7 (AT13387 administered alone) will be made with Cycle 1, Day 8 (AT13387 administered in combination with paclitaxel).

If drug-drug interaction is occurring between AT13387 and paclitaxel, the PK parameters of AT13387 and/or paclitaxel will alter when comparing the PK parameters between Day -7, C1D1 and C1D8. PK parameters that will be evaluated include but not limited to AUC, CL, $T_{1/2}$, V_z, C_{max}, t_{max}, etc of AT13387 and paclitaxel on those days.

9.2.1.4 Site(s) Performing Correlative Study

Ohio State University Comprehensive Cancer

Center.

9.3 Exploratory/Ancillary Correlative Studies

Any blood samples not utilized for the PK analysis for this specific study will be stored at The Ohio State University. These leftover PK blood samples may be used for any additional future studies and analysis at OSU as new scientific knowledge emerges.

Samples from patients who are currently off study and/or who have expired, can be used for these additional, unknown future studies and analysis. For patients who are currently in follow up, they will be able to re-consent to select whether or not they would like to have their samples used for these additional, unknown future studies.

9.4 Special Studies

Not Applicable

10 STUDY CALENDAR

Baseline evaluations are to be conducted within 2 weeks prior to start of protocol therapy. Scans and x-rays must be done ≤4 weeks prior to the start of therapy. In the event that the patient's condition is deteriorating, laboratory evaluations should be repeated within 48 hours prior to initiation of the next cycle of therapy. All study visits starting with cycle 1, day 1 and beyond have +/- 2 day window, unless otherwise noted.

	Pre-Study[a]	Day -7 (+/- 3)[j]	Day -6 (+/- 3)[j]	C1 D1	C1 D2[j]	C1 D8	C1 D9[j]	C1 D15	C1 D22	C2* D1	C2* D8	C2 * D15	C2* D22	Wk 12	Off Treatment[e] & Follow Up
**AT13387**		A**				A		A		A	A	A			
**Paclitaxel**				B		B		B		B	B	B			
Informed consent[i]	X														
Demographics	X														
Medical history	X														
Concurrent meds	X	X -- X													
Physical exam	X	X		X		X		X	X	X					X
Vital signs[b]	X	X	X[j]	X	X[j]	X	X[j]	X	X	X	X	X			X
Height	X														
Weight	X	X		X		X		X	X	X	X	X			X
Performance status	X	X		X		X		X	X	X					X
CBC w/diff, plts	X	X		X	X[j]	X	X[j]	X	X	X	X	X			X
Serum chemistry[c]	X	X		X		X		X	X	X					X
Urinalysis		X		X						X					
PTT and INR	X	X		X						X					
EKG (as indicated)	X	X		X						X					
Adverse event evaluation		X -- X													X
Tumor measurements	X		Tumor measurements are repeated every 8 weeks (every 2 cycles). Documentation (radiologic) must be provided for patients removed from study for progressive disease.												X
Radiologic evaluation	X		Radiologic measurements should be performed every 8 weeks (every 2 cycles).[h]												X
B-HCG	X[d]									X					
Assessment of Left ventricular Ejection	X														
Pharmacokinetic studies[g]		X	X[j]	X	X[j]	X	X[j]								

	A: AT13387: Dose as assigned; Administered as an IV infusion over approximately 60 minutes (+/- 10 minutes) on Day -7, Cycle 1 day 8, Cycle 1 day 15 and day on day 1, 8 and 15 of the subsequent cycles starting with cycle 2. When given in combination with paclitaxel, AT13387 will be administered first. Patients who opt out from PK analysis, do not need to receive AT13387 on day -7 and will be treated with AT13387 and paclitaxel on day 1 of the first cycle.<br>B: Paclitaxel will be administered at a dose of 80 mg/m2 given as intravenous infusion over approximately 60 minutes (+/- 10 minutes) on days 1, 8 and 15 of 28 day cycles.<br>*: Cycle 2 and all subsequent cycles.<br>**: Patients who opt out from PK analysis, do not need to receive AT13387 on day -7 and will be treated with AT13387 and paclitaxel on day 1 of the first cycle.<br>a: Informed consent, demographics, concurrent medications, physical exam, vital signs, height, weight, Eastern Cooperative Group performance status, complete blood counts, chemistry, PTT and INR, EKG and pregnancy test must be obtained 14 days prior to start of study treatment. Evaluation of left ventricular ejection fraction and tumor measurements must be assessed within 28 days prior to start of study treatment.<br>b: At a minimum, vital signs will be obtained immediately prior to the infusion of AT1337, 15, 30 minutes after the start of infusion and immediately after end or AT13387 infusion. The frequency can increase if clinically indicated. When paclitaxel is administered following administration of AT13387, vital signs will be checked prior to the start of paclitaxel infusion, at 15, 30 minutes after the start of infusion and immediately after the end of paclitaxel infusion. Patient should be monitored in the infusion center for at least 90 minutes following end of infusion of all study therapy. The 90 minute observation period following infusion is not necessary if patients did not have grade 2 or greater reactions in cycle1.<br>c: Albumin, alkaline phosphatase, total bilirubin, bicarbonate, BUN, calcium, chloride, creatinine, glucose, LDH, phosphorus, potassium, total protein, SGOT [AST], SGPT [ALT], sodium.<br>d: Serum pregnancy test (women of child bearing potential).<br>e: Off-treatment evaluation will include all items marked. Follow up after the off-treatment evaluation will continue every 3 months until progression or death, whichever occurs first. Progression and survival data only are needed and may be collected from routine clinic visits or via telephone calls to the patient. No other study assessments are required during follow up. All patients must be off study treatment by May 31st, 2020.<br>f: Transthoracic echocardiogram or Multi-gated Acquisition Scan will be performed at baseline to ensure normal left ventricular ejection fraction<br>g: PK studies for AT 13387 will be collected during day -7, cycle 1, day 8. PK studies of Paclitaxel will be collected during cycle 1, day 1 and cycle 1, day 8. Please see section 9.2.1.1 for additional description of time points. Complete PK requisition form in APPENDIX C: PHARMACOKINETIC (PK) SAMPLES REQUISITION FORM. Patients can opt out from PK analysis as of amendment 17 dated 05/03/19.<br>h: All patients will have baseline assessment of their disease within 4 weeks prior to the first cycle of the study regimen. Subsequently, all study subjects will undergo measurement of response by RECIST 1.1 every 2 cycles of treatment (that is between days 22-28 of cycles prior to which scans are due).Tumor assessments will be done by obtaining a physical examination, Computed Tomography (CT) scan of chest, abdomen and pelvis as well as a Whole Body Nuclear Bone scans. Brain MRI will not be required but can be obtained per discretion of the treating physician. Other imaging scans can be obtained in order to adequately assess response to the study therapy, if clinically indicated based on investigator's opinion.<br>i: Consent to be obtained within the time frame permissible per local policy.<br>j: Does not apply to patients who opt out from the PK studies.

11 MEASUREMENT OF EFFECT

Although the clinical benefit of AT13387 and paclitaxel drug(s) has not yet been established, the intent of offering this treatment is to provide a possible therapeutic benefit, and thus the patient will be carefully monitored for tumor response and symptom relief in addition to safety and tolerability. Patients with measurable disease will be assessed by standard criteria. For the purposes of this study, patients should be re-evaluated every 8 weeks.

11.1 Antitumor Effect – Solid Tumors

For the purposes of this study, patients should be re-evaluated for response every 8 weeks. In addition to a baseline scan, confirmatory scans should also be obtained *4* (not less than 4) weeks following initial documentation of objective response.

Response and progression will be evaluated in this study using the new international criteria proposed by the revised Response Evaluation Criteria in Solid Tumors (RECIST) guideline (version 1.1) [39]. Changes in the largest diameter (unidimensional measurement) of the tumor lesions and the shortest diameter in the case of malignant lymph nodes are used in the RECIST criteria.

11.1.1 Definitions

Evaluable for toxicity. All patients will be evaluable for toxicity from the time of their first treatment with AT13387 and Paclitaxel.

Evaluable for objective response. Only those patients who have measurable disease present at baseline, have received at least one cycle of therapy, and have had their disease re-evaluated will be considered evaluable for response. These patients will have their response classified according to the definitions stated below. (Note: Patients who exhibit objective disease progression prior to the end of cycle 1 will also be considered evaluable.)

Evaluable Non-Target Disease Response. Patients who have lesions present at baseline that are evaluable but do not meet the definitions of measurable disease, have received at least one cycle of therapy, and have had their disease re-evaluated will be considered evaluable for non-target disease. The response assessment is based on the presence, absence, or unequivocal progression of the lesions.

11.1.2 Disease Parameters

Measurable disease. Measurable lesions are defined as those that can be accurately measured in at least one dimension (longest diameter to be recorded) as ≥20 mm (≥2 cm) by chest x-ray or as ≥10 mm (≥1 cm) with CT scan, MRI, or calipers by clinical exam. All tumor measurements must be recorded in millimeters (or decimal fractions of centimeters).

Note: Tumor lesions that are situated in a previously irradiated area might or might not be considered measurable.

Malignant lymph nodes. To be considered pathologically enlarged and measurable, a lymph node must be ≥15 mm (≥1.5 cm) in short axis when assessed by CT scan (CT scan slice thickness recommended to be no greater than 5 mm [0.5 cm]). At baseline and in follow-up, only the short axis will be measured and followed.

Non-measurable disease. All other lesions (or sites of disease), including small lesions (longest diameter <10 mm [<1 cm] or pathological lymph nodes with ≥10 to <15 mm [≥1 to <1.5 cm] short axis), are considered non-measurable disease. Bone lesions, leptomeningeal disease, ascites, pleural/pericardial effusions, lymphangitis cutis/pulmonitis, inflammatory breast disease, and abdominal masses (not followed by CT or MRI), are considered as non-measurable.

Note: Cystic lesions that meet the criteria for radiographically defined simple cysts should not be considered as malignant lesions (neither measurable nor non-measurable) since they are, by definition, simple cysts.

'Cystic lesions' thought to represent cystic metastases can be considered as measurable lesions, if they meet the definition of measurability described above. However, if non-cystic lesions are present in the same patient, these are preferred for selection as target lesions.

Target lesions. All measurable lesions up to a maximum of 2 lesions per organ and 5 lesions in total, representative of all involved organs, should be identified as **target lesions** and recorded and measured at baseline. Target lesions should be selected on the basis of their size (lesions with the longest diameter), be representative of all involved organs, but in addition should be those that lend themselves to reproducible repeated measurements. It may be the case that, on occasion, the largest lesion does not lend itself to reproducible measurement in which circumstance the next largest lesion which can be measured reproducibly should be selected. A sum of the diameters (longest for non-nodal lesions, short axis for nodal lesions) for all target lesions will be calculated and reported as the baseline sum diameters. If lymph nodes are to be included in the sum, then only the short axis is added into the sum. The baseline sum diameters will be used as reference to further characterize any objective tumor regression in the measurable dimension of the disease.

Non-target lesions. All other lesions (or sites of disease) including any measurable lesions over and above the 5 target lesions should be identified as **non-target lesions** and should also be recorded at baseline. Measurements of these lesions are not required, but the presence, absence, or in rare cases unequivocal progression of each should be noted throughout follow-up.

11.1.3 Methods for Evaluation of Measurable Disease

All measurements should be taken and recorded in metric notation using a ruler or calipers. All baseline evaluations should be performed as closely as possible to the beginning of treatment and never more than 4 weeks before the beginning of the treatment.

The same method of assessment and the same technique should be used to characterize each identified and reported lesion at baseline and during follow-up. Imaging-based evaluation is preferred to evaluation by clinical examination unless the lesion(s) being followed cannot be imaged but are assessable by clinical exam.

Clinical lesions Clinical lesions will only be considered measurable when they are superficial (*e.g.*, skin nodules and palpable lymph nodes) and ≥10 mm (≥1 cm) diameter as assessed using calipers (*e.g.*, skin nodules). In the case of skin lesions, documentation by color photography, including a ruler to estimate the size of the lesion, is recommended.

Chest x-ray Lesions on chest x-ray are acceptable as measurable lesions when they are clearly defined and surrounded by aerated lung. However, CT is preferable.

Conventional CT and MRI This guideline has defined measurability of lesions on CT scan based on the assumption that CT slice thickness is 5 mm (0.5 cm) or less. If CT scans have slice thickness greater than 5 mm (0.5 cm), the minimum size for a measurable lesion should be twice the slice thickness. MRI is also acceptable in certain situations (*e.g.* for bodyscans).

Use of MRI remains a complex issue. MRI has excellent contrast, spatial, and temporal resolution; however, there are many image acquisition variables involved in MRI, which greatly impact image quality, lesion conspicuity, and measurement. Furthermore, the availability of MRI is variable globally. As with CT, if an MRI is performed, the technical specifications of the scanning sequences used should be optimized for the evaluation of the type and site of disease. Furthermore, as with CT, the modality used at follow-up should be the same as was used at baseline and the lesions should be measured/assessed on the same pulse sequence. It is beyond the scope of the RECIST guidelines to prescribe specific MRI pulse sequence parameters for all scanners, body parts, and diseases. Ideally, the same type of scanner should be used and the image acquisition protocol should be followed as closely as possible to prior scans. Body scans should be performed with breath-hold scanning techniques, if possible.

PET-CT At present, the low dose or attenuation correction CT portion of a combined PET-CT is not always of optimal diagnostic CT quality for use with RECIST measurements. However, ifthe site can document that the CT performed as part of a PET-CT is of identical diagnostic quality to a diagnostic CT (with IV and oral contrast), then the CT portion of the PET-CT can be used for RECIST measurements and can be used interchangeably with conventional CT in accurately measuring cancer lesions over time. Note, however, that the PET portion of the CT introduces additional data which may bias an investigator if it is not routinely or serially performed. Therefore PET-CT will not be the preferred method of response evaluation in this study. The preferred method of response evaluation in the study will be CT of chest, abdomen and pelvis and a whole body bone scan. However, PET-CT may be used at an investigator's discretion on case by case basis, if the investigator feels that this modality would provide information regarding response to study treatment that would not be otherwise clear with the use of the preferred scans. If PET-CT is needed at baseline to adequately evaluate extend of metastatic disease and monitor a response to the study therapy, it should be a part of baseline evaluation and be used consistently at thetime of subsequent assessments. On the other hand, if the investigator feels that follow up CT scans and

whole body bone scan show findings that are indeterminate for progression which could be resolved with a PET-CT, the study may be added during response evaluation.

Ultrasound Ultrasound is not useful in assessment of lesion size and should not be used as a method of measurement. Ultrasound examinations cannot be reproduced in their entirety for independent review at a later date and, because they are operator dependent, it cannot be guaranteed that the same technique and measurements will be taken from one assessment to the next. If new lesions are identified by ultrasound in the course of the study, confirmation by CT or MRI is advised. If there is concern about radiation exposure at CT, MRI may be used instead of CT in selected instances.

Endoscopy, Laparoscopy The utilization of these techniques for objective tumor evaluation is not advised. However, such techniques may be useful to confirm complete pathological response when biopsies are obtained or to determine relapse in trials where recurrence following complete response (CR) or surgical resection is an endpoint.

Tumor markers Tumor markers alone cannot be used to assess response. If markers are initially above the upper normal limit, they must normalize for a patient to be considered in complete clinical response.

Cytology, Histology These techniques can be used to differentiate between partial responses(PR) and complete responses (CR) in rare cases (*e.g.*, residual lesions in tumor types, suchas germ cell tumors, where known residual benign tumors can remain).

The cytological confirmation of the neoplastic origin of any effusion that appears or worsens during treatment when the measurable tumor has met criteria for response or stable disease is mandatory to differentiate between response or stable disease (an effusion may be a side effect of the treatment) and progressive disease.

FDG-PET While FDG-PET response assessments need additional study, it is sometimes reasonable to incorporate the use of FDG-PET scanning to complement CT scanning in assessment of progression (particularly possible 'new' disease). New lesions on the basis of FDG-PET imaging can be identified according to the following algorithm:

a. Negative FDG-PET at baseline, with a positive FDG-PET at follow-up is a sign of PD based on a new lesion.
b. No FDG-PET at baseline and a positive FDG-PET at follow-up: If the positive FDG-PET at follow-up corresponds to a new site of disease confirmed byCT, this is PD. If the positive FDG-PET at follow-up is not confirmed as a new site of disease on CT, additional follow-up CT scans are needed to determine if there is truly progression occurring at that site (if so, the date of PD will be the date of the initial abnormal FDG-PET scan). If the positive FDG-PET at follow-up corresponds to a pre-existing site of disease on CT that is not progressing on the basis of the anatomic images, this is not PD.
c. FDG-PET may be used to upgrade a response to a CR in a manner similar to a biopsy

in cases where a residual radiographic abnormality is thought to represent fibrosis or scarring. The use of FDG-PET in this circumstance should be prospectively described in the protocol and supported by disease-specific medical literature for the indication. However, it must be acknowledged that both approaches may lead to false positive CR due to limitations of FDG-PET and biopsy resolution/sensitivity.

Note: A 'positive' FDG-PET scan lesion means one which is FDG avid with an uptake greater than twice that of the surrounding tissue on the attenuation corrected image.

11.1.4 Response Criteria

11.1.4.1 Evaluation of Target Lesions

Complete Response (CR): Disappearance of all target lesions. Any pathological lymph nodes (whether target or non-target) must have reduction in short axis to <10 mm (<1 cm).

Partial Response (PR): At least a 30% decrease in the sum of the diameters of target lesions, taking as reference the baseline sum diameters.

Progressive Disease (PD): At least a 20% increase in the sum of the diameters of target lesions, taking as reference the smallest sum on study (this includes the baseline sum if that is the smallest on study). In addition to the relative increase of 20%, the sum must also demonstrate an absolute increase of at least 5 mm (0.5 cm). (Note: the appearance of one or more new lesions is also considered progressions).

Stable Disease (SD): Neither sufficient shrinkage to qualify for PR nor sufficient increase to qualify for PD, taking as reference the smallest sum diameters while on study.

11.1.4.2 Evaluation of Non-Target Lesions

Complete Response (CR): Disappearance of all non-target lesions and normalization of tumor marker level. All lymph nodes must be non-pathological in size (<10 mm [<1 cm] short axis).

Note: If tumor markers are initially above the upper normal limit, they must normalize for a patient to be considered in complete clinical response.

Non-CR/Non-PD: Persistence of one or more non-target lesion(s) and/or maintenance of tumor marker level above the normal limits.

Progressive Disease (PD): Appearance of one or more new lesions and/or *unequivocal progression* of existing non-target lesions. *Unequivocal progression* should not normally trump target lesion status. It must be representative of overall disease status change, not a single lesion increase.

Although a clear progression of "non-target" lesions only is exceptional, the opinion of the treating physician should prevail in such circumstances, and the progression status should beconfirmed at a later time by the review panel (or Principal Investigator).

11.1.4.3 Evaluation of Best Overall Response

The best overall response is the best response recorded from the start of the treatment until disease progression/recurrence (taking as reference for progressive disease the smallest measurements recorded since the treatment started). The patient's best response assignment will depend on the achievement of both measurement and confirmation criteria.

For Patients with Measurable Disease (*i.e.*, Target Disease)

Target Lesions	Non-Target Lesions	New Lesions	Overall Response
CR	CR	No	CR
CR	Non-CR/Non-PD	No	PR
CR	Not evaluated	No	PR
PR	Non-CR/Non-PD/not evaluated	No	PR
SD	Non-CR/Non-PD/not evaluated	No	SD
PD	Any	Yes or No	PD
Any	PD*	Yes or No	PD
Any	Any	Yes	PD

* In exceptional circumstances, unequivocal progression in non-target lesions may be accepted as disease progression.

Note: Patients with a global deterioration of health status requiring discontinuation of treatment without objective evidence of disease progression at that time should be reported as "*symptomatic deterioration.*" Every effort should be made to document the objective progression even after discontinuation of treatment.

For Patients with Non-Measurable Disease (*i.e.*, Non-Target Disease)

Non-Target Lesions	New Lesions	Overall Response
CR	No	CR
Non-CR/non-PD	No	Non-CR/non-PD*
Not all evaluated	No	not evaluated
Unequivocal PD	Yes or No	PD
Any	Yes	PD

* 'Non-CR/non-PD' is preferred over 'stable disease' for non-target disease since SD is increasingly used as an endpoint for assessment of efficacy in some trials so to assign this category when no lesions can be measured is not advised

11.1.5 Duration of Response

Duration of overall response: The duration of overall response is measured from the time measurement criteria are met for CR or PR (whichever is first recorded) until the first date that recurrent or progressive disease is objectively documented (taking as reference for progressive disease the smallest measurements recorded since the treatment started).

The duration of overall CR is measured from the time measurement criteria are first met for CR until the first date that progressive disease is objectively documented.

Duration of stable disease: Stable disease is measured from the start of the treatment until the criteria for progression are met, taking as reference the smallest measurements recorded since the treatment started, including the baseline measurements.

11.1.6 Progression-Free Survival

PFS is defined as the duration of time from start of treatment to time of progression or death, whichever occurs first.

11.1.7 Response Review

Responses will be reviewed by the investigators after completion of the scans and prior to initiation of the next cycle of therapy. Patient files and responses will be reviewed together. Given that response rate is a secondary objective of the trial, independent review will not be conducted.

11.2 Antitumor Effect – Hematologic Tumors

Not Applicable

11.3 Other Response Parameters

Not Applicable

12 STUDY OVERSIGHT AND DATA REPORTING / REGULATORY REQUIREMENTS

Adverse event lists, guidelines, and instructions for AE reporting can be found in Section 7.0 (Adverse Events: List and Reporting Requirements).

12.1 Study Oversight

This protocol is monitored at several levels, as described in this section. The Protocol Principal Investigator is responsible for monitoring the conduct and progress of the clinical trial, including the ongoing review of accrual, patient-specific clinical and laboratory data, and routine and serious adverse events; reporting of expedited adverse events; and accumulation of reported adverse

events from other trials testing the same drug(s). The Protocol Principal Investigator and statistician have access to the data at all times through the CTMS web-based reporting portal.

For the Phase 1 portion of this study, all decisions regarding dose escalation/expansion/de-escalation require sign-off by the Protocol Principal Investigator through the CTMS/IWRS. In addition, for the Phase 1 portion, the Protocol Principal Investigator will have at least monthly, or more frequently, conference calls with the Study Investigators and the CTEP Medical Officer(s) to review accrual, progress, and adverse events and unanticipated problems.

All Study Investigators at participating sites who register/enroll patients on a given protocol are responsible for timely submission of data via Medidata Rave and timely reporting of adverse events for that particular study. This includes timely review of data collected on the electronic CRFs submitted via Medidata Rave.

All studies are also reviewed in accordance with the enrolling institution's data safety monitoring plan.

12.2 Data Reporting

Data collection for this study will be done exclusively through Medidata Rave. Access to the trial in Rave is granted through the iMedidata application to all persons with the appropriate roles assigned in the Regulatory Support System (RSS). To access Rave via iMedidata, the site user must have an active CTEP IAM account (check at https://ctepcore.nci.nih.gov/iam) and the appropriate Rave role (Rave CRA, Rave Read-Only, Rave CRA (Lab Admin), Rave SLA, or Rave Investigator) on either the LPO or participating organization roster at the enrolling site. To the hold Rave CRA role or Rave CRA (Lab Admin), the user must hold a minimum of an AP registration type. To hold the Rave Investigator role, the individual must be registered as an NPIVR or IVR. Associates can hold read-only roles in Rave.

Upon initial site registration approval for the study in RSS, all persons with Rave roles assigned on the appropriate roster will be sent a study invitation e-mail from iMedidata. To accept the invitation, site users must log into the Select Login (https://login.imedidata.com/selectlogin) using their CTEP-IAM user name and password, and click on the "accept" link in the upper right-corner of the iMedidata page. Please note, site users will not be able to access the study in Rave until all required Medidata and study specific trainings are completed. Trainings will be in the form of electronic learnings (eLearnings), and can be accessed by clicking on the link in the upper right pane of the iMedidata screen. Users that have not previously activated their iMedidata/Rave account at the time of initial site registration approval for the study in RSS will also receive a separate invitation from iMedidata to activate their account. Account activation instructions are located on the CTSU website, Rave tab under the Rave resource materials (Medidata Account Activation and Study Invitation Acceptance). Additional information on iMedidata/Rave is available on the CTSU members' website under the Rave tab or by contacting the CTSU Help Desk at 1-888- 823- 5923 or by e-mail at ctsucontact@westat.com.

12.2.1 Method

CTMS Comprehensive Monitoring:
This study will be monitored by the Clinical Trials Monitoring Service (CTMS). Data will be submitted to CTMS at least once every two weeks via Medidata Rave (or other modality if approved by CTEP). Information on CTMS reporting is available at http://www.theradex.com/clinicalTechnologies/?National-Cancer-Institute-NCI-11.
On-site audits will be conducted three times annually (one annual site visit and two data audits). For CTMS monitored studies, after users have activated their accounts, please contact the Theradex Help Desk at (609) 799-7580 or by email at CTMSSupport@theradex.com for additional support with Rave and completion of CRFs.

12.2.2 Responsibility for Data Submission

For ETCTN trials, it is the responsibility of the PI(s) at the site to ensure that all investigators at the ETCTN Sites understand the procedures for data submission for each ETCTN protocol and that protocol specified data are submitted accurately and in a timely manner to the CTMS via the electronic data capture system, Medidata Rave.

Data are to be submitted via Medidata Rave to CTMS on a real-time basis, but no less than once every 2 weeks. The timeliness of data submissions and timeliness in resolving data queries will be tracked by CTMS. Metrics for timeliness will be followed and assessed on a quarterly basis. For the purpose of Institutional Performance Monitoring, data will be considered delinquent if it is greater than 4 weeks past due.

Data from Medidata Rave and CTEP-AERS is reviewed by the CTMS on an ongoing basis as data is received. Queries will be issued by CTMS directly within Rave. The queries will appear on the Task Summary Tab within Rave for the CRA at the ETCTN to resolve. Monthly web-based reports are posted for review by the Drug Monitors in the IDB, CTEP. Onsite audits will be conducted by the CTMS to ensure compliance with regulatory requirements, GCP, and NCI policies and procedures with the overarching goal of ensuring the integrity of data generated from NCI-sponsored clinical trials, as described in the ETCTN Program Guidelines, which may be found on the CTEP (http://ctep.cancer.gov/protocolDevelopment/electronic_applications/adverse_events.htm) and CTSU websites.

An End of Study CRF is to be completed by the PI, and is to include a summary of study endpoints not otherwise captured in the database, such as (for phase 1 trials) include the recommended phase 2 dose (RP2D), and a description of any dose-limiting toxicities (DLTs). CTMS will utilize a core set of eCRFs that are Cancer Data Standards Registry and Repository (caDSR) compliant (http://cbiit.nci.nih.gov/ncip/biomedical-informatics-resources/interoperability-and-semantics/metadata-and-models). Customized eCRFs will be included when appropriate to meet unique study requirements. The PI is encouraged to review the eCRFs, working closely with CTMS to ensure prospectively that all required items are appropriately captured in the eCRFs prior to study activation. CTMS will prepare the eCRFs with built-in edit checks to the extent possible

to promote data integrity.

CDUS data submissions for ETCTN trials activated after March 1, 2014, will be carried out by the CTMS contractor, Theradex. CDUS submissions are performed by Theradex on a monthlybasis. The trial's lead institution is responsible for timely submission to CTMS via Rave, asabove.

Further information on data submission procedures can be found in the ETCTN Program Guidelines (http://ctep.cancer.gov/protocolDevelopment/electronic_applications/adverse_events.htm).

12.3 Data and Safety Monitoring

The data and safety monitoring plan will involve the continuous evaluation of safety, data quality and data timeliness. Investigators will conduct continuous review of data and patient safety at their regular Disease Group meetings (at every 2 weeks) and the discussion will be documented in minutes. For each dose level, the Principal Investigator, study coordinator, and statistician, in consultation with treating physicians as appropriate will review all toxicities at a given dose level to inform the model for dose level adjustments. The Principal Investigator of the trial will review toxicities and responses of the trial where applicable at these disease center meetings and determine if the risk/benefit ratio of the trial changes. Frequency and severity of adverse events will be reviewed by the Principal Investigator and compared to what is known about the agent/device from other sources; including published literature, scientific meetings and discussions with sponsors, to determine if the trial should be terminated before completion.

Serious adverse events will be reviewed by the OSUCCC Data and Safety Monitoring Committee (DSMC). All reportable SAEs will be reported to the IRB of record as per the policies of the IRB.

Safety and trial review teleconferences will be scheduled and moderated by the Multi-Center Trial Program (MCTP). All sites involved in the study should have a representative present for every call to review and discuss patients on study and other applicable agenda items. Teleconferences will be held monthly and may be held more frequently, as needed. For studies closed to accrual with patients expected to remain on long-term treatment and/or follow-up, teleconferences may be extended to occur every two months or quarterly. Decreasing frequency of teleconferences requires OSU PI and MCTP approval.

As of February 2nd, 2020, all sub-site patients are off study. Therefore, no further multi-site teleconferences will be held.

12.4 Collaborative Agreements Language

The agent(s) supplied by CTEP, DCTD, NCI used in this protocol is/are provided to the NCI under a Collaborative Agreement (CRADA, CTA, CSA) between the Pharmaceutical Company(ies) (hereinafter referred to as "Collaborator(s)") and the NCI Division of Cancer Treatment and Diagnosis. Therefore, the following obligations/guidelines, in addition to the provisions in the "Intellectual Property Option to Collaborator" (http://ctep.cancer.gov/industryCollaborations2/intellectual_property.htm) contained within the terms of award, apply to the use of the Agent(s) in this study:

1. Agent(s) may not be used for any purpose outside the scope of this protocol, nor can Agent(s) be transferred or licensed to any party not participating in the clinical study. Collaborator(s) data for Agent(s) are confidential and proprietary to Collaborator(s) and shall be maintained as such by the investigators. The protocol documents for studies utilizing Agents contain confidential information and should not be shared or distributed without the permission of the NCI. If a copy of this protocol is requested by a patient or patient's family member participating on the study, the individual should sign a confidentiality agreement. A suitable model agreement can be downloaded from: http://ctep.cancer.gov.

2. For a clinical protocol where there is an investigational Agent used in combination with (an)other Agent(s), each the subject of different Collaborative Agreements, the access toand use of data by each Collaborator shall be as follows (data pertaining to such combinationuse shall hereinafter be referred to as "Multi-Party Data"):

   a. NCI will provide all Collaborators with prior written notice regarding the existence and nature of any agreements governing their collaboration with NCI, the design of the proposed combination protocol, and the existence of any obligations that would tend to restrict NCI's participation in the proposed combination protocol.

   b. Each Collaborator shall agree to permit use of the Multi-Party Data from the clinical trial by any other Collaborator solely to the extent necessary to allow said other Collaborator to develop, obtain regulatory approval or commercialize its own Agent.

   c. Any Collaborator having the right to use the Multi-Party Data from these trials must agree in writing prior to the commencement of the trials that it will use the Multi-Party Data solely for development, regulatory approval, and commercialization of its ownAgent.

3. Clinical Trial Data and Results and Raw Data developed under a Collaborative Agreement will be made available to Collaborator(s), the NCI, and the FDA, as appropriate and unless additional disclosure is required by law or court order as described in the IP Option to Collaborator (http://ctep.cancer.gov/industryCollaborations2/intellectual_property.htm). Additionally, all Clinical Data and Results and Raw Data will be collected, used and disclosed consistent with all applicable federal statutes and regulations for the protection of human subjects, including, if applicable, the *Standards for Privacy of Individually Identifiable Health Information* set forth in 45 C.F.R. Part 164.

4. When a Collaborator wishes to initiate a data request, the request should first be sent to the NCI, who will then notify the appropriate investigators (Group Chair for Cooperative Group studies, or PI for other studies) of Collaborator's wish to contact them.

5. Any data provided to Collaborator(s) for Phase 3 studies must be in accordance with the guidelines and policies of the responsible Data Monitoring Committee (DMC), if there is a DMC for this clinicaltrial.

6. Any manuscripts reporting the results of this clinical trial must be provided to CTEP by the

Group office for Cooperative Group studies or by the Principal Investigator for non-Cooperative Group studies for immediate delivery to Collaborator(s) for advisory review and comment prior to submission for publication. Collaborator(s) will have 30 days from the date of receipt for review. Collaborator shall have the right to request that publication be delayed for up to an additional 30 days in order to ensure that Collaborator's confidential and proprietary data, in addition to Collaborator(s)'s intellectual property rights, are protected. Copies of abstracts must be provided to CTEP for forwarding to Collaborator(s) for courtesy review as soon as possible and preferably at least three (3) days prior to submission, but in any case, prior to presentation at the meeting or publication in the proceedings. Press releases and other media presentations must also be forwarded to CTEP prior to release. Copies of any manuscript, abstract and/or press release/ media presentation should be sent to:

Email: ncicteppubs@mail.nih.gov

The Regulatory Affairs Branch will then distribute them to Collaborator(s). No publication, manuscript or other form of public disclosure shall contain any of Collaborator's confidential/ proprietary information.

13. STATISTICAL CONSIDERATIONS

13.1 Study Design/Endpoints

This is a standard 3+3 phase I trial design in women or men with unresectable, locally advanced or metastatic triple negative breast cancer who received any number of prior lines of chemotherapy to establish the recommended phase 2 dose, evaluate safety profile, explore biomarkers of response and toxicity and preliminarily evaluate the objective response rates to the study combination of AT13387 and paclitaxel.

The primary objectives of the study are to determine the recommended phase 2 dose (RP2D) and to determine the toxicity Profile (based on CTCAE v. 5.0) of AT13387 in combination with paclitaxel in patients with advanced triple negative breast cancer (TNBC). Subjects who complete cycle 1 of therapy or those who have received at least 2 of the 3 doses of taxol and AT13387 in cycle 1 will comprise the DLT population. Dose modifications or interruptions of AT13387 and Paclitaxel will not be allowed during the DLT period for patients in dose escalation cohort unless a patient experiences 1 or more dose limiting toxicities; or if the patient developstoxicities that require dose interruption based on dose modification tables in section 6, in which case either or both of the study medications may be held. If either study medication is held, all missed doses will not be made up upon resuming study therapy. Patients who do not complete the DLT period for reasons other than toxicities will be replaced. Grading of toxicities will be assessed by the use of Common Terminology Criteria for Adverse Events (CTCAE) version 5.0 with details listed in section 7 of the treatment plan. The RP2D is defined as (1) the dose level at which no more than 1 of 6 patients experiences a DLT (maximum tolerated dose or MTD); or (2) doses of the combination below MTD, if in the opinion of the investigators, lower doses are better tolerated and safer. The first criterion will be given the priority when determining RP2D. The second criterion will only apply if an unexpected and highly clinically significant toxicity is noted that does not neatly fit the DLT criteria (for example, >1 patients in a given dose level develop

grade 4 thrombocytopenia after cycle 2). The dose escalation rules follow the standard design (summarized in the table 5.3), where cohorts of 3 patients are accrued to a dose level and evaluated for DLT during the DLT observation period before making a decision to dose escalate or not. Ifin the first 3 patients treated at a dose level there are no DLT's observed, the next cohort of 3 can be accrued to the next dose level. If one or more of the first 3 patients at a dose level experiences DLT, then 3 additional patients will be accrued to that dose level to ensure it is tolerable and does not exceed the MTD. Any dose that has two or more patients with DLTs has exceeded the MTD and is deemed to have unacceptable toxicity. Three additional patients will be entered at the next lowest dose level if only 3 patients were treated previously at that dose. If the first dose level proves to have unacceptable toxicity (i.e. 2 or more DLTs) then 3-6 patients will be accrued to the de-escalation dose (i.e. dose level (-1)). The MTD will be determined as the highest dose level at which there is 1 or fewer DLTs in 6 patients. At least 6 patients will be enrolled at MTD. For the purposes of this study, the DLT observation period will be defined as the first cycle of therapy; any DLTs observed during this time will be used to direct dose escalation decisions.

13.2 Sample Size/Accrual Rate

This proposal is for a multi-institutional phase I study. Four to five (including dose level -1) cohorts of 3-15 patients each, for a maximum of 6-33 patients will be evaluated at 4-5 different dose levels. In order to obtain more data about safety/tolerability and pharmacokinetics, a total of 15 patients will be enrolled to the recommended phase 2 dose (RP2D). Once the MTD has been reached, it will be expanded to a total of 15 patients to (1) further characterize the safety and tolerability of the combination; (2) provide additional data on effect of either agent on PK of the other agent and; (3) provide estimates for preliminary anti-tumor activity with more precision levels. A sample size of 12 patients (with 3 additional patients to account for early drop out or unevaluable patients) treated at the RP2D is sufficient to detect a DLT in at least one patient with a high probability. The following table shows the probabilities for observing a DLT in at least one of the 12 patients treated at the MTD.

True DLT rate	10%	15%	20%	30%	35%	40%
Probability of detecting at DLT in at least one patient (out of 12)	0.72	0.86	0.93	0.97	0.99	>0.99

Based on the above table, if the true DLT rate is between 10-35%, then the probability that a DLT will be detected in at least one patient ranges from 72-99%.

Furthermore, a sample size of 12 patients improve accuracy in estimating preliminary anti-tumor activity levels at the RP2D by an estimated standard error of 15% and the length of 95% confidence interval to be no wider than 50%. This more accurate estimate of will be useful in designing a subsequent phase II study. We will enroll 15 patients to account for 2-3 patients with early drop out (unevaluable).

The accrual rate is expected to be approximately 2 patients per month across participating sites.

The estimated time to study completion will be 2 years or less.

PLANNED ENROLLMENT REPORT

Racial Categories	Ethnic Categories				Total
	Not Hispanic or Latino		Hispanic or Latino		
	Female	Male	Female	Male	
American Indian/ Alaska Native	0-1*	0	0	0	**0-1**
Asian	0-2	0	0	0	**0-1**
Native Hawaiian or Other Pacific Islander	0-1	0	0	0	**0-1**
Black or African American	1-4	0	0	0	**1-4**
White	4-17	0	0-2	0	**4-21**
More Than One Race	1-2	0	0-3	0	**1-5**
**Total**	**6-27**	**0**	**0-4**	**0**	**6-33**

PHS 398 / PHS 2590 (Rev. 08/12 Approved Through 8/31/2015) OMB No. 0925-0001/0002

*Depends on the total number of study subjects.

13.3 Stratification Factors

Not Applicable.

13.4 Analysis of Secondary Endpoints

Secondary Objectives are to determine the effect of AT13387 on pharmacokinetics of paclitaxel in the study population by measuring PK of AT13387 during day -7 and cycle 1, day 8; to determine the effect of paclitaxel on pharmacokinetics of AT13387 in the study population by measuring PKs of paclitaxel during cycle 1, day 1 and cycle 1, day 8; and to determine the overall response rate (partial response + complete response), response duration and progression-free survival in the study patients.

Pharmacokinetics (PK)

We will assess the PK parameters for AT13387 alone during day -7 (+/- 3 days), for paclitaxel alone during cycle 1, day 1 and the combination of the two in cycle 1, day 8 for all patients in the study. A descriptive analysis will be performed to define systemic exposure, drug clearance, and other pharmacokinetic parameters. The PK parameters will be summarized with simple summary

statistics, including means, medians, ranges, and standard deviations (if numbers and distribution permit).

Overall Response Rate

Overall response will also be assessed in all patients in an exploratory manner, using summary statistics, by dose level. Response will be defined using the best response achieved in the first 6 months and will include CR or PR based on RECIST 1.1 criteria. The overall response rate will be calculated as the proportion of patients who achieve a CR or PR divided by the total number of patients who have received at least one dose of therapy per protocol. We will also calculate corresponding 95% binomial confidence intervals for these response rates.

Progression Free Survival

For descriptive purposes only, we will also summarize the progression-free survival. Progression free survival will be defined as the interval from study enrollment to first documented disease progression according to RECIST 1.1 or death from any cause (whichever occurs first). Progression free survival will be summarized using Kaplan and Meier methods, where patients who are event-free at the time of their last evaluation will be censored at that time point.

14 REFERENCES

1. Bauer KR, Brown, Cress M, Parise RD. Descriptive analysis of estrogen receptor (ER)-negative, progesterone receptor (PR)-negative, and HER2-negative invasive breast cancer, the so-called triple negative phenotype: a population-based study from the California Cancer Registry. Cancer 2007;109:1721–28.

2. Carey L, Winer E, Viale G, Cameron D, Gianni L. Triple-negative breast cancer: disease entity or title of convenience? Nat Rev Clin Oncol. 2010;7:683–92.

3. Normant E, Paez G, West KA, et al. The HSP90 inhibitor IPI-504 rapidly lowers EML4-ALK levels and induces tumor regression in ALK-driven NSCLC models. Oncogene 2011;30:2581-2586.

4. Pearl LH, Prodromou C, Workman P. The Hsp90 molecular chaperone: an open and shut case for treatment Biochem J. 2008;410:439-53.

5. Shimamura T, Lowell A, Engelman J, et al. Epidermal growth factor receptors harboring kinase domain mutations associate with the heat shock protein 90 chaperone and are destabilized following exposure to geldanamycins. Cancer Res. 2005;65:6401-8.

6. Xu W, Marcu M, Yuan X, et al. Chaperone-dependent E3 ubiquitin ligase CHIP mediates a degradative pathway for c ErbB2/Neu. PNAS 2002;99:12847–52.

7. Workman P, Burrows F, Neckers L. Drugging the cancer chaperone HSP90: combinatorial therapeutic exploitation of oncogene addiction and tumor stress. Ann N Y Acad Sci. 2007;1113:202-16.

8. Kamal A, Thao L, Sensintaffar J, et al. A high-affinity conformation of HSP90 confers tumor selectivity on HSP90 inhibitors Nature 2003;425:407-10.

9. Pick E, Kluger Y, Giltnane J, et al. High HSP90 expression is associated with decreased survival in breast cancer. Cancer Res. 2007;67:2392-7.

10. Graham B, Curry, J, Smyth T, et al. The heat shock protein 90 inhibitor, AT13387, displays a long duration of action in vitro and in vivo in non-small cell lung cancer. Cancer Sci. 2012;103:522-7.

11. Smyth T, Van Looy T, Curry JE, et al. The HSP90 inhibitor AT13387 is effective against imatinib-sensitive and –resistant gastrointestinal stromal tumor models. Mol Cancer Ther. 2012;11:1799-808.

12. Hong DS, Banerji U, Tavana B, et al. Targeting the molecular chaperone heat shock protein 90 (HSP90): Lessons learned and future directions. Cancer Treat Rev. 2012;39:375-87.

13. Jhaveri K, Taldone, T, Modi S, et al Advances in the clinical development of heat shock protein 90 (Hsp90) inhibitors in cancers Biochim Biophys Acta. 2012;1823:742-55.

14. Citri A, Harari D, Shohat G, et al. Hsp90 recognizes a common surface on client kinases.J Biol Chem. 2006;281:14361-9.

15. Al-Ejeh F, Miranda M, Shi W, et al. Kinome profiling reveals breast cancer heterogeneity and identifies targeted therapeutic opportunities for triple negative breast cancer. Oncotarget 2014;5:3145-58.

16. Caldas-Lopes E, Cerchietti L, Ahn JH, et al. Hsp90 inhibitor PU-H71, a multimodal inhibitor of malignancy, induces complete responses in triple-negative breast cancer models. Proc Natl Acad Sci U S A. 2009;106:8368-73.

17. Seidman AD, Berry D, Cirrincione C, et al. Randomized phase III trial of weekly compared with every-3-weeks paclitaxel for metastatic breast cancer, with trastuzumab for all HER-2 overexpressors and random assignment to trastuzumab or not in HER-2 nonoverexpressors: final results of Cancer and Leukemia Group B protocol 9840. J Clin Oncol. 2008;26:1642-9.

18. Clark AS, West K, Streicher S, Dennis PA. Constitutive and Inducible Akt Activity Promotes Resistance to Chemotherapy, Trastuzumab, or Tamoxifen in Breast Cancer Cells. Mol Cancer Ther. 2002;1:707-717.

19. Solit DB, Basso AD, Olshen AB, Scher HI, Rosen N. Inhibition of heat shock protein 90 function down-regulates Akt kinase and sensitizes tumors to Taxol. Cancer Res. 2003;63:2139-44.

20. Proia DA, Zhang C, Sequeira M, et al. Preclinical activity profile and therapeutic efficacy of the HSP90 inhibitor ganetespib in triple-negative breast cancer. Clin Cancer Res. 2014;20:413-24.

21. Woodhead AJ, Angove H, Carr MG, et al. Discovery of (2,4-dihydroxy-5-isopropylphenyl)-[5-(4-methylpiperazin-1-ylmethyl)-1,3-dihydroisoindol-2-yl]methanone (AT13387), a novel inhibitor of the molecular chaperone Hsp90 by fragment based drug design. J Med Chem. 2010;53:5956-69.

22. Lyons J, Graham B, Reule M, et al. AT13387, A Fragment-Derived Clinical Candidate is Active in Lung Cancer and Melanoma Models. The 20th EORTC-NCI-AACR Symposium 2008;147 (Poster).

23. Seidman A, Tiersten A, Hudis C, et al. Phase II trial of paclitaxel by 3-hour infusion as initial and salvage chemotherapy for metastatic breast cancer. J Clin Oncol. 1995;

13:2575–81.

24. Hudis C, Seidman A, Crown JPA, et al. Phase II and pharmacologic study of docetaxel as initial chemotherapy for metastatic breast cancer. J Clin Oncol. 1996;14:58–65.

25. Gradishar W, Tjulandin S, Davidson N, et al. Phase III trial of nanoparticle albumin-bound paclitaxel compared with polyethylated castor oil-based paclitaxel in women with breast cancer. J Clin Oncol. 2005; 23:7794–7803.

26. Wani MC, Taylor HL, Wall ME, Coggon P, Mcphail AT. Plant antitumor agents. VI. The isolation and structure of taxol, a novel antileukemic and antitumor agent from Taxus brevifolia. J Am Chem Soc. 1971;93:2325-7.

27. Horwitz SB. Personal recollections on the earlydevelopment of taxol. J Nat Prod. 2004;317 67;136-8.

28. Schiff PB, Fant J, Horwitz SB. Promotion of microtubule assembly in vitro by taxol. Nature 1979;277:665-7.

29. Rowinsky EK, Donehower RC. Paclitaxel (taxol). N Engl J Med. 1995;332:1004-14.

30. Berger MJ, Vargo C, Vincent M, et al. Stopping paclitaxel premedication after twodoses in patients not experiencing a previous infusion hypersensitivity reaction. Support Care Cancer. 2015;23:2019-24.

31. Sledge GW, Neuberg D, Bernardo P, et al. Phase III trial of doxorubicin, paclitaxel, and the combination of doxorubicin and paclitaxel as front-line chemotherapy for metastatic breast cancer: an intergroup trial (E1193). J Clin Oncol. 2003;21:588-92.

32. Norton L. Theoretical concepts and the emerging role of taxanes in adjuvant therapy. Oncologist 2001;6:30-35.

33. Symmans WF, Volm MD, Shapiro RL, et al. Paclitaxel-induced apoptosis and mitotic arrest assessed by serial fine-needle aspiration: implications for early prediction of breast cancer response to neoadjuvant chemotherapy. Clin Cancer Res. 2000;6:4610-17.

34. Green MC, Buzdar AU, Smith T et al. Weekly paclitaxel improves pathologic complete remission in operable breast cancer when compared with paclitaxel once every 3 weeks. J Clin Oncol.2005;23:5983-92.

35. Sparano JA, Wang M, Martino S, et al. Weekly paclitaxel in adjuvant treatment of breast cancer. N Engl J Med. 2008;358:1663-71.

36. Sikov WM, Berry DA, Perou CM, et al. Impact of the addition of carboplatin and/or bevacizumab to neoadjuvant once-per-week paclitaxel followed by dose-dense doxorubicin and cyclophosphamide on pathologic complete response rates in stage II to III triple-negative breast cancer: CALGB 40603 (Alliance). J Clin Oncol. 2015;33:13-21.

37. Romond EH, Perez EA, Bryant J et al. Trastuzumab plus Adjuvant Chemotherapy for Operable HER2-Positive Breast Cancer. N Engl J Med. 2005;353:1673-84.

38. Baselga J, Bradbury I, Eidtmann H, et al. Lapatinib with trastuzumab for HER2-positive early breast cancer (NeoALTTO): a randomised, open-label, multicentre, phase 3 trial. Lancet. 2012;379:633-40.

39. Eisenhauer EA, Therasse P, Bogaerts J, et al. New response evaluation criteria in solid tumours: revised RECIST guideline (version 1.1). Eur J Cancer. 2009;45:228-47.

APPENDIX A: PERFORMANCE STATUS CRITERIA

<table>
<tr><th colspan="2">ECOG Performance Status Scale</th><th colspan="2">Karnofsky Performance Scale</th></tr>
<tr><th>Grade</th><th>Descriptions</th><th>Percent</th><th>Description</th></tr>
<tr><td rowspan="2">0</td><td rowspan="2">Normal activity. Fully active, able to carry on all pre-disease performance without restriction.</td><td>100</td><td>Normal, no complaints, no evidence of disease.</td></tr>
<tr><td>90</td><td>Able to carry on normal activity; minor signs or symptoms of disease.</td></tr>
<tr><td rowspan="2">1</td><td rowspan="2">Symptoms, but ambulatory. Restricted in physically strenuous activity, but ambulatory and able to carry out work of a light or sedentary nature (e.g., light housework, office work).</td><td>80</td><td>Normal activity with effort; some signs or symptoms of disease.</td></tr>
<tr><td>70</td><td>Cares for self, unable to carry on normal activity or to do active work.</td></tr>
<tr><td rowspan="2">2</td><td rowspan="2">In bed <50% of the time. Ambulatory and capable of all self-care, but unable to carry out any work activities. Up and about more than 50% of waking hours.</td><td>60</td><td>Requires occasional assistance, but is able to care for most of his/her needs.</td></tr>
<tr><td>50</td><td>Requires considerable assistance and frequent medical care.</td></tr>
<tr><td rowspan="2">3</td><td rowspan="2">In bed >50% of the time. Capable of only limited self-care, confined to bed or chair more than 50% of waking hours.</td><td>40</td><td>Disabled, requires special care and assistance.</td></tr>
<tr><td>30</td><td>Severely disabled, hospitalization indicated. Death not imminent.</td></tr>
<tr><td rowspan="2">4</td><td rowspan="2">100% bedridden. Completely disabled. Cannot carry on any self-care. Totally confined to bed or chair.</td><td>20</td><td>Very sick, hospitalization indicated. Death not imminent.</td></tr>
<tr><td>10</td><td>Moribund, fatal processes progressing rapidly.</td></tr>
<tr><td>5</td><td>Dead.</td><td>0</td><td>Dead.</td></tr>
</table>

APPENDIX B: INFORMATION ON POSSIBLE DRUG INTERACTIONS

Information on Possible Interactions with Other Agents for Patients and Their Caregivers and Non-Study Healthcare Team

Paclitaxel interacts with many drugs that are processed by your liver. Because of this, it is very important to tell your study doctors about all of your medicine before you start this study. It is also very important to tell them if you stop taking any regular medicine, or if you start taking a new medicine while you take part in this study. When you talk about your medicine with your study doctor, include medicine you buy without a prescription at the drug store (over-the-counter remedy), or herbal supplements such as St. John's wort.

Many health care prescribers can write prescriptions. You must also tell your other prescribers (doctors, physicians' assistants or nurse practitioners) that you are taking part in a clinical trial. **Bring this paper with you and keep the attached information card in your wallet**. These are the things that you and they need to know:

Paclitaxel interacts with (a) certain specific enzyme(s) in your liver.

- The enzyme(s) in question is/are **CYP 2C8 and CYP 3A4.** One of the therapeutic agents in the study is paclitaxel which is broken down by the above enzymes in order to be cleared from your system.
- Paclitaxel must be used very carefully with other medicines that need these liver enzymes to be effective or to be cleared from your system.
- Other medicines may also affect the activity of the enzyme.
- Substances that increase the enzyme's activity ("inducers") could reduce the effectiveness of the drug, while substances that decrease the enzyme's activity ("inhibitors") could result in high levels of the active drug, increasing the chance of harmful side effects.
- You and healthcare providers who prescribe drugs for you must be careful about adding or removing any drug in this category.
- Before you start the study, your study doctor will work with your regular prescriber to switch any medicines that are considered strong inducers/inhibitors or substrates of **CYP 2C8 and CYP 3A4**.
- Please be very careful! Over-the-counter drugs have a brand name on the label—it's usually big and catches your eye. They also have a generic name—it's usually small and located above or below the brand name, and printed in the ingredient list. Find the generic name and determine, with the pharmacist's help, whether there could be an adverse interaction.
- Be careful:
    - If you take acetaminophen regularly: You should not take more than 4 grams a day [no more than: 12 tablets of regular tylenol (325 mg), 8 tablets of Tylenol Extra Strength (500 mg) and 6 tablets of Tylenol Arthritis (625 mg)] if you are an adult or 2.4 grams a day [no more than: 7 tablets of regular tylenol (325 mg), 4 tablets of Tylenol Extra Strength (500 mg) and 3 tablets of Tylenol Arthritis (625 mg)] if you are older than 65 years of age. Read labels carefully! Acetaminophen

is an ingredient in many medicines for pain, flu, and cold.

- If you drink grapefruit juice or eat grapefruit: Avoid these until the study is over.
- If you take herbal medicine regularly: You should not take St. John's wort while you are taking Paclitaxel.

Other medicines can be a problem with your study drugs.

- You should check with your doctor or pharmacist whenever you need to use an over-the-counter medicine or herbal supplement.
- Your regular prescriber should check a medical reference or call your study doctor before prescribing any new medicine for you. Your study doctor's name is____ and he or she can be contacted at:

**INFORMATION ON POSSIBLE DRUG INTERACTIONS**<br>You are enrolled on a clinical trial using the experimental agent ________________**.** This clinical trial is sponsored by the NCI. ________________interacts with drugs that are processed by your liver. Because of this, it is very important to:<br>➢ Tell your doctors if you stop taking regular medicine or if you start taking a new medicine.<br>➢ Tell all of your prescribers (doctor, physicians' assistant, nurse practitioner, pharmacist) that you are taking part in a clinical trial.<br>➢ Check with your doctor or pharmacist whenever you need to use an over-the-counter medicine or herbal supplement.	________________interacts with a specific liver enzyme called **CYP**______, and must be used very carefully with other medicines that interact with this enzyme.<br>➢ Before you start the study, your study doctor will work with your regular prescriber to switch any medicines that are considered "strong inducers/inhibitors or substrates of **CYP_2C8 and 3A4**."<br>➢ Before prescribing new medicines, your regular prescriber should go to a frequently-updated medical reference for a list of drugs to avoid, or contact your study doctor.<br>➢ Your study doctor's name is ____________________________<br>and can be contacted at______________________________**.**

APPENDIX C: PHARMACOKINETIC (PK) SAMPLES REQUISITION FORM

Site Number: _____________

Subsite Principal Investigator: ____________________________________

Subject ID:______________ Subject Initials:__________(first, middle, last)

Collection Cycle and Day: ☐ Run-in, D(-7) ☐ C1D1 ☐ C1D8

Date of Collection:___/_____/___________(Day/Mo/Year)

Complete the applicabletable:

D(-7) or C1D1 **For D(-7): AT13387 dose:__________mg**
For C1D1: paclitaxel dose:__________mg

**Time Point/Description**	**Time of collection (military time; hr:min)**
Pre-dose (within 15 min)	____:____
Start of infusion	____:____
Immediately PRIOR to end of infusion (EOI) (within 5 min)	____:____
0.5 hour after end of infusion (±5 min)	____:____
1 hour after end of infusion (±5 min)	____:____
2 hour after end of infusion (±5 min)	____:____
4 hour after end of infusion (±5 min)	____:____
6 hour after end of infusion (±10 min)	____:____
8 hour after end of infusion (±10 min)	____:____
24 hour after end of infusion (±2 hr)	____:____

C1D8 **AT13387 dose:_______mg; paclitaxel dose:_______mg**

**Time Point/Description**	**Time of collection (military time; hr:min)**
Pre-dose of AT13387 (within 15 min)	____:____
Start of AT 13387 infusion	____:____
Immediately PRIOR to end of infusion (EOI) of AT13387 (within 5 min)	____:____
Pre-dose of paclitaxel (within 5 min)	____:____
Start of paclitaxel infusion	____:____
Immediately PRIOR to end of infusion (EOI) of paclitaxel (within 5 min)	____:____
1 hour after end of paclitaxel infusion (±5 min)	____:____
2 hour after end of paclitaxel infusion (±5 min)	____:____
4 hour after end of paclitaxel infusion (±5 min)	____:____
6 hour after end of paclitaxel infusion (±10 min)	____:____
24 hour after end of paclitaxel infusion (±2 hr)	____:____

APPENDIX D: LIST OF CLINICALLY SIGNIFICANT OR STRONG CYP 3A4 AND CYP2C8 INHIBITORS AND INDUCERS

	**Inducers**	**Inhibitors**
**CYP3A4**	bosentan, carbamazepine, efavirenz, nevirapine, oxcarbazepine, phenytoin, phenobarbital, rifampin, St. John's wort	boceprevir, clarithromycin, conivaptan, indinavir, itraconazole, ketoconazole, lopinavir/ritonavir, nefazodone, nelfinavir, posaconazole, ritonavir, saquinavir, telaprevir, telithromycin, voriconazole
**CYP2C8**	rifampin	gemfibrozil

Please note: This is not an exhaustive list and it has been abbreviated to include only clinically relevant inhibitors and inducers of CYP 3A4 and CYP2C8.

Reference:

1. PL Detail-Document, Cytochrome P450 Drug Interactions. Pharmacist's Letter/Prescriber's Letter. October 2013.
2. P450 Drug Interaction Table: Abbreviated "Clinically Relevant" Table. <http://medicine.iupui.edu/clinpharm/ddis/clinical-table>

Supplementary Table 1: CONSORT 2025 checklist of information to include when reporting a randomised trial*

Section / Topic	No	CONSORT 2025 checklist item description	Reported on page no.
**Title and abstract**			
Title and structured abstract	1a	Identification as a randomised trial	N/A, not randomized
	1b	Structured summary of the trial design, methods, results, and conclusions	2, 4
**Open science**			
Trial registration	2	Name of trial registry, identifying number (with URL) and date of registration	2, 4, 9
Protocol and statistical analysis plan	3	Where the trial protocol and statistical analysis plan can be accessed	11, Supp Fig 3
Data sharing	4	Where and how the individual de-identified participant data (including data dictionary), statistical code and any other materials can be accessed	11, Supp Fig 3
Funding and conflicts of interest	5a	Sources of funding and other support (e.g., supply of drugs), and role of funders in the design, conduct, analysis and reporting of the trial	11-12
	5b	Financial and other conflicts of interest of the manuscript authors	12
**Introduction**			
Background and rationale	6	Scientific background and rationale	3-4
Objectives	7	Specific objectives related to benefits and harms	3-4
**Methods**			
Patient and public involvement	8	Details of patient or public involvement in the design, conduct and reporting of the trial	N/A
Trial design	9	Description of trial design including type of trial (e.g., parallel group, crossover), allocation ratio, and framework (e.g., superiority, equivalence, non-inferiority, exploratory)	9-10
Changes to trial protocol	10	Important changes to the trial after it commenced including any outcomes or analyses that were not prespecified, with reason	N/A
Trial setting	11	Settings (e.g., community, hospital) and locations (e.g., countries, sites) where the trial was conducted	Supp Fig 3
Eligibility criteria	12a	Eligibility criteria for participants	9-10, Supp Fig 3

	12b	If applicable, eligibility criteria for sites and for individuals delivering the interventions (e.g., surgeons, physiotherapists)	N/A
Intervention and comparator	13	Intervention and comparator with sufficient details to allow replication. If relevant, where additional materials describing the intervention and comparator (e.g., intervention manual) can be accessed	N/A
Outcomes	14	Pre-specified primary and secondary outcomes, including the specific measurement variable (e.g., systolic blood pressure), analysis metric (e.g., change from baseline, final value, time to event), method of aggregation (e.g., median, proportion), and time point for each outcome	6, Supp Fig 3
Harms	15	How harms were defined and assessed (e.g., systematically, non-systematically)	10
Sample size	16a	How sample size was determined, including all assumptions supporting the sample size calculation	11
	16b	Explanation of any interim analyses and stopping guidelines	Supp Fig 3
Randomisation:			
Sequence generation	17a	Who generated the random allocation sequence and the method used	N/A
	17b	Type of randomisation and details of any restriction (e.g., stratification, blocking and block size)	N/A
Allocation concealment mechanism	18	Mechanism used to implement the random allocation sequence (e.g., central computer/telephone; sequentially numbered, opaque, sealed containers), describing any steps to conceal the sequence until interventions were assigned	N/A
Implementation	19	Whether the personnel who enrolled and those who assigned participants to the interventions had access to the random allocation sequence	N/A
Blinding	20a	Who was blinded after assignment to interventions (e.g., participants, care providers, outcome assessors, data analysts)	N/A
	20b	If blinded, how blinding was achieved and description of the similarity of interventions	N/A
Statistical methods	21a	Statistical methods used to compare groups for primary and secondary outcomes, including harms	10
	21b	Definition of who is included in each analysis (e.g., all randomised participants), and in which group	9
	21c	How missing data were handled in the analysis	9, Supp Fig 3
	21d	Methods for any additional analyses (e.g., subgroup and sensitivity analyses), distinguishing prespecified from post-hoc	N/A
**Results**			
Participant flow, including flow diagram	22a	For each group, the numbers of participants who were randomly assigned, received intended intervention, and were analysed for the primary outcome	9
	22b	For each group, losses and exclusions after randomisation, together with reasons	9
Recruitment	23a	Dates defining the periods of recruitment and follow-up for outcomes of benefits and harms	9
	23b	If relevant, why the trial ended or was stopped	N/A

Intervention and comparator delivery	24a	Intervention and comparator as they were actually administered (e.g., where appropriate, who delivered the intervention/comparator, how participants adhered, whether they were delivered as intended [fidelity])	N/A, single arm
	24b	Concomitant care received during the trial for each group	N/A
Baseline data	25	A table showing baseline demographic and clinical characteristics for each group	9, 16
Numbers analysed, outcomes and estimation	26	For each primary and secondary outcome, by group:<br>● the number of participants included in the analysis<br>● the number of participants with available data at the outcome time point<br>● result for each group, and the estimated effect size and its precision (such as 95% confidence interval)<br>● for binary outcomes, presentation of both absolute and relative effect size	9, 10
Harms	27	All harms or unintended events in each group	N/A
Ancillary analyses	28	Any other analyses performed, including subgroup and sensitivity analyses, distinguishing pre-specified from post-hoc	11
**Discussion**			
Interpretation	29	Interpretation consistent with results, balancing benefits and harms, and considering other relevant evidence	7-9
Limitations	30	Trial limitations, addressing sources of potential bias, imprecision, generalisability, and, if relevant, multiplicity of analyses	9

*We strongly recommend reading this statement in conjunction with the CONSORT 2025 Explanation and Elaboration and/or the CONSORT 2025 Expanded Checklist for important clarifications on all the items. We also recommend reading relevant CONSORT extensions. See www.consort-spirit.org.

Citation: Hopewell S, Chan AW, Collins GS, Hróbjartsson A, Moher D, Schulz KF, et al. CONSORT 2025 Statement: updated guideline for reporting randomised trials. BMJ. 2025; 388:e081123. https://dx.doi.org/10.1136/bmj-2024-081123.

© 2025 Hopewell et al. This is an Open Access article distributed under the terms of the Creative Commons Attribution License (https://creativecommons.org/licenses/by/4.0/), which permits unrestricted use, distribution, and reproduction in any medium, provided the original work is properly cited.
